# Helicity-driven chiral self-sorting supramolecular polymerization with Ag^+^: right- and left-helical aggregates†

**DOI:** 10.1039/d1sc06413d

**Published:** 2022-02-09

**Authors:** Mirae Ok, Ka Young Kim, Heekyoung Choi, Seonghan Kim, Shim Sung Lee, Jaeheung Cho, Sung Ho Jung, Jong Hwa Jung

**Affiliations:** Department of Chemistry, Research Institute of Natural Sciences, Gyeongsang National University Jinju 52828 Korea jonghwa@gnu.ac.kr; Department of Chemistry, Ulsan National Institute of Science and Technology Ulsan 44919 Korea; Department of Emerging Materials Science, Daegu Gyeongbuk Institute of Science and Technology Daegu 42988 Korea; Department of Liberal Arts, Gyeongsang National University Jinju 52828 Korea

## Abstract

The study of chiral self-sorting is extremely important for understanding biological systems and for developing applications for the biomedical field. In this study, we attempted unprecedented chiral self-sorting supramolecular polymerization accompanying helical inversion with Ag^+^ in one enantiomeric component. Bola-type terpyridine-based ligands (*R*-L^1^ and *S*-L^1^) comprising *R*- or *S*-alanine analogs were synthesized. First, *R*-L^1^ dissolved in DMSO/H_2_O (1 : 1, v/v) forms right-handed helical fibers (aggregate I) *via* supramolecular polymerization. However, after the addition of AgNO_3_ (0.2–1.1 equiv.) to the *R*-L^1^ ligand, in particular, it was found that aggregate II with left-handed helicity is generated from the [*R*-L^1^(AgNO_3_)_2_] complex through the [*R*-L^1^Ag]^+^ complex *via* the dissociation of aggregate I by a multistep with an off pathway, thus demonstrating interesting self-sorting properties driven by helicity and shape discrimination. In addition, the [*R*-L^1^(AgNO_3_)_2_] complex, which acted as a building block to generate aggregate III with a spherical structure, existed as a metastable product during the formation of aggregate II in the presence of 1.2–1.5 equiv. of AgNO_3_. Furthermore, the AFM and CD results of two samples prepared using aggregates I and III with different volume ratios were similar to those obtained upon the addition of AgNO_3_ to free *R*-L^1^. These findings suggest that homochiral self-sorting in a mixture system occurred by the generation of aggregate II composed of the [*R*-L^1^Ag]^+^ complex *via* the rearrangement of both, aggregates I and III. This is a unique example of helicity- and shape-driven chiral self-sorting supramolecular polymerization induced by Ag^+^ starting from one enantiomeric component. This research will improve understanding of homochirality in complex biological models and contribute to the development of new chiral materials and catalysts for asymmetric synthesis.

## Abstract 2

Chiral self-sorting supramolecular polymerization of bola-type terpyridine-based ligands (*R*-L^1^ and *S*-L^1^) comprising *R*- or *S*-alanine analogs occurred upon addition of Ag^+^ in one enantiomeric component.

## Graphical abstract

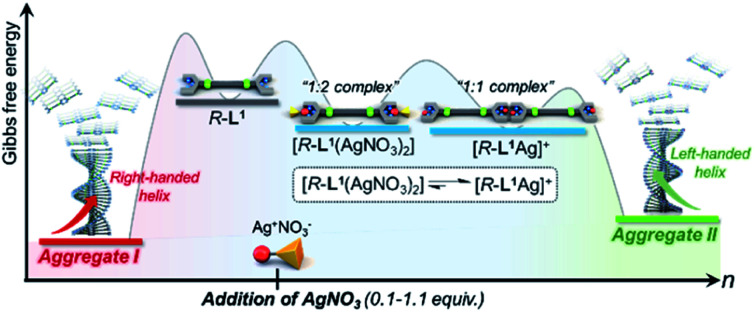

## Introduction

Chiral metal-coordinated supramolecular assemblies have recently received much attention and are among the most promising structures in supramolecular chemistry,^1–10^ because metal-coordinated systems play essential roles in the formation of superstructures such as coiled-coil helix bundle proteins,^11,12^ DNA superhelices,^13^ and protein-DNA hybrid superstructures.^14,15^ Thus, better understanding of emergent chiral phenomena and the origin of chirality at the supramolecular level are essential for biomedical applications such as drug delivery,^16–18^ gene delivery,^19^ tissue engineering,^20,21^ imaging,^22^ and sensing.^23,24^

Self-sorting is one of the high-fidelity self-recognitions or self-discriminations in living systems, in which a complex object is recognizable from non-self into well-organized supramolecular architectures.^25–28^ Biomacromolecules have great self-sorting ability, for example proteins and enzymes can distinguish two enantiomers by supramolecular interactions and thus promote biological processes.^29,30^ Chirality-driven self-sorting processes are also mainly dependent on the handedness of the helical structures with the configuration of the enantiomer moiety. A variety of artificial systems, driven by metal–ligand coordination, hydrogen bonding, and solvophobic interaction in a racemic mixture,^31–34^ have been widely investigated. In particular, metal–ligand coordination interactions are the most widely used for the self-sorted construction of discrete and functional complex structures, including chiral ones at the single crystal level.^35–39^

To the best of our knowledge, helicity-driven self-sorting supramolecular polymerization in a single component, is rarely reported.^40,41^ Recently, one example for helicity-driven self-sorting in a single component system was reported by the Wurthner group.^40^ According to the cooling rate, the achiral perylene bisimide derivative formed either a homochiral self-assembly with a columnar structure or a heterochiral self-assembly with a lamellar structure, forming supramolecular polymers. In contrast, no studies have reported helicity-driven self-sorting metallosupramolecular polymerization induced by metal ions in a single component system, because transition metal ions form a variety of coordination geometries with different coordination numbers, and one handedness of supramolecular polymers is mainly determined by chirality of organic building blocks. Thus, a study on metallosupramolecular polymerization with handedness regulation by metal ions in a single component system, as a unique self-sorting system, can offer significant insights into the underlying mechanisms to understand chirality in biological systems, though it is a major challenge.

**Fig. 1:**
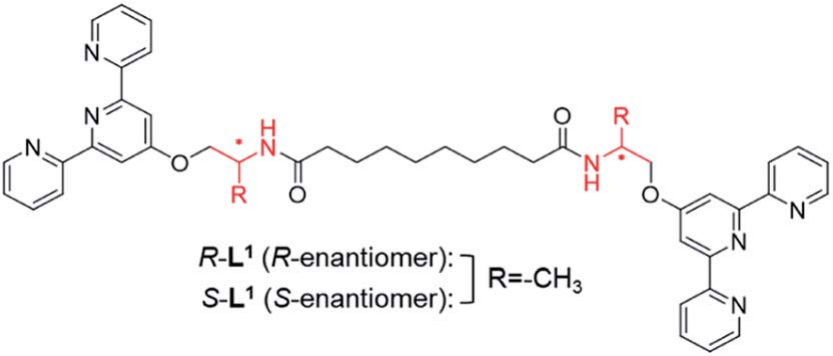
Chemical structures used in this study (*R*-L^1^ and *S*-L^1^).

We designed chiral bolaamphiphilic terpyridine-based ligands (*R*-L^1^ and *S*-L^1^), possessing either two *R*- or two *S*-alanine moieties, toward chiral self-assembly and towards intermolecular hydrogen-bonding interactions for promoting metallosupramolecular polymerization (Fig. 1 and Scheme S1†). The Ag^+^ ion was chosen as a guest ion as it allows the formation of a variety of coordination geometries, such as linear, triangular, square planar, and tetrahedral structures with different coordination numbers, which mainly depends on the concentration of Ag^+^ and structures of ligands.^42–44^ In particular, bolaamphiphilic terpyridine-based ligands form a helical network structure by coordination of each terpyridine moiety attached to two ligand molecules to one Ag^+^ ion. Ag–Ag interactions induce helical structures in complexes, and act as an additional driving force for the formation of helical metallosupramolecular architectures. Thus, herein, we report chiral self-sorting Ag^+^-coordinated supramolecular polymerization accompanied by helical inversion. Notably, the supramolecular polymerization is precisely controlled by the Ag^+^ concentration, in a unique example of chiral self-sorting through the self-helicity and self-shape recognition of free ligands (*R*-L^1^, and *S*-L^1^) (Fig. 1) and complexes ([*R*-L^1^Ag]^+^ and [*S*-L^1^Ag]^+^) from a single component, but not a racemic mixture.

## Results and discussion

### Stoichiometric ratios for Ag^+^ complexes by ESI-MS

In order to monitor the molar ratio effect on the formation of silver(i) complexes of *R*-L^1^ systemically, a series of the high-resolution electrospray ionization mass spectrometry (HR-ESI-MS) experiments were performed by varying the AgNO_3_ contents (0–2.0 equiv.). When the molar ratios are in the range of 0.1–0.4 equiv., monosilver complex [*R*-L^1^Ag]^+^ at *m*/*z* 885.3002 is predominant except the free *R*-L^1^ (Fig. S1 and S2†). Above 0.6 equiv. of AgNO_3_, a new peak appeared at *m*/*z* 497.1021, corresponding to the disilver complex [*R*-L^1^Ag_2_]^2+^ as a minor product (Fig. S3†). When the molar ratio reaches 2.0 equiv., the disilver complex becomes a major species (Fig. S4–S7†). Anion-coordinated disilver complex [*R*-L^1^Ag_2_NO_3_]^+^ is also observed (Fig. S8†). These results indicate that *R*-L^1^ forms a 1 : 2 (ligand-to-metal) complex in the presence of the excess Ag^+^.

### Morphology of chiral supramolecular polymers by AFM

**Fig. 2:**
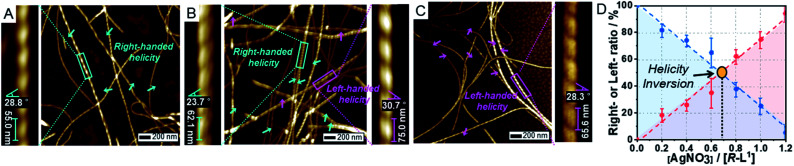
AFM images of self-assembled *R*-L^1^ with AgNO_3_ obtained in a DMSO and H_2_O mixture (1 : 1 v/v): (A) 0.0, (B) 0.2, and (C) 1.2 equiv. (D) Plot of the ratio of right- or left-handed helical fibers *vs.* equivalents of AgNO_3_. The ratio of right- to left-handed fiber was calculated by the number of individual fibers (50–100); the blue and the red colors indicate the ratio of right- and left-handed fibers, respectively.

To investigate the morphologies of the supramolecular polymers formed depending on Ag^+^ contents, we analyzed atomic force microscopy (AFM) images of *R*-L^1^ and *S*-L^1^ in the presence of AgNO_3_ (0–1.8 equiv.) in DMSO/H_2_O (1 : 1 v/v). First, *R*-L^1^ forms right-handed fibers (aggregate I) with pitch lengths and angles of 55 ± 7 nm and 28 ± 3°, respectively (Fig. 2A and Table S1†). The height of aggregate I was determined to be 8.0 nm (Fig. S9†), which is slightly larger than twice the molecular length of *R*-L^1^ indicating a bilayer structure. The helicity of aggregate I is consistent with that of the *R*-alanine moiety,^7,45^ which implies that the helicity of the self-assembled nanostructures is derived from the chirality of their molecular building blocks. No morphological changes were observed after aging for 24 h.

In the presence of AgNO_3_ (0.2 equiv.), to our surprise, both right- and left-handed helical fibers were observed, indicating the coexistence of two building blocks at a molecular level. The left-handed helical fiber (aggregate II) had a pitch length of ∼75 ± 5 nm and a pitch angle of ∼30 ± 3° (Fig. 2B and Table S1†). However, aggregate I was the major product with less than 0.7 equiv. of AgNO_3_ (Fig. 2D). These results indicate that aggregate I originates from *R*-L^1^, whereas aggregate II originates from the [*R*-L^1^Ag]^+^ complex accompanied by the inversion of helicity. This chiral self-sorting is induced by self-discrimination in a mixture of building blocks of different shapes and handedness. Thus, AgNO_3_ induced chiral self-sorting for *R*-L^1^, the first such example of self-sorting from a single enantiomeric component. With more than 0.8 equiv. of AgNO_3_, aggregate II emerged as the dominant structure. With increasing AgNO_3_ concentration, the concentration of aggregate I gradually reduced, whereas that of aggregate II gradually increased with no change in the pitch length and pitch angle (Fig. 2 and S10–S16†).

Subsequently, only aggregate II was observed with more than 1.2 equiv. of AgNO_3_. No further morphological change was observed after one month of incubation, indicating that aggregate II based on [*R*-L^1^Ag]^+^ was the thermodynamic product. The ratio of aggregates I to II was determined by the number of individual fibers (50–100) (Fig. 2D and S10–S16†). Interestingly, the concentration of aggregate II increased with increasing Ag^+^ concentration (0–1.2 equiv.), as shown in Fig. 2D. In addition, the increased tendency of the concentration of aggregate II was consistent with the HR-ESI-MS results (Fig. S17†), because aggregate II was produced by the [*R*-L^1^Ag]^+^ complex. Thus, chiral self-sorting in this supramolecular polymerization can be controlled quantitatively using the concentration of AgNO_3_. In addition, the AFM image revealed a spherical structure (aggregate III) in the presence of AgNO_3_ (2.0 equiv.) (Fig. S18†), which originated from the self-assembled [*R*-L^1^(AgNO_3_)_2_] complex. The right-handed helicity of the free ligand based on bipyridine possessing the alanine moiety was converted to the left-handed helicity upon addition of Ag^+^ by circular dichroism (CD) observation in our previous study.^6^ However, AFM revealed one-handed helicity of the self-assemblies although the Ag^+^ concentration revealed one handedness.^6^ Thus, the presence of isolated right – and left-handed helical fibers induced by Ag^+^ in one sample is a unique phenomenon, which indicates a new chiral self-sorting system that occurs due to free ligands and complexes with opposite helicities in a single enantiomeric component.

AFM images of the self-assembled *S*-L^1^ ligand with and without AgNO_3_ revealed the opposite behavior to that exhibited by the self-assembled *R*-L^1^ with and without AgNO_3_. In the absence of AgNO_3_, the self-assembled *S*-L^1^ ligand formed a left-handed helical fiber with a pitch length of ∼58 ± 7 nm and a pitch angle of ∼30 ± 5° (Fig. S19†). This left-handed helicity is consistent with the molecular chirality of the enantiomeric alanine moiety embedded in *S*-L^1^,^7,45^ indicating that the helicity of the supramolecular polymer was determined by the *S*-alanine moiety of *S*-L^1^. Chiral self-sorting was also observed upon the addition of AgNO_3_. Increasing AgNO_3_ concentration induced the formation of right-handed helical fibers (Fig. S19–S25†); this result was attributed to the formation of the [*S*-L^1^Ag]^+^ complex and the accompanying helical inversion.

By employing a 1 : 1 mixture of *R*-L^1^ and *S*-L^1^, we also observed chiral self-sorting *via* the supramolecular polymerization with and without Ag^+^. Without Ag^+^, for example, the right- and left-handed fibers with approximately 1 : 1 ratio (51.9 : 48.1, Fig. S26†) were formed due to the homochiral (or *narcissistic*) self-sorting during supramolecular polymerization. In the presence of 0.6 equiv. of Ag^+^, again right- and left-handed fibers with approximately 1 : 1 ratio (52.1 : 47.9, Fig. S27†) were formed. In this case, however, there are two sources of the right-handed fibers: *R*-L^1^ and [*S*-L^1^Ag]^+^, due to the helicity inversion upon silver(i) complexation. Similarly, the left-handed fibers originates from *S*-L^1^ and [*R*-L^1^Ag]^+^. These results demonstrate that chiral self-sorting occurs by selective shape-recognition *via* helicity inversion upon silver(i) complexation: *R*-L^1^ and [*S*-L^1^Ag]^+^ form a *P*-type helical structure (or *S*-L^1^ and [*R*-L^1^Ag]^+^ form an *M*-type helical structure).

### Helical changes of supramolecular polymers by CD

**Fig. 3:**
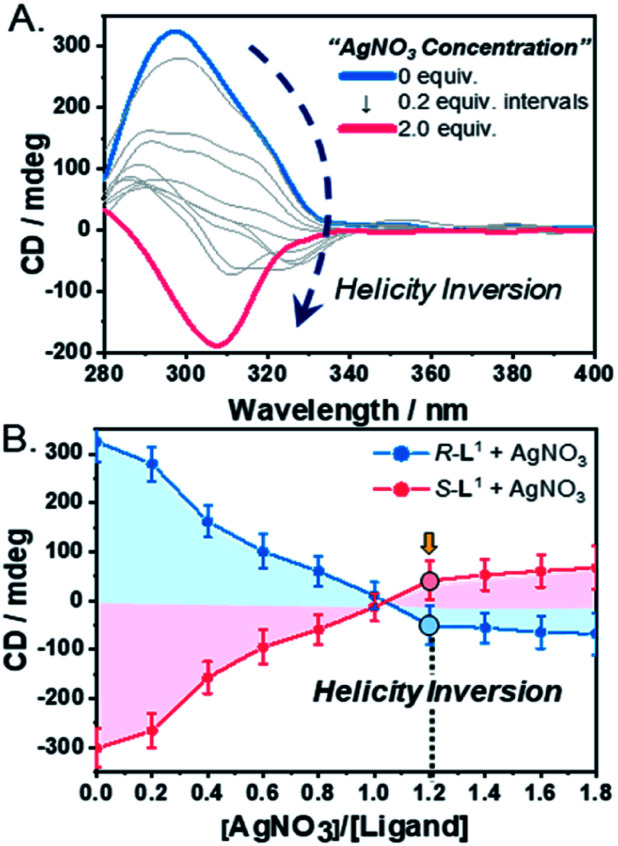
(A) CD spectra of *R*-L^1^ (8 mM) upon addition of AgNO_3_ in a DMSO and H_2_O mixture (1 : 1 v/v). (B) Plot of CD spectra intensities of *R*-L^1^ and *S*-L^1^*vs.* equivalents of AgNO_3_ in a H_2_O and DMSO mixture (1 : 1 v/v). These spectra were determined using a special quartz cell with 0.1 mm of the path length (free ligand: Δ*ε* = *ca.* 1.1 × 10^4^).

To demonstrate self-sorting and helicity inversion upon the addition of AgNO_3_, CD spectra of self-assembled *R*-L^1^ and *S*-L^1^ in a DMSO and H_2_O mixture (1 : 1 v/v) were recorded. An intense positive CD signal, corresponding to the terpyridine moiety of the free ligand *R*-L^1^, was observed at ∼297 nm (Fig. 3A). A positive CD signal indicates that the *R*-L^1^ ligand is inclined toward *P*-helicity (right-handed),^7,8,45^ which is consistent with the AFM observations. In contrast, the intensity of the CD signal for self-assembled *R*-L^1^ gradually decreased with the concentration of AgNO_3_, and eventually became negative with 1.2 equiv. of AgNO_3_ (Fig. 3B), when the CD signal exhibited a red shift of ∼10 nm relative to the signal of the self-assembled free *R*-L^1^ ligand (no AgNO_3_). The reduction of the positive CD signal occurred due to the coexistence of aggregate I with right-handed (*P*-type) helicity and aggregate II with left-handed (*M*-type) helicity,^7,8,45^ as confirmed by AFM observations. The negative signal at ∼307 nm originated from the terpyridine–Ag^+^ in the [*R*-L^1^Ag]^+^ complex, which is not a mirror image of the free ligand *R*-L^1^. This result indicates that the [*R*-L^1^Ag]^+^ complex exhibited left-handed helicity. Thus, the addition of Ag^+^ to the free *R*-L^1^ ligand produced building blocks with opposite handedness. These helical building blocks aggregated with those exhibiting the same handedness and shapes, resulting in helicity- and shape-driven chiral self-sorting supramolecular polymerization with Ag^+^. In conclusion, the addition of Ag^+^ to the free *R*-L^1^ ligand (right-handed helicity) induced unique chiral self-sorting through the formation of the [*R*-L^1^Ag]^+^ complex, which has left-handed helicity.

To confirm the linear dichroism (LD) effect in the CD signal, we also observed the LD spectra of *R*-L^1^ in the absence and the presence of AgNO_3_ (1.2 and 2.0 equiv.) under the same conditions as those used for the CD observations (Fig. S28†). The intensities of the LD spectrum for a solution of *R*-L^1^ in the absence and the presence of AgNO_3_ (1.2 and 2.0 equiv.) were negligible, indicating that the LD effect did not contribute to the CD signals of the helical supramolecular polymers.

In contrast, the CD spectra of self-assembled *S*-L^1^ with and without AgNO_3_ were completely opposite to those of self-assembled *R*-L^1^ without and with AgNO_3_ (Fig. 3B and S29†). These CD measurements indicate that chiral self-sorting in supramolecular polymerization based on *S*-L^1^ with AgNO_3_ occurred due to helicity inversion when the free *S*-L^1^ ligand (left-handed *M*-type helicity) was converted to the [*S*-L^1^Ag]^+^ complex (right-handed *P*-type helicity).

### Mechanism for chiral self-sorting supramolecular polymerization

**Fig. 4:**
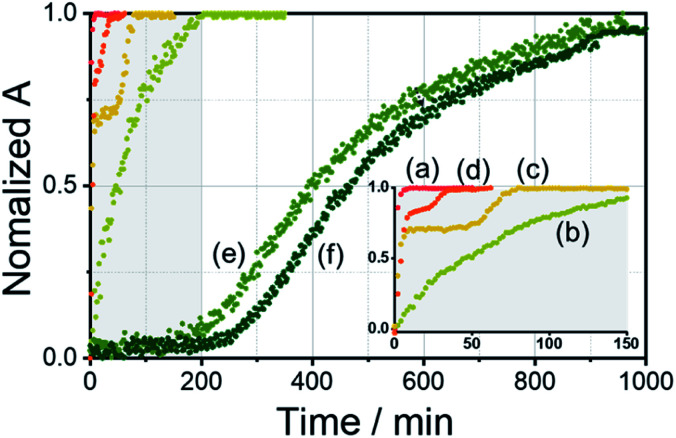
Time-dependent UV-Vis spectral changes of *R*-L^1^ (7.2 mM) with AgNO_3_ in a mixture of H_2_O and DMSO (1 : 1 v/v); (a) 0 equiv., (b) 0.4 equiv., (c) 0.6 equiv., (d) 0.8 equiv., (e) 1.2 equiv., and (f) 1.4 equiv.

**Fig. 5:**
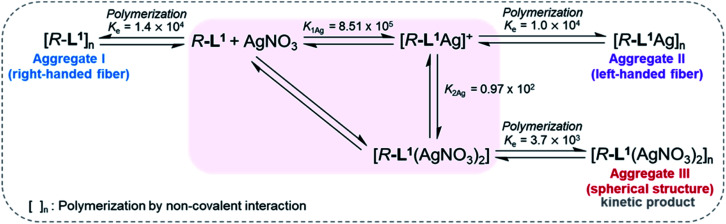
Proposed chiral self-sorting supramolecular polymerization of *R*-L^1^ at different concentrations of AgNO_3_ (the formation of aggregate II with left-helicity and aggregate III with left-helicity). The stability constants (*K*_1Ag_ and *K*_2Ag_) were determined by UV-Vis titration using a previously reported method.^48–50^

To demonstrate self-sorting in supramolecular polymerization, time-dependent UV-Vis spectra of the self-assembled *R*-L^1^ were obtained in the presence and the absence of AgNO_3_ in a mixture of DMSO and H_2_O (1 : 1 v/v) (Fig. 4 and S30–S32†). The absorbance of the free ligand *R*-L^1^ increased quickly without AgNO_3_ and the system reached equilibrium within 10 min (Fig. 4a), which indicates the absence of a metastable supramolecular polymerization product in the formation of aggregate I (Fig. 5). With 0.4 equiv. of AgNO_3_, the absorbance increased more slowly than that without AgNO_3_ (Fig. 4b). This was attributed to the formation of aggregates I and II by two different pathways with the competition reaction (Fig. 5). This phenomenon can be described by stability constants (*K*_1Ag_ and *K*_2Ag_) obtained from the control experiment (Fig. S33†) and elongation binding constant (*K*_e_), as described by Meijer and Takeuchi groups in porphyrin-based supramolecular polymerization with Zn^2+^ and pyridine.^46,47^ Based on the ESI-MS results (Fig. S2†), stability and elongation constants (Fig. S33†), we assumed that the free *R*-L^1^ ligand formed a supramolecular polymer, and then the partial aggregate I then dissociated to the monomeric species to form the [*R*-L^1^Ag]^+^ complex by the equilibrium reaction (Fig. 5), because the complex formation of Ag^+^ in the monomeric species (*R*-L^1^) state is more thermodynamically favorable than that in the aggregate I state in the present system. The free ligand *R*-L^1^ is capable of forming the [*R*-L^1^Ag]^+^ complex spontaneously based on the stability constant (*K*_1Ag_ = 8.51 × 10^5^) obtained from UV-Vis titration.^48–50^ Finally, the monomer [*R*-L^1^Ag]^+^ complex formed a supramolecular polymer. More interestingly, two step-shaped curves were observed with 0.6 and 0.8 equiv. of AgNO_3_ (Fig. 4c and d). The absorbance increased quickly at the initial stage, and a lag time was observed after aging for 50 min and 20 min in the presence of 0.6 and 0.8 equiv. for AgNO_3_, respectively. These phenomena were caused by the free *R*-L^1^ ligand generating aggregate I quickly without a metastable state in the initial stage, and the dissociation of a small quantity of aggregate I to monomer species upon the addition of Ag^+^. The monomer species formed the [*R*-L^1^Ag]^+^ and [*R*-L^1^(AgNO_3_)_2_] complexes, as confirmed by HR-ESI-MS observations (Fig. S2†), and then generated aggregate II based on the [*R*-L^1^Ag]^+^ complex (Fig. 5), because the stability constant (*K*_1Ag_ = 8.51 × 10^5^) of [*R*-L^1^Ag]^+^ was higher than the stability constant (*K*_2Ag_ = 0.97 × 10^2^) of the [*R*-L^1^(AgNO_3_)_2_] complex. The lag times at 0.6 and 0.8 equiv. of AgNO_3_ were due to the intermediate aggregate I. However, the [*R*-L^1^(AgNO_3_)_2_] complex formed in the presence of AgNO_3_ (0.5–1.1 equiv.) did not form aggregate III with a spherical structure, according to AFM observations (Fig. S13–S15†), because the concentration of the [*R*-L^1^(AgNO_3_)_2_] complex was not sufficiently high to induce nucleation. In contrast, the lag times in the self-assembly process with 1.2 and 1.4 equiv. of AgNO_3_ were clearly observed in the initial stage (Fig. 4e and f). The AFM image showed a spherical structure with a diameter of 100–300 nm after incubation for 1 h (Fig. S34A†), which originated from the [*R*-L^1^(AgNO_3_)_2_] complex (Fig. 5), as confirmed by HR-ESI-MS observations (Fig. S35†). These findings indicate that aggregate II, based on the [*R*-L^1^Ag]^+^ complex, was formed *via* the metastable intermediate aggregate III, based on the [*R*-L^1^(AgNO_3_)_2_] complex, upon the addition of 1.2 and 1.4 equiv. of AgNO_3_. Thus, aggregate II, which is based on the [*R*-L^1^Ag]^+^ complex, was the thermodynamically favored product. The AFM image also showed one-dimensional nanorods with a length of 500–800 nm and nanoparticles 100–300 nm in diameter (Fig. S34B and C†), which were formed due to the coexistence of aggregates II and III. After aging for 3 days, the nanorods grew into left-handed fibrous structures (Fig. S34D†). This clearly demonstrated that the [*R*-L^1^Ag]^+^ complex grew into one-dimensional fibers through intermolecular interactions. Thus, the long lag time was attributed to the existence of aggregate III comprising the [*R*-L^1^(AgNO_3_)_2_] complex as a metastable product.

We also measured the time-dependent CD spectra of *R*-L^1^ with and without AgNO_3_ (Fig. S36†). Although the CD spectral changes of *R*-L^1^ in the presence of 0.4 and 0.8 equiv. of AgNO_3_ were observed, the lag times were not obtained because both right- and left-handed helical fibers were present. In the presence of 1.2 and 1.4 equiv. of AgNO_3_, we could observe the lag time attributed to the formation of aggregate III as an intermediate.

### Chiral self-sorting supramolecular polymerization by rearrangement in aggregates

**Fig. 6:**
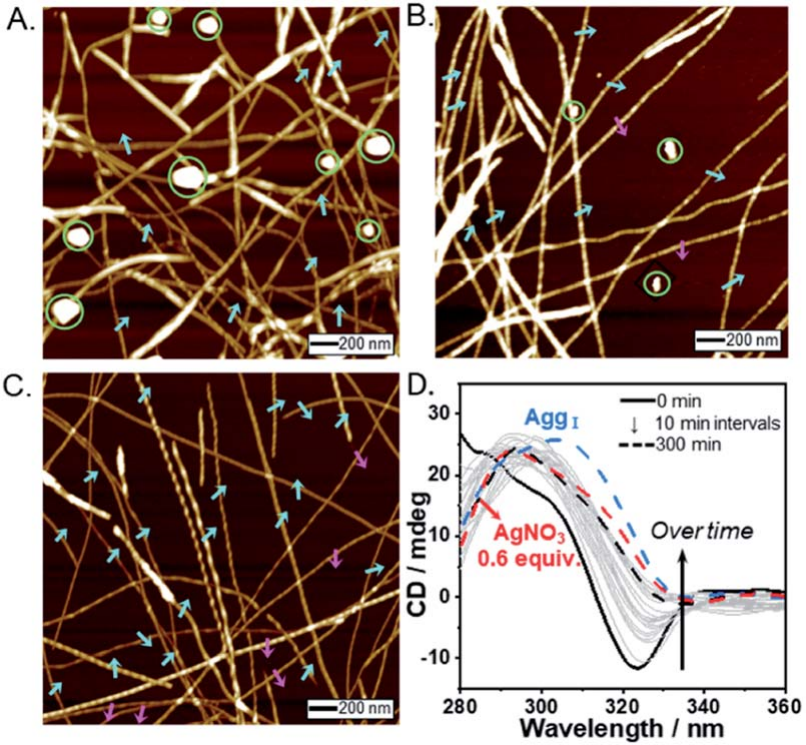
AFM images of a mixed sample of aggregate I (7.2 mM, 50 μL) and aggregate III (7.2 mM, 21.5 μL) after aging time of (A) 10 min, (B) 1 h, and (C) 3 h. The molar ratio of Ag^+^ to *R*-L^1^ in a mixed sample was 0.6 equiv. (D) Time-dependent CD spectra of a mixed sample of aggregate I (7.2 mM, 50 μL) and aggregate III (7.2 mM, 21.5 μL) in a mixture of DMSO and H_2_O (1 : 1 v/v). The blue and pink arrows indicate right- and left-handed structures, respectively. The green circles indicate a spherical structure.

To prove that self-sorting occurred by the rearrangement of building blocks, we observed time-dependent AFM images of self-assemblies prepared under three different conditions (Fig. 6). We prepared two samples (Ag^+^ : *R*-L^1^ molar ratios = 0.6 and 1.0 equiv.) using different volume ratios of aggregates I and III. With 0.6 equiv. AgNO_3_, as expected, the AFM image clearly revealed a right-handed helical fiber (aggregate I) and a spherical structure (aggregate III) in the initial stage (Fig. 6A). After aging for 1 h, fewer spherical structures and left-handed helical fibers (aggregate II) were observed (Fig. 6B). Finally, the AFM image showed right- and left-handed helical fibers at an approximate ratio of 65 : 35 (Fig. 6C and S37†), which were similar to that obtained upon the addition of 0.6 equiv. AgNO_3_ to free *R*-L^1^ (Fig. 2D). In addition, a positive CD signal was observed when aggregate I was added to aggregate III (Fig. 6D). Then, the mixed solution reached equilibrium in 3 h. This result is similar to that obtained upon the addition of 0.6 equiv. of AgNO_3_ to free *R*-L^1^. These findings suggest that chiral self-sorting in a mixture system occurred by the generation of aggregate II comprising the [*R*-L^1^Ag]^+^ complex *via* the rearrangement of aggregates I and III. In a solution consisting of 1.0 equiv. AgNO_3_, both a sphere and right-handed fiber were observed in the initial state (Fig. S38C†). Remarkably, after 1 h, the AFM image showed aggregate I as the major product and aggregate II as the minor product (Fig. S38D†). After 1 day, aggregates I and II (*ca.* 20 : 80) were obtained (Fig. S38E†), and no further morphological changes were observed after 5 days. Aggregate II was generated through the dissociation of partial aggregate I to form the [*R*-L^1^Ag]^+^ complex. These results clearly demonstrate that Ag^+^ acts as a key component in chiral self-sorting supramolecular polymerization. The mixed solution produced a negative CD signal, and it reached equilibrium after 13 h. This also demonstrates that aggregate II was generated by rearrangement in a mixture of aggregates I and III (Fig. S38F†). In comparison to the negative CD signal shown in Fig. 6D, the more negative CD signal (Fig. S38F†) was due to the formation of large amount of aggregate II.

The temperature-dependent changes in the CD spectra of the two samples prepared from a mixture of aggregates I and III, resulting in samples consisting of 0.6 and 1.2 equiv. of AgNO_3_, respectively, were almost the same as those obtained upon addition of AgNO_3_ to the free *R*-L^1^ (Fig. S39†), indicating that the self-sorting effect triggered by a mixture of aggregates I and III is the same as that for a mixture of the free *R*-L^1^ ligand and AgNO_3_. In addition, AgNO_3_ (1.2 equiv.) was added to aggregate I to confirm the self-sorting mechanism for the generation of aggregate II. As shown in Fig. S40,† a negative CD signal was observed, but no positive signals were observed when 1.2 equiv. of AgNO_3_ was added to aggregate I formed without AgNO_3_, because aggregate I dissociated quickly to the monomer species, and then formed [*R*-L^1^Ag]^+^ and [*R*-L^1^(AgNO_3_)_2_] (Fig. 5). The initial negative CD signal was observed due to the formation of aggregate III as a kinetic product, and this signal did not change during the first 300 min (Fig. S40B†); this was attributed to the existence of the metastable aggregate III based on the [*R*-L^1^(AgNO_3_)_2_] complex. After aging for 300 min, the negative CD signal gradually increased and finally reached equilibrium after 1200 min (Fig. S40B†). This was attributed to the conversion of aggregate III into aggregate II, as observed by AFM (Fig. S34†). The occurrence of the negative CD signal also indicates that aggregate II existed as the dominant product.

### Study of intermolecular interactions in supramolecular polymerization

To better understand the intermolecular structures involved in supramolecular polymerization, we analyzed the chiral self-sorting supramolecular polymers by Fourier transform infrared (FTIR) spectroscopy. Compared to the spectrum of the monomer *R*-L^1^, the spectra of aggregates I and II showed a shift in the –NH stretching bands to shorter wavenumbers (Fig. S41A and B(c)†), which confirmed the presence of intermolecular hydrogen bonds between the amide groups of the alanine moieties. The amide I band also shifted to shorter wavenumbers. The shift of the –NH and amide I bands of aggregate I was larger than that of aggregate II (Fig. S41B†), indicating that the intermolecular hydrogen-bonding interactions in aggregate I were stronger than those in aggregate II. The peaks at 1335 cm^−1^ (pink) and 1355 cm^−1^ (gray, broad, Fig. S41B(c)†) originate from the uncoordinated NO_3_^−^ and the self-assembled [*R*-L^1^Ag]^+^, respectively.^51^ In contrast, the NO_3_^−^ band of aggregate III shifted to a shorter wavenumber, which is indicative of coordination to Ag^+^.

In addition, temperature-dependent ^1^H NMR spectra of the two supramolecular polymers were observed by heating (Fig. S42 and S43†). The aromatic proton peaks of terpyridine with AgNO_3_ (1.2 equiv.) largely shifted to a low field compared to that without AgNO_3_, indicative of the π–π stacking of *R*-L^1^ in the presence of AgNO_3_. In contrast, the chemical shift of the aromatic proton peaks without AgNO_3_ is negligible, which means that the π–π stacking of aggregate I was negligible.^52,53^ Also, the aromatic proton peaks of *R*-L^1^ with AgNO_3_ are broader than those of the free *R*-L^1^, mainly due to the formation of the [*R*-L^1^Ag]^+^ complex and the supramolecular polymerization.

Moreover, the wide-angle X-ray diffraction (WAXD) measurements of self-assembled samples obtained with AgNO_3_ (0.5, 0.8, and 1.2 equiv.) revealed a sharp peak, which corresponds to the Ag–Ag interaction with a distance of 2.8 Å, at 2*θ* = 32° (Fig. S44†).^54,55^ This peak was distinct with 1.2 equiv. of AgNO_3_. The Ag–Ag interaction likely plays a key role not only in the formation of left-handed aggregate II, but also as an additional driving force. In contrast, no two WAXD patterns were observed at 2*θ* = 32° for aggregate I prepared without AgNO_3_ and aggregate III obtained with 2.0 equiv. of AgNO_3_. These results indicate the absence of Ag–Ag interactions (Fig. S45†).

### DFT calculations

**Fig. 7:**
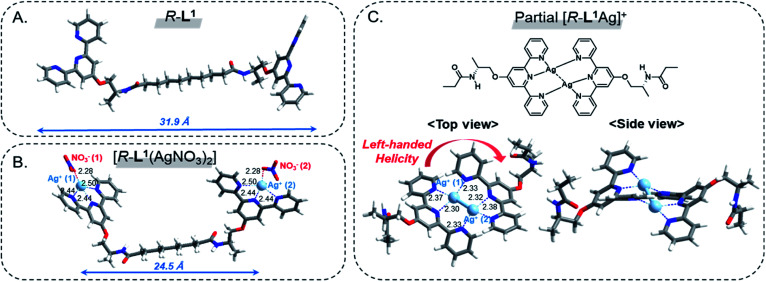
DFT-optimized structures; (A) *R*-L^1^, (B) [*R*-L^1^(AgNO_3_)_2_] and (C) partial [*R*-L^1^Ag]^+^ viewed from the side and top. The local coordination geometry and bond lengths are displayed.

To investigate the local coordination geometry around Ag^+^, we optimized the molecular structure of the free ligand *R*-L^1^, and the [*R*-L^1^(AgNO_3_)_2_] complex using density functional theory (DFT) calculations.^56^ The optimized molecular length of the free ligand *R*-L^1^ was 31.9 Å, and the dihedral angle between the two terpyridine moieties of *R*-L^1^ was approximately 46.8° (Fig. 7A, S46A and Table S2†). In contrast, the distance between Ag^+^ and the Ag^+^ of the [*R*-L^1^(AgNO_3_)_2_] complex (24.5 Å) was lower than that of the free ligand, and the dihedral angle between terpyridine and terpyridine moieties decreased to 38.7°. In addition, each Ag^+^ interacted with the three nitrogen atoms from terpyridine and one oxygen atom from NO_3_^−^, which resulted in a four-coordinated structure (Fig. 7B, S46B and Table S3†).^57,58^

For the [*R*-L^1^Ag]^+^ complex, a terpyridine derivative with alanine and methyl groups was employed instead of the [*R*-L^1^Ag]^+^ complex for simplicity. Because NO_3_^−^ in the [*R*-L^1^Ag]^+^ complex did not coordinate to Ag^+^ in FT IR observation, NO_3_^−^ was ignored in DFT calculations. As shown in Fig. 7C, S46C and Table S4,† Ag^+^ (1) is coordinated to one nitrogen atom (N1) from the left side of the terpyridine moiety and two nitrogen atoms (N5 and N6) from the right side of the terpyridine moiety. In contrast, Ag^+^ (2) is coordinated to two nitrogen atoms (N2 and N3) from the left side of the terpyridine moiety and one nitrogen atom (N4) from the right side of the terpyridine moiety. These results led to the left-handed helical structure. The distance between Ag^+^ and nitrogen atoms was 2.32–2.38 Å. More interestingly, the length of the Ag(1)–Ag(2) bond was 2.82 Å, which was consistent with the WAXD results. The result indicates that the Ag–Ag interaction acted as an additional driving force in the formation of aggregate II. The Ag^+^ (1) and Ag^+^ (2) atoms showed a four-coordinated tetrahedral structure involving a Ag–Ag interaction.

### Thermodynamic study of supramolecular polymers

**Fig. 8:**
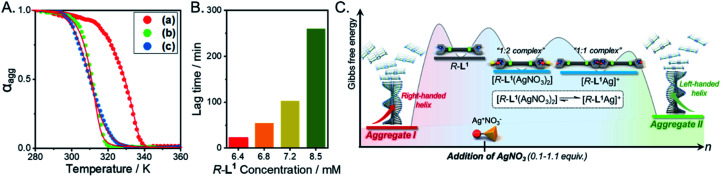
(A) Degree of aggregation at 326 nm as a function of temperature with the heating curve fitted to the equilibrium (EQ) model regime based on the nucleation–elongation model;^58–60^ (a) aggregate I, (b) aggregate II, and (c) aggregate III. (B) Representation of the lag time concentration in the presence of 0.6 equiv. AgNO_3_ in a mixture of DMSO and H_2_O (1 : 1 v/v). (C) Energy landscapes of the two competing pathways of *R*-L^1^ in the presence of AgNO_3_: elongation Gibbs free energies were experimentally determined by using the cooperative supramolecular polymerization model.

To determine the thermodynamic parameters of the chiral self-sorting supramolecular polymers, the temperature-dependent UV-Vis spectral changes were measured (from 10 to 90 °C, a heating rate of 1 K min^−1^); the temperature range was chosen based on the slow complex formation at 10 °C. The plots of absorbance *vs.* temperature for aggregates I and II exhibited non-sigmoidal curves (Fig. 8A), which indicate that both aggregate types were generated by a cooperative pathway with a nucleation–elongation mechanism.^59–61^ The aggregate I based on the free ligand *R*-L^1^ was not found in the hysteresis of heating and cooling curves (Fig. S47A†), indicating that no kinetic product existed. During heating and cooling, the *T*_e_ values are 327.2 and 325.7 K, respectively while the *T*_m_ values are 317.5 and 316.9 K, respectively. In contrast, the hysteresis of aggregate II was observed during its assembly from monomeric [*R*-L^1^Ag]^+^ upon cooling (Fig. S47B†), indicating that [*R*-L^1^Ag]^+^ monomers were trapped in a metastable state with a high-energy barrier. The thermodynamic parameters for the formation of aggregates I, II, and III were obtained by the equilibrium (EQ) model based on the nucleation–elongation model (Fig. 8A, S48 and Table S5†).^59–62^ At 7 mM, the elongation enthalpy (Δ*H*_e_) released during self-assembly and the elongation binding constant (*K*_e_) were determined to be in the range of −89.6 to −171.8 kJ mol^−1^ and 1.4 × 10^4^ to 3.7 × 10^3^ mol^−1^, respectively. Although the Δ*H*_e_ value of aggregate II was smaller than that of aggregate III, Δ*S* of aggregate II was larger than that of aggregate III, because uncoordinated NO_3_^−^ and free anions in aggregate II induced an increase of entropy. Thus, the Δ*G*_e_ value of aggregate II (−20.3 kJ mol^−1^) was larger than that of aggregate III (−17.5 kJ mol^−1^) (Fig. 8A and Table S5†), indicating that aggregate II is a thermodynamic product (Fig. 8C).

We additionally performed the kinetic experiment to propose the off-pathway. In the presence of 0.6 equiv. of AgNO_3_, the lag times were extended with increasing concentration of aggregate I (Fig. 8B and S49†). Accordingly, we assumed that the metastable aggregate I is initially formed as an off-pathway intermediate. This is also consistent with our hypothesis.

We propose an energy landscape consisting of two competing pathways to confirm the mechanism of self-assembly of *R*-L^1^ in the presence of AgNO_3_ (0.1–1.1 equiv., Fig. 8C), which induces chiral self-sorting. In the pre-equilibrium state, a mixture of *R*-L^1^ and AgNO_3_ (0.1–1.1 equiv.) may generate three species: free *R*-L^1^, [*R*-L^1^Ag]^+^, and [*R*-L^1^(AgNO_3_)_2_] (Fig. 5). As mentioned, free *R*-L^1^ forms aggregate I preferentially. Following this, partial aggregate I is converted to the monomeric complexes including [*R*-L^1^Ag]^+^ and [*R*-L^1^(AgNO_3_)_2_] *via* the stepwise complexation equilibria. During the formation of aggregate II, [*R*-L^1^(AgNO_3_)_2_] is converted to [*R*-L^1^Ag]^+^ because the monosilver complex (*K*_1Ag_ = 8.51 × 10^5^) is thermodynamically more stable than the disilver one (*K*_2Ag_ = 0.97 × 10^2^). Finally, aggregate II is generated by a multistep off-pathway.^63,64^

## Conclusions

In conclusion, we demonstrated unprecedented successive chiral self-sorting in supramolecular polymerization accompanied by helical inversion with Ag^+^ for a single enantiomeric component. Chiral self-sorting occurred by three different completion reaction pathways, which were largely affected by the Ag^+^ concentration. The chiral self-sorting supramolecular polymerization was precisely controlled with the addition of Ag^+^. The unique chiral self-sorting occurred because of the coexistence of *R*-L^1^ with right-handed helicity and the [*R*-L^1^Ag]^+^ complex with left-handed helicity. Thus, the present chiral self-sorting was induced by self-discrimination in a mixture of differently shaped building blocks with different helicities. In addition, chiral self-sorting in a mixture system occurred by the generation of aggregate II composed of the [*R*-L^1^Ag]^+^ complex *via* the rearrangement of aggregates I and III. Two different helical aggregates I and II were thermodynamically favored, and the chiral self-sorted supramolecular polymers were generated by a cooperative pathway with a nucleation–elongation mechanism. With 0.1–1.1 equiv. of AgNO_3_, the chiral self-sorted supramolecular polymer was formed through an off-pathway mechanism. This system is a significant example of chiral self-sorting supramolecular polymerization starting with one enantiomeric component and Ag^+^. We believe that this approach will contribute significantly to the development of catalysts for asymmetric synthesis and new materials with intriguing chiral properties. It will also improve understanding of the origin of homochirality in complex biological molecules.

## Author contributions

M. O. and K. Y. K. contributed equally. M. O. and K. Y. K. contributed to the design and performed the preparation and characterization of supramolecular polymers. H. C. contributed to the thermodynamic studies. S. K. and J. C. carried out DFT calculations of complexes. J. H. J. and S. H. J. directed the project. All authors contributed to the discussion and the preparation of the paper.

## Conflicts of interest

There are no conflicts to declare.

## Supplementary Material

SC-013-D1SC06413D-s001
