# Supplementary material for: Helicity-driven chiral self-sorting supramolecular polymerization with Ag+: right- and left-helical aggregates
Source: Chem Sci. 2022 Feb 9;13(11):3109–17. doi: 10.1039/d1sc06413d (PMC8926169; doi:10.1039/d1sc06413d)
Supplement: SC-013-D1SC06413D-s001 [file SC-013-D1SC06413D-s001.pdf]

Electronic Supplementary Material (ESI) for Chemical Science.
This journal is © The Royal Society of Chemistry 2022

Electronic Supplementary Information

Helicity-driven chiral self-sorting supramolecular polymerization with Ag^+: right- and left-helical aggregates

Mirae Ok[a,e], Ka Young Kim,[a,e], Heekyoung Choi[a], Seonghan Kim[b,c], Shim Sung Lee,[a] Jaeheung Cho*[b], Sung Ho Jung*[d] and Jong Hwa Jung*[a]

[a]*Department of Chemistry and Research Institute of Natural Sciences Gyeongsang National University, Jinju 52828, Korea*

[b]*Department of Chemistry, Ulsan National Institute of Science and Technology, Ulsan 44919, Korea*

[c]*Department of Emerging Materials Science, Daegu Gyeongbuk Institute of Science and Technology, Daegu 42988, Korea*

[d]*Department of Liberal Arts, Gyeongsang National University, Jinju 52828, Korea*

[e]*These authors are contributed equally.*

Contents

1. Method

1.1 General characterization ··· S3

1.2 AFM observation ··· S3

1.3 Circular dichroism studies ··· S3

1.4 Calculation of thermodynamic parameter ··S3

1.5 Preparation of silver complexes···S4

1.6 Calculation of complex stability constants···S4

1.7 Theoretical calculations··S4

2. Synthesis and characterization

2.1 Synthesis of *R*-$\mathbf{L}^2$ and *S*-$\mathbf{L}^2$···S4

2.2 Synthesis of *R*-$\mathbf{L}^1$ ··S5

2.3 Synthesis of *S*-$\mathbf{L}^1$ ··S5

3. Supplementary scheme and figures

3.1 Scheme S1·· S6

3.2 Figures S1-S49 ·· S6

3.3 Tables S1-S5·· S40

4. Analytical data

4.1 ^{1}H- and ^{13}C-NMR spectoscopy···S45

4.2 HR mass spectrometry··S47

5. Supplementary references·· S48

Supplementary data

1. Methods

1.1 General characterization: The ^{1}H and ^{13}C NMR spectra were taken on a Bruker DRX 300, and Bruker DRX 500. Mass spectroscopy samples were analyzed on a JEOL JMS-700 mass spectrometer. The high resolution mass spectra (HR-MS) were measured by electrospray ionization (ESI) with a micro TOF Focus spectrometer from SYNAPT G2 (Waters, U.K.). A UV-visible spectrophotometer (JASCO J-815) was used to obtain the absorption spectra. IR spectra were observed over the range 500-4000 cm^{-1}, with a Thermo scientific Nicolet iS 10 instrument. Powder X-ray pattern (PXRD) was recorded on a Rigaku model NANOPIX X-ray diffractometer with a Cu K_{α} radiation source.

1.2 AFM observation: Atomic force microscope (AFM) imaging was performed by using XE-100 and a PPP-NCHR 10 M cantilever (Park systems). The AFM samples were prepared by spin-coating (2000 rpm) onto freshly cleaved Muscovite Mica, and images were recorded with the AFM operating in noncontact mode in air at RT with resolution of 1024 × 1024 pixels, using moderate scan rates (0.3 Hz). AFM images were recorded for fibers obtained from different ratio of right- and left-handed helix at diverse Ag^{+} equivalents. In each image, 50~100 fibers were selected from different regions of the mica and analyzed using XEI software developed by Park systems.

1.3 Circular dichroism (CD) and UV-vis studies: The CD and UV-vis spectra were recorded on a Jasco J-815 CD spectrophotometer. The CD and UV-vis spectra were determined over the range of 200-500 nm using a quartz cell with 0.1 mm path length. Scans were taken at rate of 200 nm/min with a sampling interval of 0.5 nm and response time of 0.5 s. To elucidate the supramolecular polymerization process, we first prepared the sample by dissolving *R*-$\mathbf{L^1}$ (7.2 mM) with or without $AgNO_3$ in H_2O/DMSO (1:1 v/v). After adding the sample to the CD and UV cells, it was heated to 90 °C (1 °C/min) to form the monomeric species in CD and UV-vis spectroscopy. Then the sample was cooled to 20 °C (5 °C/min) in UV-vis spectroscopy. The time-dependent CD and UV-vis spectral changes were measured at 20 °C.

1.4 Calculation of thermodynamic parameter: The thermodynamic parameters governing the supramolecular aggregation of *R*-$\mathbf{L^1}$ were obtained by the global fitting of the melting curves. This global fitting is performed by using the equilibrium (EQ) model reported by ten Eikelder and coworkers.[1] The values for the elongation enthalpy (ΔH_e) and the entropy (ΔS_e),

and elongation binding constant (K_e) used in the cooperative supramolecular polymerization models were determined by the global fitting of the heating curves,[2-4] which were obtained by plotting the degree of aggregation (α_{agg}) of R-**L**¹ (7.0 mM) without and with $AgNO_3$ (1.2 equiv.) at 326 nm against temperature with heating experiments. An elongation binding constant (K_e) for aggregation at 293 K was estimated according to equation 1, from which the enthalpy change (ΔH), and the entropy change (ΔS) were determined:

$$K_e = e^{-(\Delta H_e - T\Delta S)/RT} \qquad \text{(equation 1)}$$

1.5 Preparation of silver complexes: Different concentrations (0~2.0 equiv.) of aqueous Ag^+ solution were added to R-$\mathbf{L}^1$ or S-$\mathbf{L}^1$ (6.4 mM) solution in DMSO/H_2O (1:1 v/v). Time- and temperature-dependent CD and UV-vis spectra were measured.

1.6 Calculation of stability constants: The UV-vis titration with $AgNO_3$ for R-$\mathbf{L}^1$ (50 μM) was performed in DMSO/H_2O (1:1 v/v). The titration was performed with 0-3.0 equiv. of $AgNO_3$ at 25 °C. Titration data were fitted into a desired binding model with HyperSpec to calculate stability constants for 1:1 and 2:1 (Ag^+ : ligand) complexes, respectively.[5-7]

1.7 Theoretical calculations: We performed density functional theory (DFT) calculations to optimize the Ag^+ complex systems using the Gaussian 09 package.[8] The unrestricted B3LYP functional was employed for all optimizations and frequency calculations with Def2-SVP level of theory for all atoms.[9-11] All calculations were performed in the gas phase. All the optimized structures were confirmed by vibrational frequency analysis with no imaginary frequency. The Cartesian coordinates are shown in Tables S2-S4.

2. Synthesis and characterization

2.1 Synthesis of R-$\mathbf{L}^2$ and S-$\mathbf{L}^2$

(R or S)-(−)-2-amino-1-propanol (0.28 g, 3.7 mmol) was added to a stirred suspension of powdered KOH (1.05 g, 18.7 mmol) in dry DMSO (20 mL) at 60 °C. After 30 min, 4′-chloro-2,2′:6′,2″-terpyridine (1.00 g, 3.7 mmol) was added to the mixture. The mixture was then stirred for 4 h at 70 °C and poured into 600 mL of distilled water thereafter. CH_2Cl_2 (3 × 200 mL) was used to extract the aqueous phase. Residual water in dichloromethane was dried over Na_2SO_4 and CH_2Cl_2 was removed in vacuum, and the desired product was purified by recrystallization with ethyl acetate to give 0.72 g (72%) of R-$\mathbf{L}^2$ and S-$\mathbf{L}^2$. Mp = 118.3 °C; IR (KBr pellet):

3375, 2964, 2926, 2846, 1577, 1565, 1473, 1439, 1403, 1353, 1204, 799 cm^{-1}; ^{1}H NMR (300 MHz, $CDCl_3$): δ 8.70 (tdd, *J* = 4.8, 1.8, 0.9 Hz, 2H), 8.62 (dt, *J* = 8.0, 1.1 Hz, 2H), 8.02 (s, 2H), 7.84 (td, *J* = 7.7, 1.8 Hz, 2H), 7.33 (ddd, *J* = 7.4, 4.8, 1.2 Hz, 2H), 4.14 (dd, *J* = 9.0, 4.1 Hz, 1H), 3.94 (dd, *J* = 9.1, 7.6 Hz, 1H), 3.41 (dddd, *J* = 10.6, 7.6, 6.6, 4.2 Hz, 1H), 1.21 (d, *J* = 6.5 Hz, 3H); ^{13}C NMR (125 MHz, DMSO-d_6): δ 167.2, 157.1, 155.3, 149.7, 137.9, 125.0, 121.3, 107.3, 75.1, 46.2, 20.43; HR-Mass (m/z) calculated for $C_{18}H_{18}N_4O$ $[M]^+$: 306.3690, Found $[M]^+$: 306.3690.

2.2 Synthesis of *R*-L¹

R-**L¹** and *S*-**L¹** was prepared according to a literature procedure.[12] In a two neck flask, *R*-**L²** (0.50 g, 1.64 mmol) and TEA (0.1 mL, 0.72 mmol) were added to dry CH_2Cl_2 (10 mL). After cooling the solution in an ice bath, sebacoyl chloride (0.16 mL, 0.75 mmol) was added dropwise. The reactant was stirred for 3 h at room temperature. The crude product was recrystallized from CH_2Cl_2 to give a white crystalline solid *R*-**L¹** in 49.7% yield (0.632 g). Mp = 198 °C; IR (KBr pellet): 3428, 3311, 2929, 2845, 1640, 1582, 1563, 1466, 1446, 1407, 1362, 1207, 1038, 785 cm^{-1}; ^{1}H NMR (300 MHz, DMSO-d_6): δ 8.68 (m, 8H), 7.99 (m, 8H), 7.87 (d, *J* = 7.5 Hz, 2H), 7.50 (ddd, *J* = 7.7, 4.8, 1.6 Hz, 4H), 4.15 (m, 6H), 2.02 (t, *J* = 7.3 Hz, 4H), 1.42 (d, *J* = 7.5 Hz, 4H), 1.21 (s, 3H), 1.19 (s, 3H), 1.13 (s, 8H); ^{13}C NMR (125 MHz, DMSO-d_6): δ 172.3, 167.1, 157.2, 155.3, 149.7, 137.8, 125.0, 121.3, 107.2, 70.9, 44.1, 35.9, 29.2, 29.0, 25.7, 17.6; HR-Mass (m/z) calculated for $C_{46}H_{50}N_8O_4$ $[M]^+$: 778.3955, Found $[M]^+$: 778.3954.

2.3 Synthesis of *S*-L¹

The synthesis of *S*-**L¹** was performed as described in the synthesis of *R*-**L¹**. 52.4% yield (0.632 g). Mp = 198 °C; IR (KBr pellet): 3426, 3310, 2928, 2845, 1642, 1581, 1561, 1466, 1445, 1405, 1362, 1206, 1037, 786 cm^{-1}; ^{1}H NMR (300 MHz, DMSO-d_6): δ 8.70 (m, 8H), 7.95 (m, 8H), 7.86 (d, *J* = 7.4 Hz, 2H), 7.46 (ddd, *J* = 7.7, 4.7, 1.6 Hz, 4H), 4.17 (m, 6H), 2.12 (t, *J* = 7.2 Hz, 4H), 1.40 (d, *J* = 7.6 Hz, 4H), 1.19 (s, 3H), 1.17 (s, 3H), 1.11 (s, 8H); ^{13}C NMR (125 MHz, DMSO-d_6): δ 172.1, 167.0, 157.3, 155.0, 149.9, 137.6, 125.2, 121.5, 107.1, 70.8, 44.0, 35.7, 29.0, 28.8, 25.6, 17.5; HR-Mass (m/z) calculated for $C_{46}H_{50}N_8O_4$ $[M]^+$: 778.3955, Found $[M]^+$: 778.3953.

3. Supplementary scheme and figures

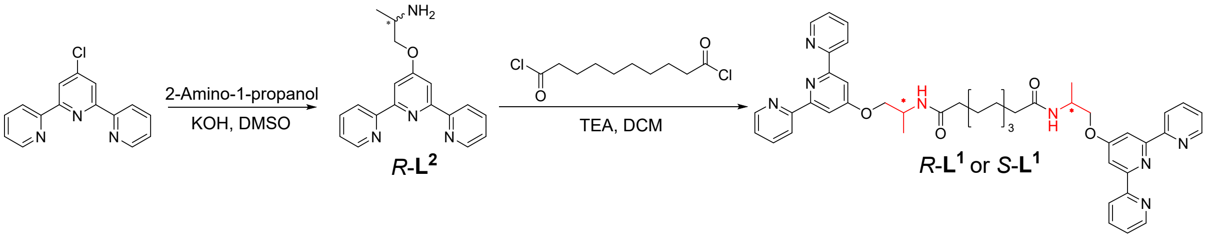

Scheme S1. Synthetic method of *R*-**L**[1] or *S*-**L**[1].

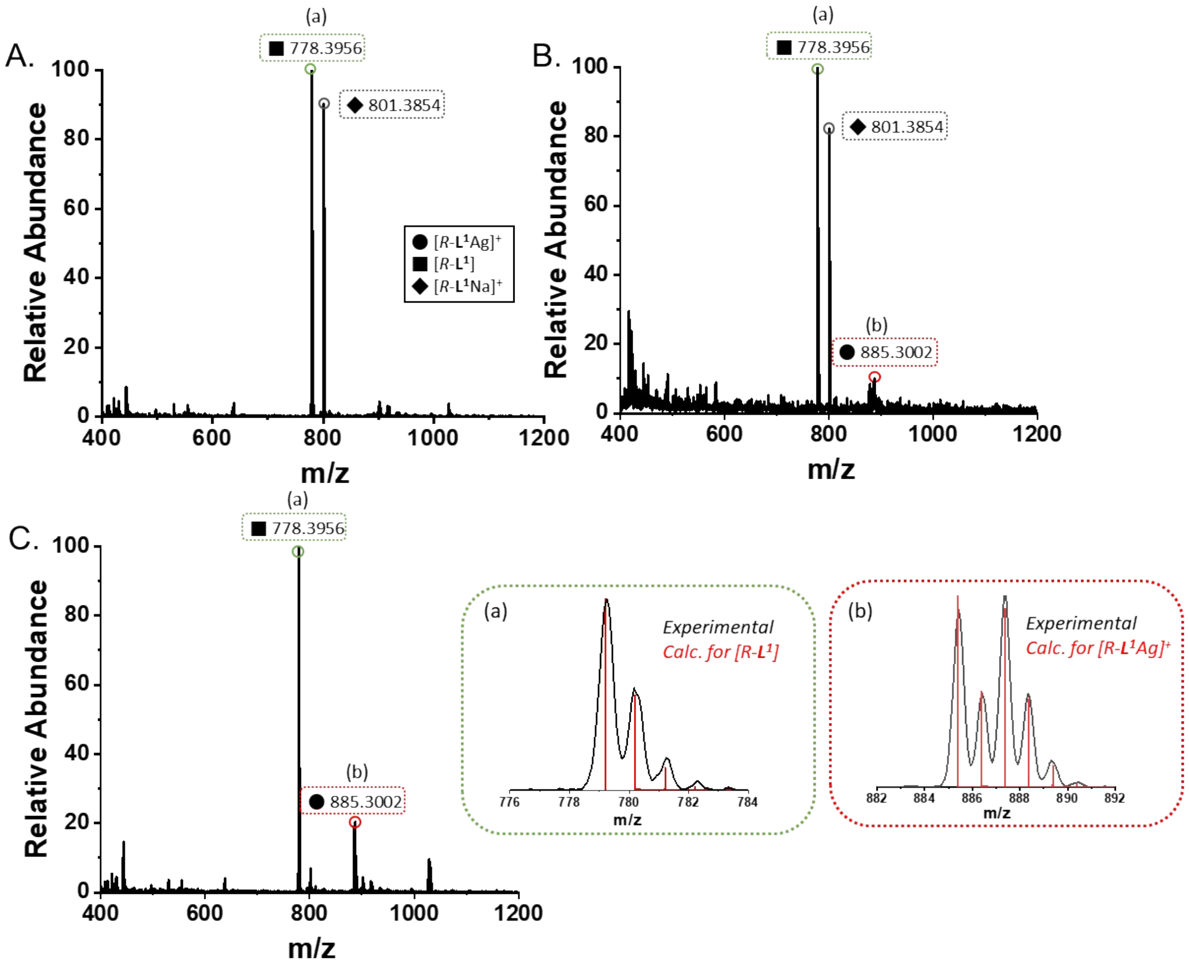

Fig. S1 HR-ESI-MS spectra of *R*-**L**[1] (6.4 mM) in the presence of different equiv. of $AgNO_3$; (A) 0 equiv., (B) 0.1 equiv. and (C) 0.2 equiv. in DMSO/H_2O (1:1 v/v) after 72 h aging.

[*Note*] The peaks at m/z 801.3854 correspond to [*R*-**L**[1]+Na]$^+$.

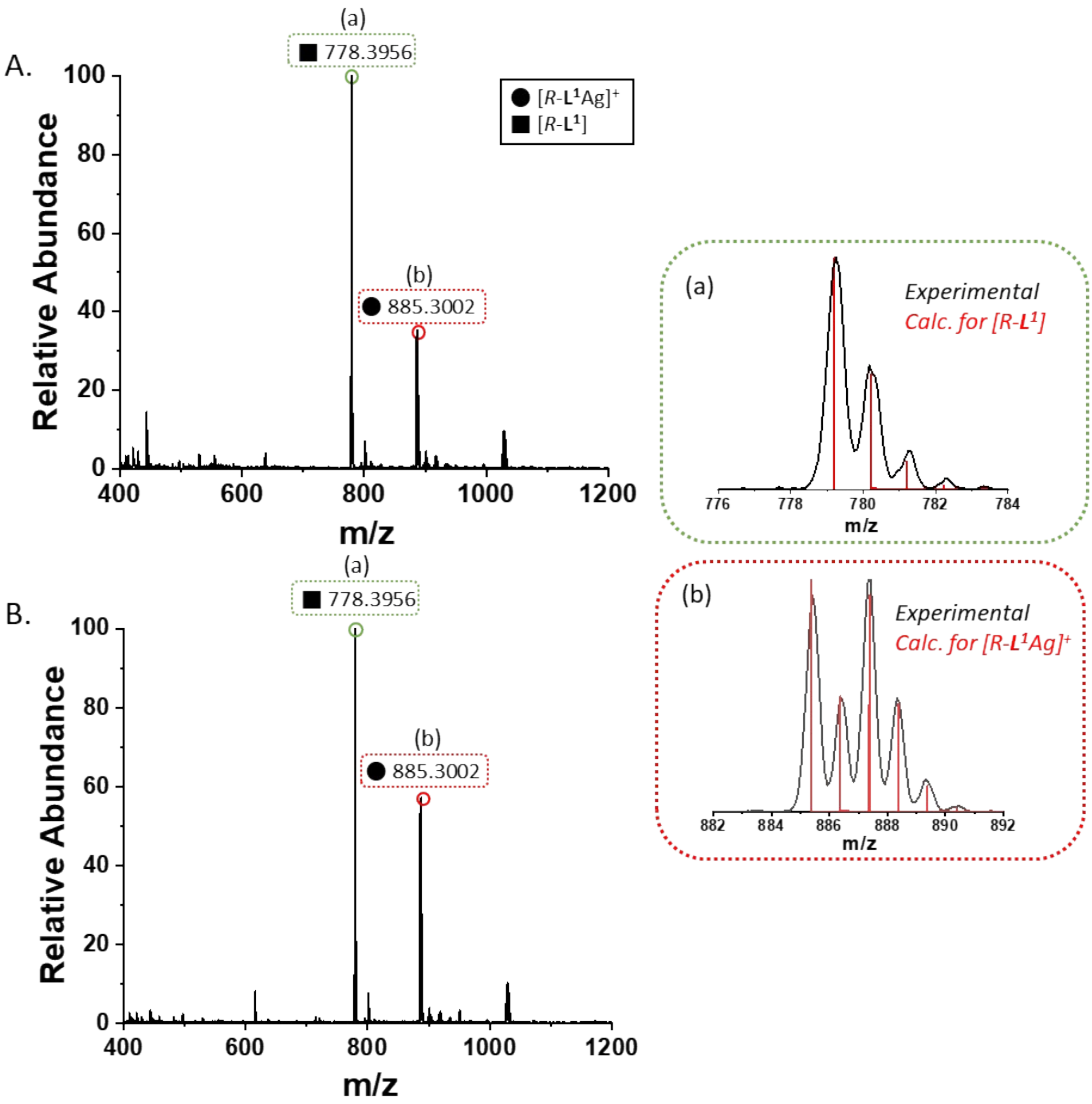

Fig. S2 HR-ESI-MS spectra of *R*-**L**^{1} (6.4 mM) in the presence of different equiv. of $AgNO_3$: (A) 0.3 equiv. and (B) 0.4 equiv. in DMSO/H_2O (1:1 v/v) after 72 h aging.

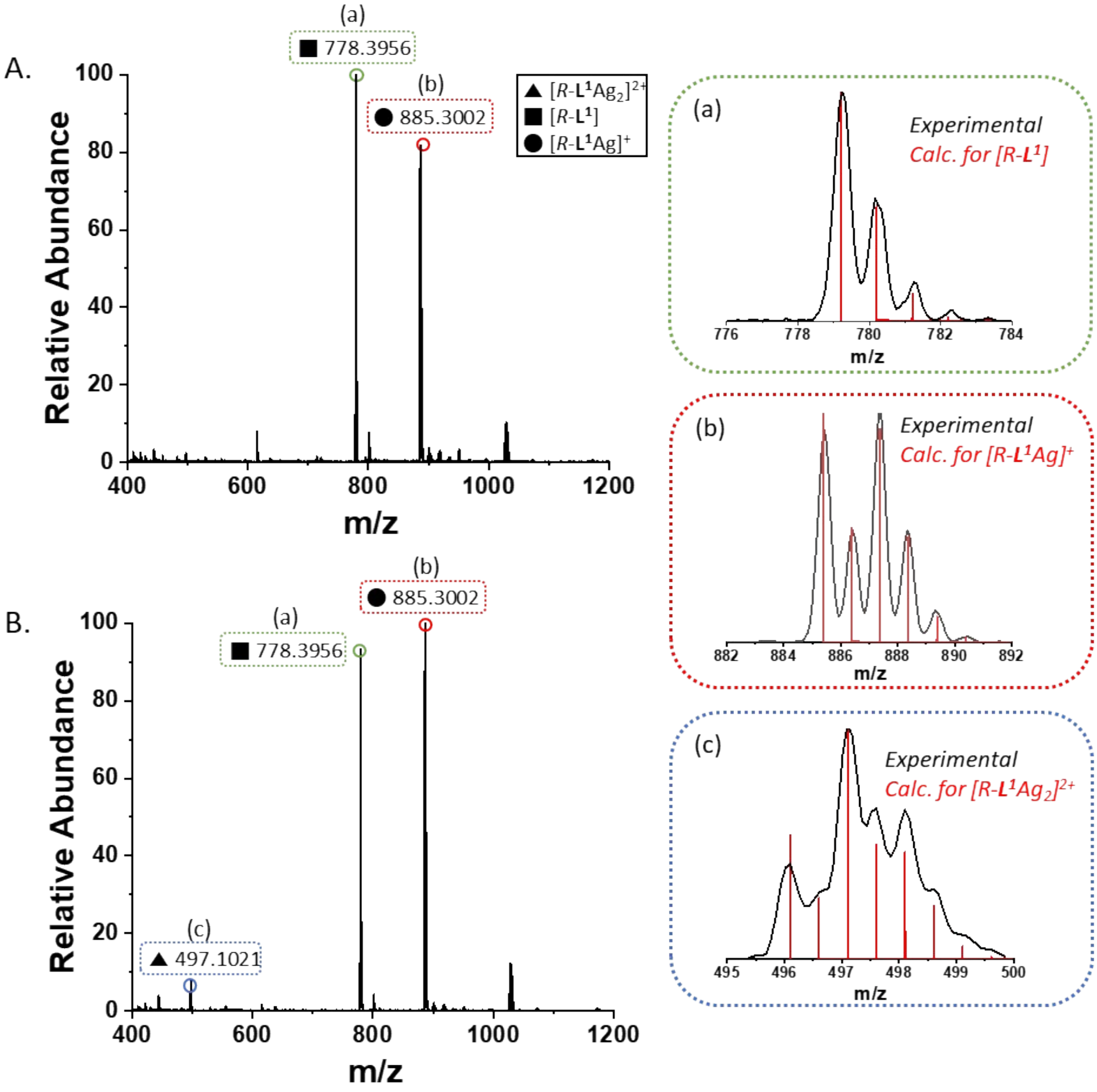

Fig. S3 HR-ESI-MS spectra of *R*-**L**¹ (6.4 mM) in the presence of different equiv. of $AgNO_3$; (A) 0.5 equiv. and (B) 0.6 equiv. in DMSO/H_2O (1:1 v/v) after 72 h aging.

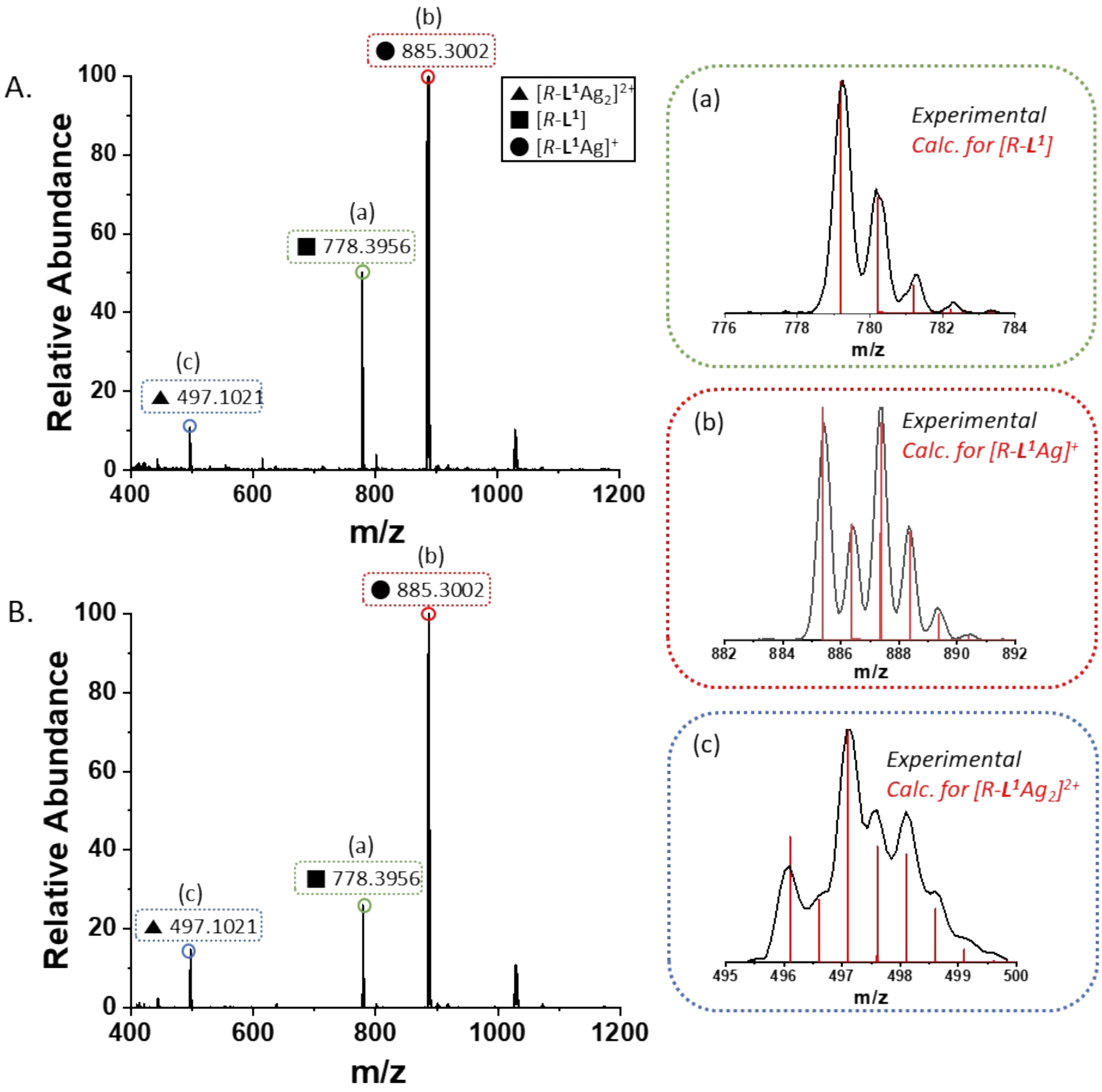

Fig. S4 HR-ESI-MS spectra of *R*-**L**[1] (6.4 mM) in the presence of different equiv. of $AgNO_3$; (A) 0.8 equiv. and (B) 1.0 equiv. in DMSO/H_2O (1:1 v/v) after 72 h aging.

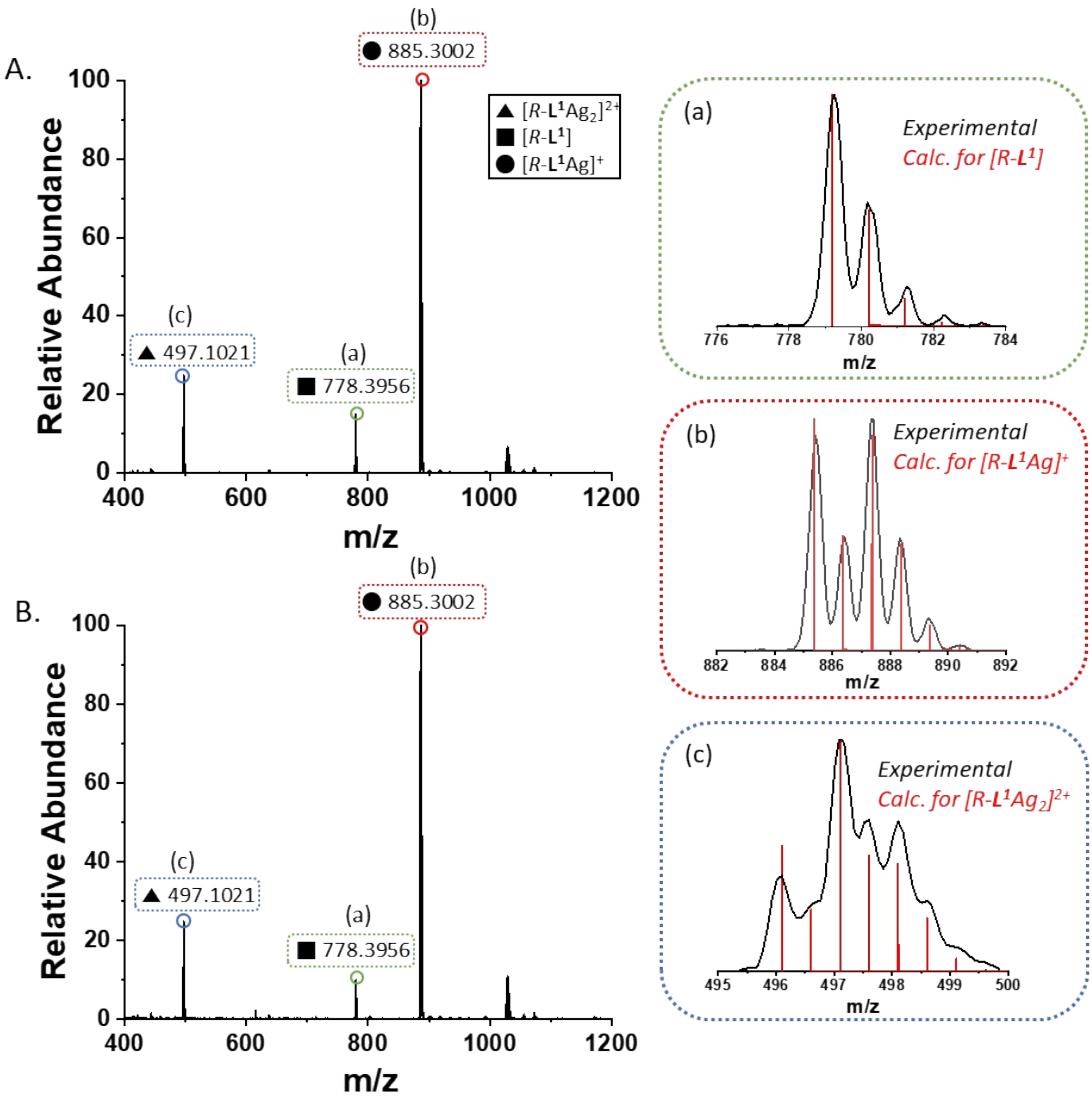

Fig. S5 HR-ESI-MS spectra of *R*-**L**[1] (6.4 mM) in the presence of different equiv. of $AgNO_3$; (A) 1.2 equiv. and (B) 1.4 equiv. in DMSO/H_2O (1:1 v/v) after 72 h aging.

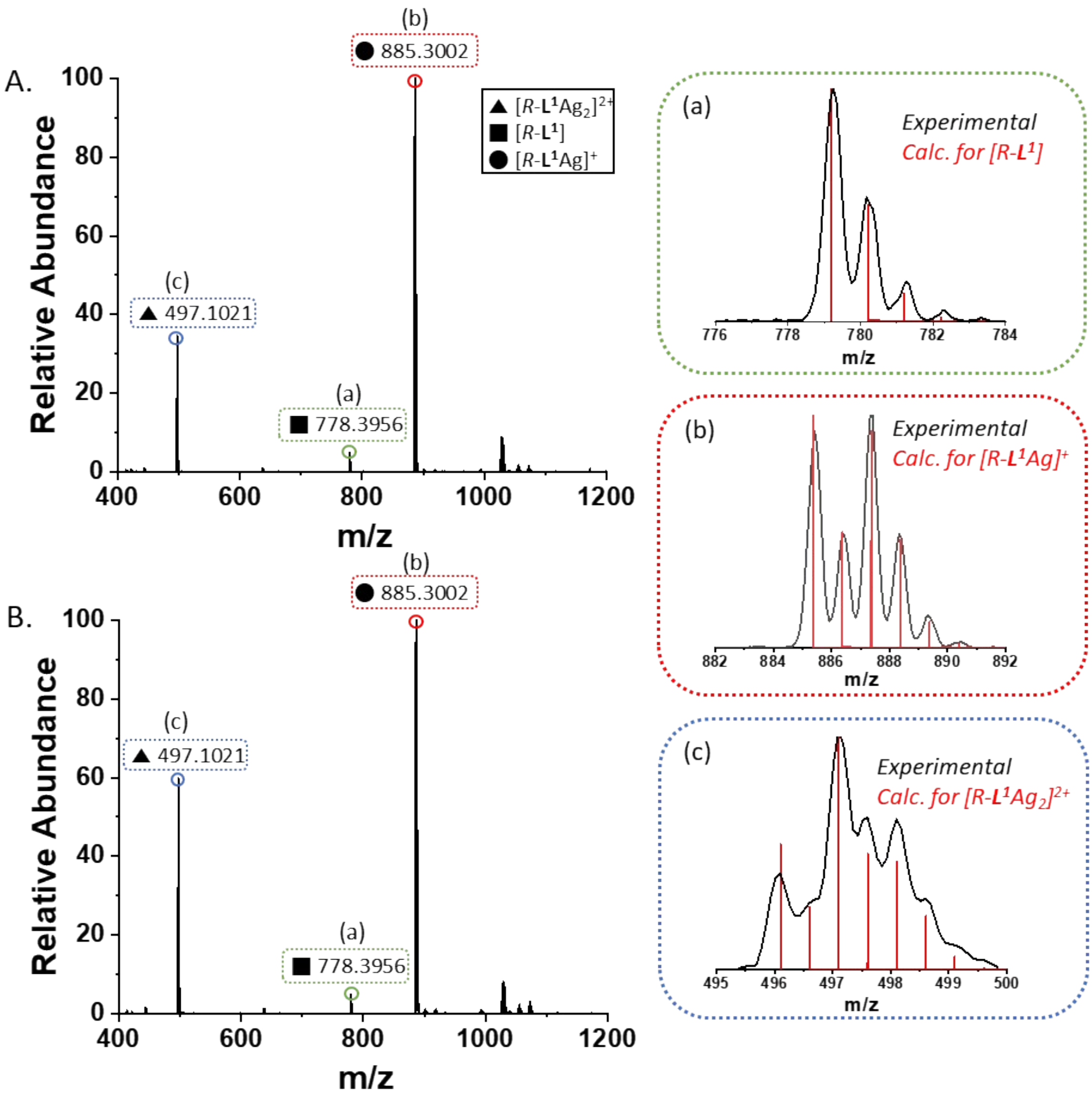

Fig. S6 HR-ESI-MS spectra of *R*-**L**[1] (6.4 mM) in the presence of different equiv. of $AgNO_3$; (A) 1.6 equiv., and (B) 1.8 equiv. in DMSO/H_2O (1:1 v/v) after 72 h aging.

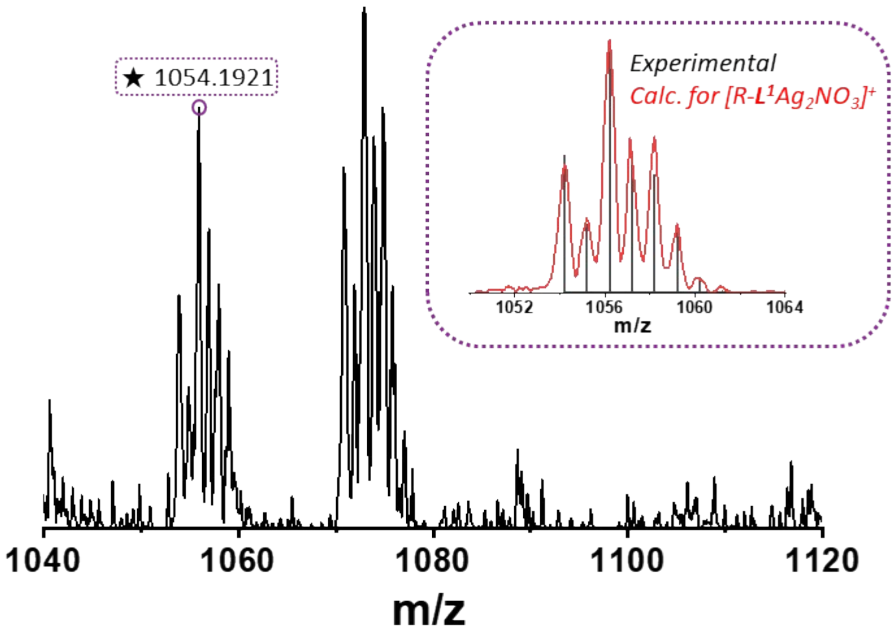

Fig. S7 HR-ESI-MS spectrum of [R-**L**^{1}Ag$_2$NO$_3$]$^+$.

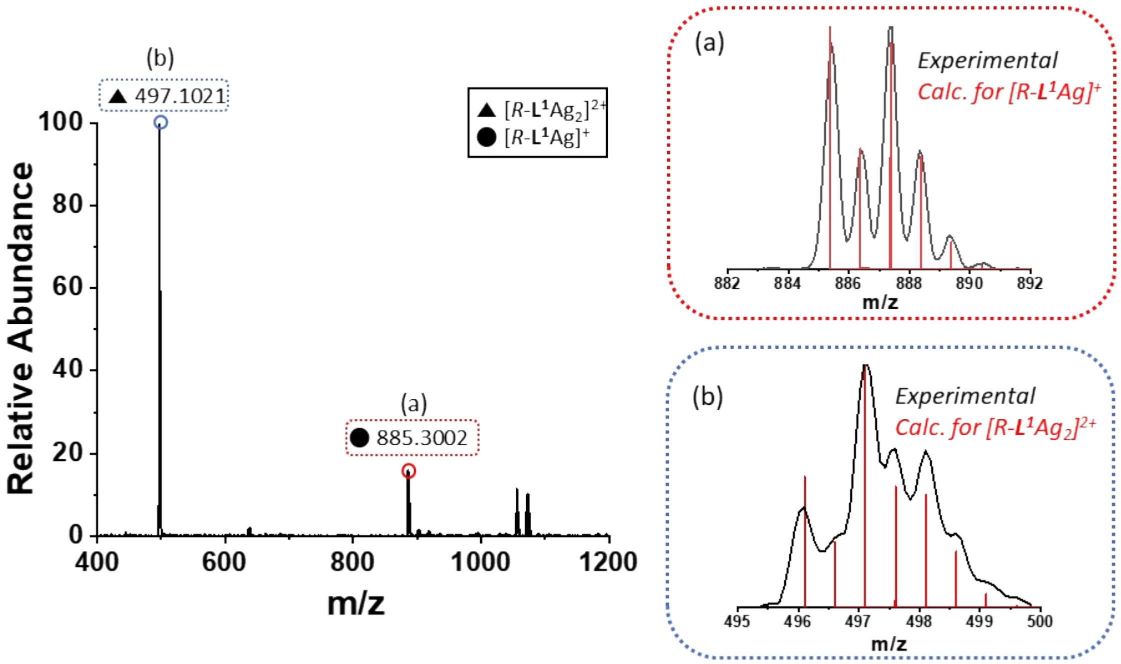

Fig. S8 HR-ESI-MS spectrum of R-**L**^{1} (6.4 mM) in the presence of AgNO$_3$ (2.0 equiv.) in DMSO/H$_2$O (1:1 v/v) after 72 h aging.

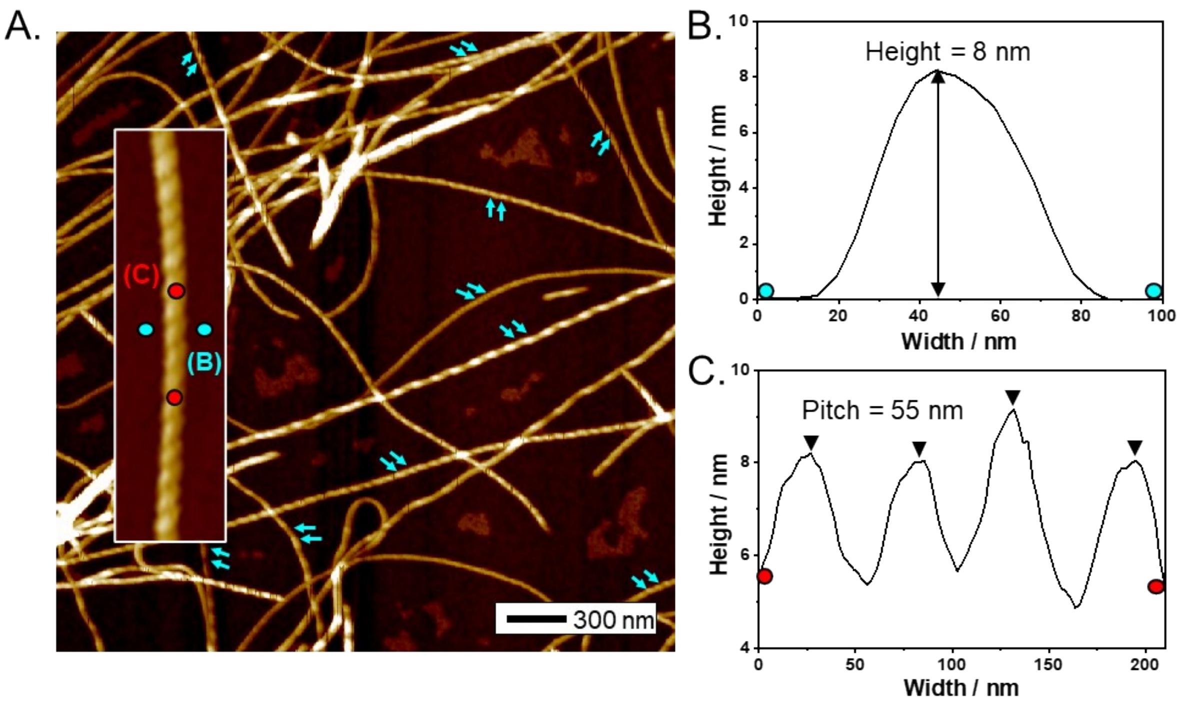

Fig. S9 (A) AFM image of *R*-**L**[1] (6.4 mM) without $AgNO_3$ in DMSO/H_2O (1:1 v/v) after 72 h aging. Cross-sectional analysis (B) blue dots (for height) and (C) red dots (for pitch) in the image.

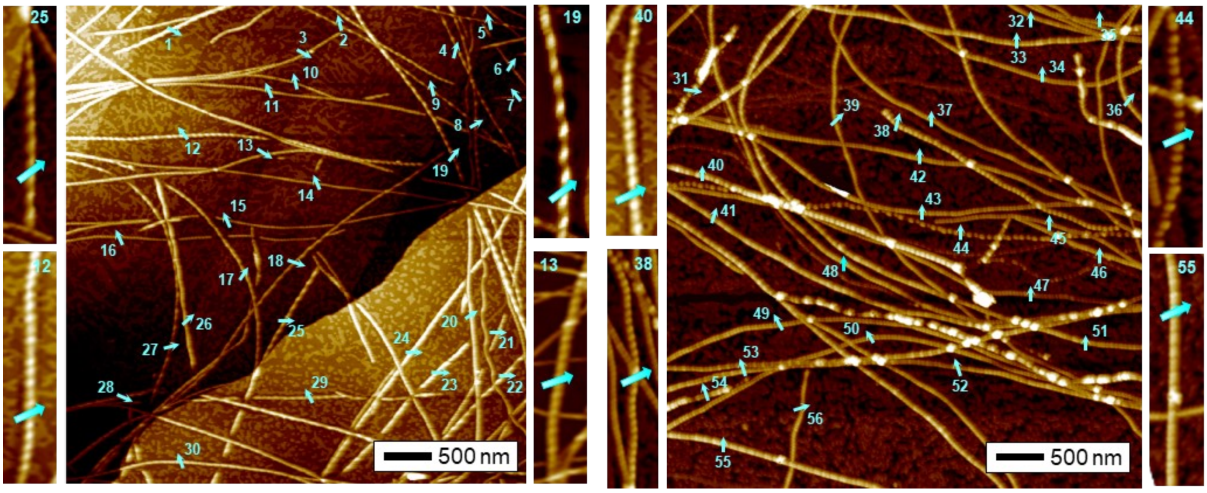

Fig. S10 AFM images of *R*-**L**[1] (6.4 mM) in DMSO/H_2O (1:1 v/v) after 72 h aging. No shown all AFM images.

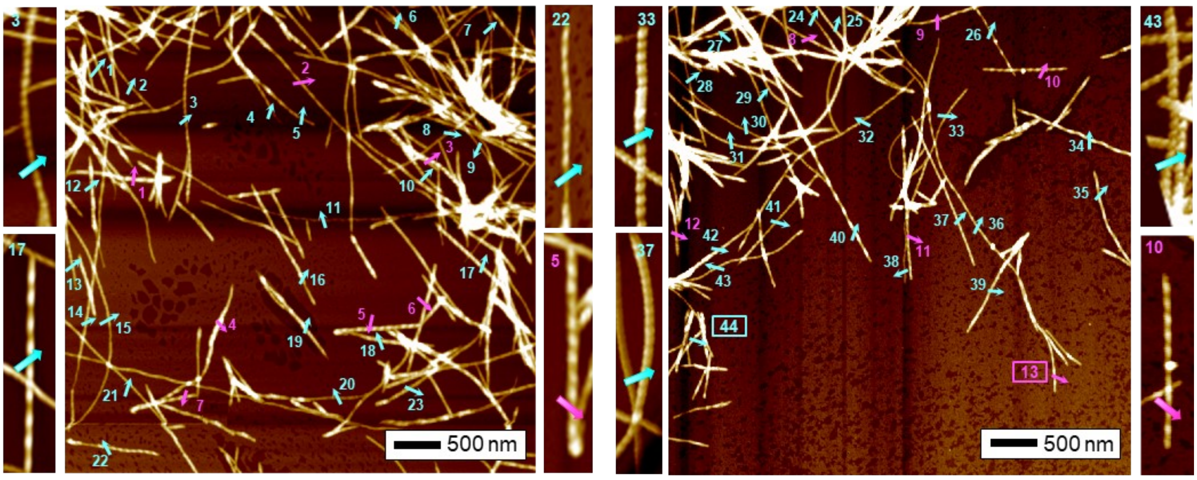

Fig. S11 AFM images of *R*-**L**[1] (6.4 mM) with 0.2 equiv. of $AgNO_3$ in DMSO/H_2O (1:1 v/v) after 72 h aging. The blue arrows indicate right-handed helical fiber. The pink arrows indicate left-handed helical fiber. No shown all AFM images.

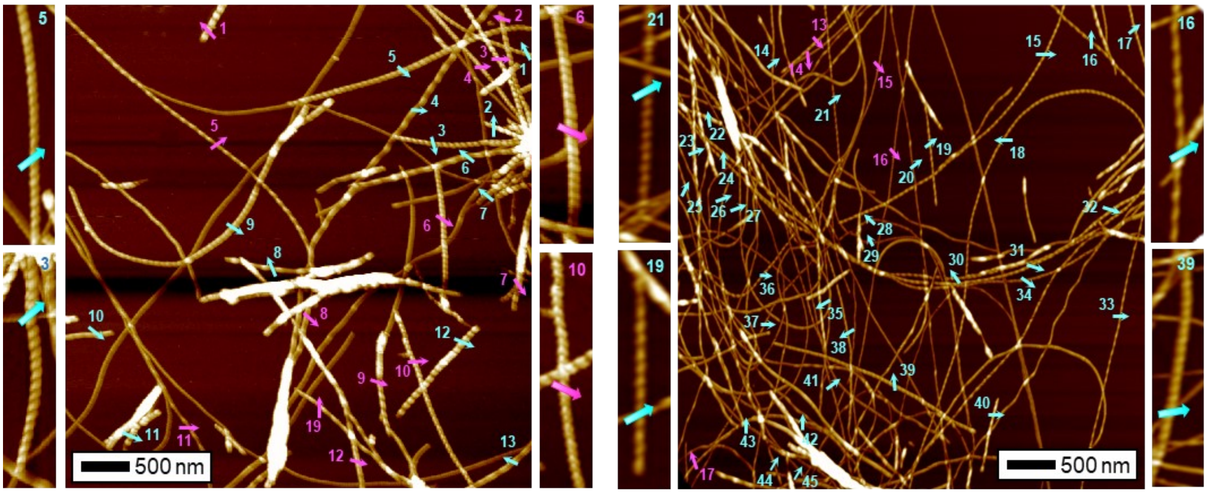

Fig. S12 AFM images of *R*-**L**[1] (6.4 mM) with 0.4 equiv. of $AgNO_3$ in DMSO/H_2O (1:1 v/v) after 72 h aging. No Shown all AFM images. The blue arrows indicate right-handed helical fiber. The pink arrows indicate left-handed helical fiber.

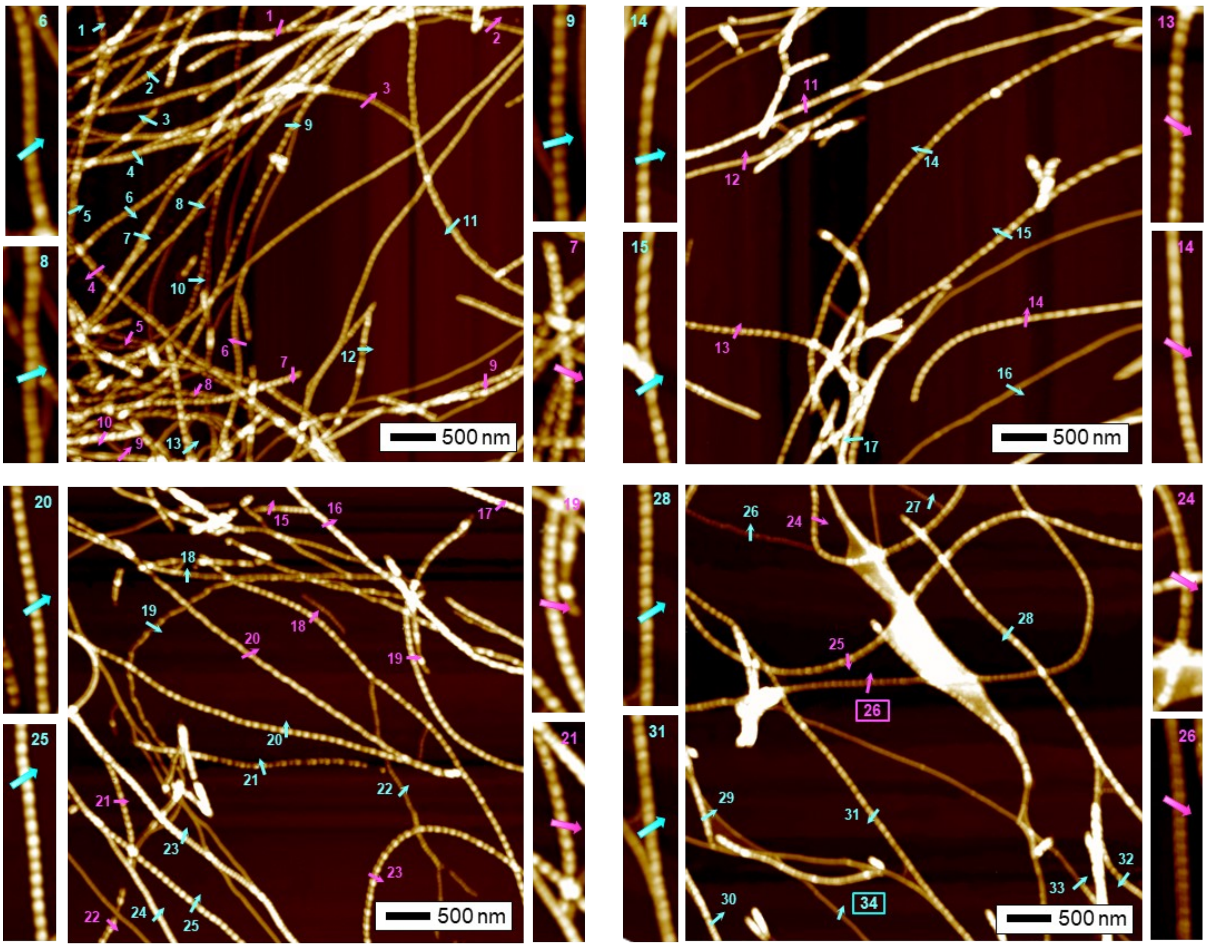

Fig. S13 AFM images of *R*-**L**^{1} (6.4 mM) with 0.6 equiv. of $AgNO_3$ in DMSO/H_2O (1:1 v/v) after 72 h aging. No shown all AFM images. The blue arrows indicate right-handed helical fi ber. The pink arrows indicate left-handed helical fiber.

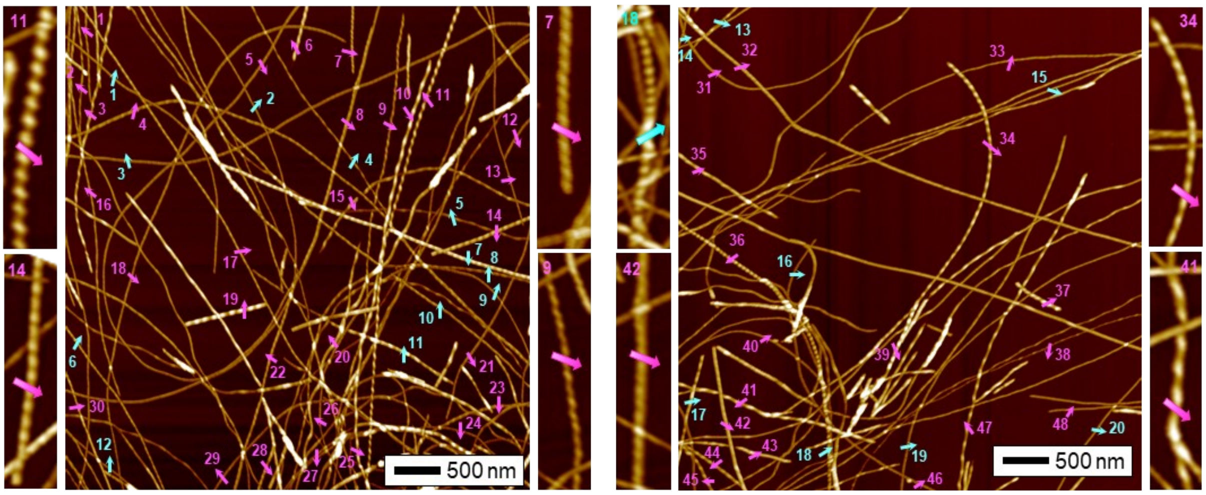

Fig. S14 AFM images of *R*-$\mathbf{L}^1$ (6.4 mM) with 0.8 equiv. of $AgNO_3$ in DMSO/H_2O (1:1 v/v) after 72 h aging. No shown all AFM images. The blue arrows indicate right-handed helical fi ber. The pink arrows indicate left-handed helical fiber.

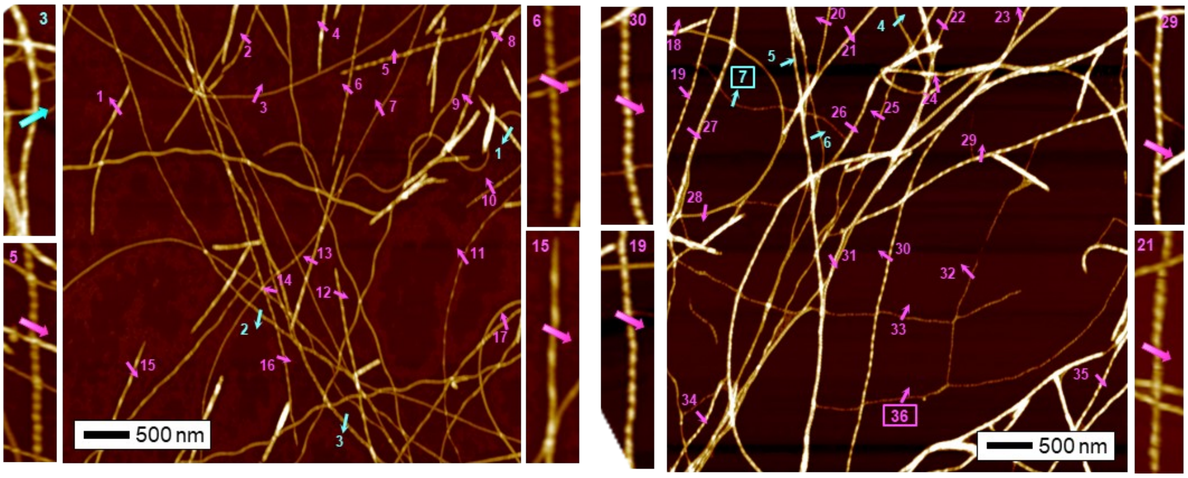

Fig. S15 AFM images of *R*-$\mathbf{L}^1$ (6.4 mM) with 1.0 equiv. of $AgNO_3$ in DMSO/H_2O (1:1 v/v) after 72 h aging. No shown all AFM images. The blue arrows indicate right-handed helical fiber. The pink arrows indicate left-handed helical fiber.

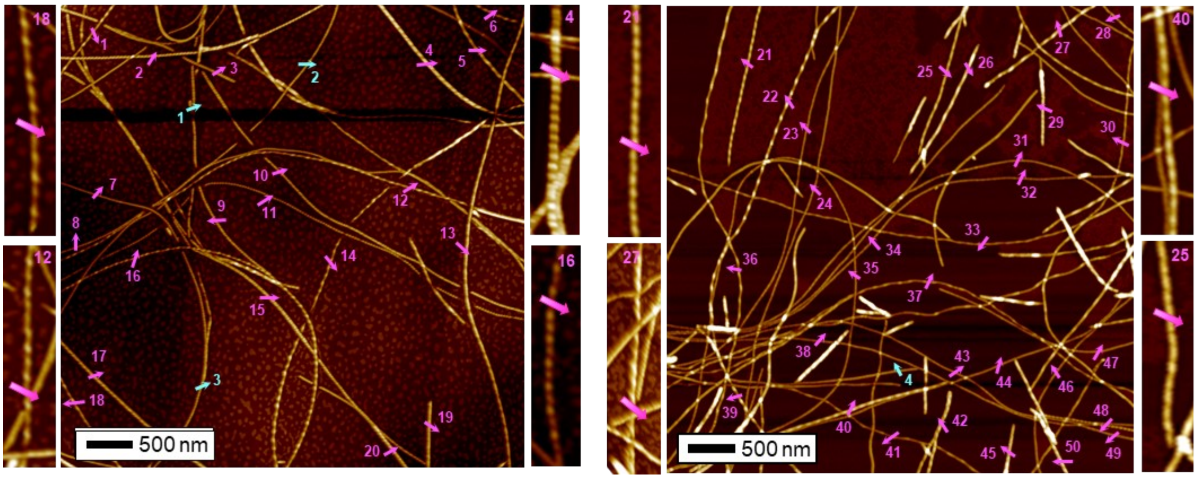

Fig. S16 AFM images of *R*-**L**[1] (6.4 mM) with 1.2 equiv. of $AgNO_3$ in DMSO/H_2O (1:1 v/v) after 72 h aging. No shown all AFM images. The blue arrows indicate right-handed helical fiber. The pink arrows indicate left-handed helical fiber.

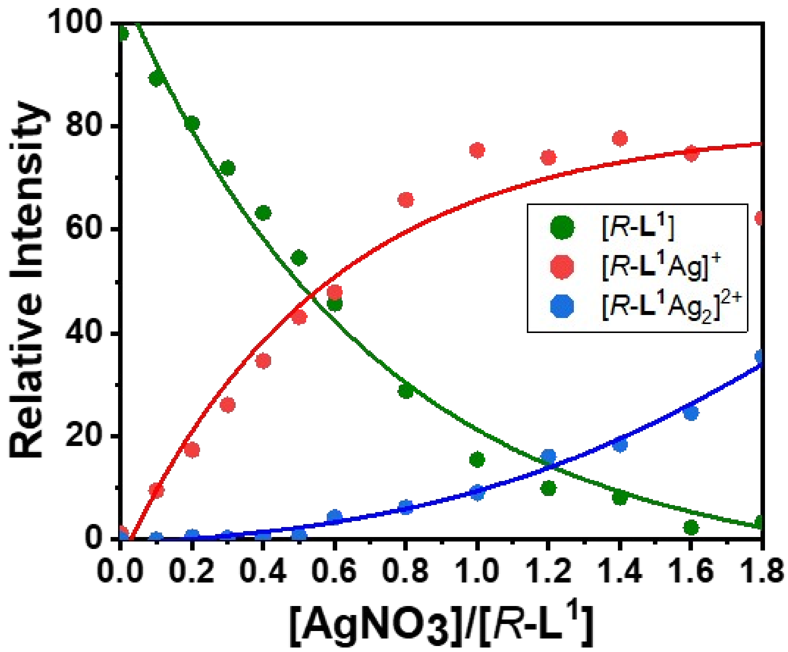

Fig. S17 Species distribution depending on $[AgNO_3]/[R\text{-}\mathbf{L}^1]$ ratios monitored by ESI-MS.

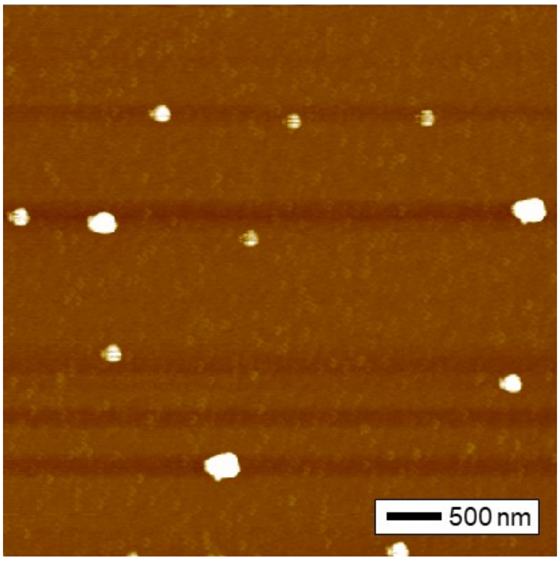

Fig. S18 AFM images of *R*-**L**^{1} (6.4 mM) with 2.0 equiv. of $AgNO_3$ in DMSO/H_2O (1:1 v/v) after 72 h aging.

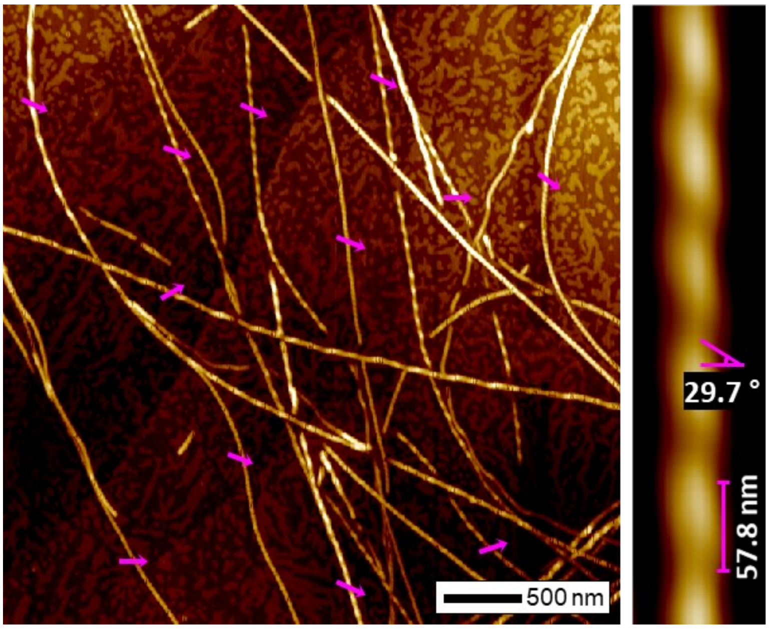

Fig. S19 AFM image of *S*-**L**^{1} (6.4 mM) without $AgNO_3$ in DMSO/H_2O (1:1 v/v) after 72 h aging.

[*Note*] Since unaggregated species are visible in the AFM background, we did not include the background in the calculation of right- and left-handed fibers.

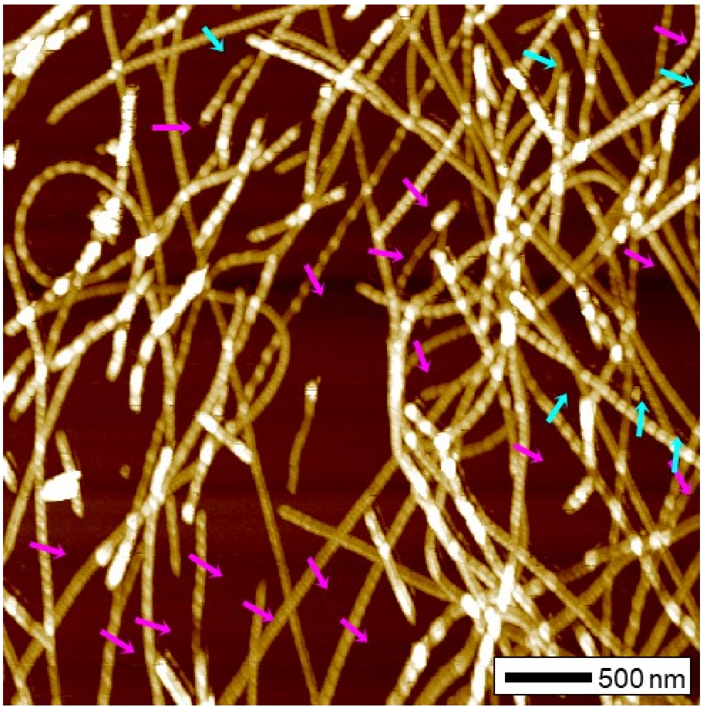

Fig. S20 AFM image of *S*-$\mathbf{L}^1$ (6.4 mM) with 0.2 equiv. of $AgNO_3$ in DMSO/H_2O (1:1 v/v) a fter 72 h aging. The blue arrows indicate right-handed helical fiber. The pink arrows indicate left-handed helical fiber.

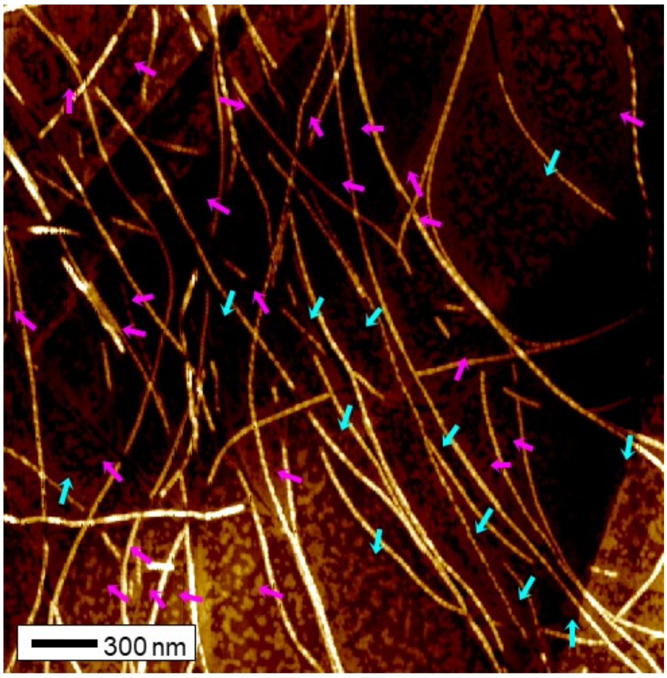

Fig. S21 AFM image of *S*-$\mathbf{L}^1$ (6.4 mM) with 0.4 equiv. of $AgNO_3$ in DMSO/H_2O (1:1 v/v) after 72 h aging. The blue arrows indicate right-handed helical fiber. The pink arrows indicate left-handed helical fiber.

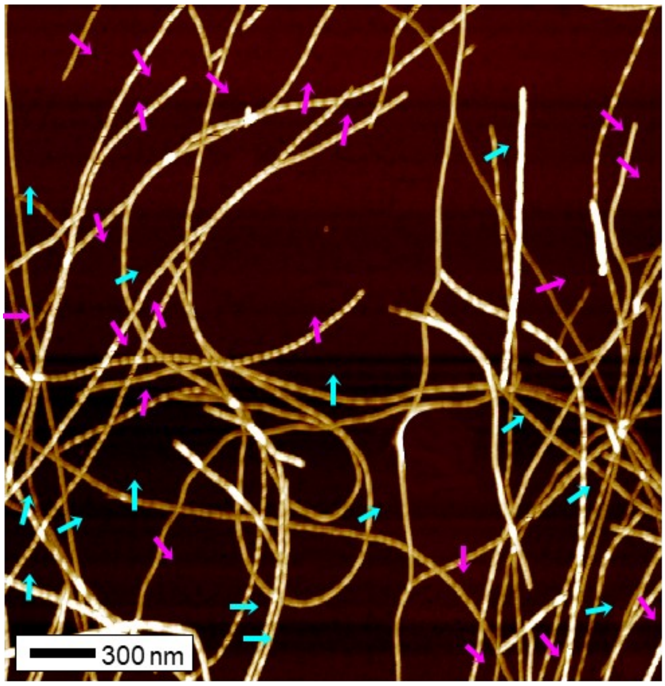

Fig. S22 AFM image of *S*-**L**[1] (6.4 mM) with 0.6 equiv. of $AgNO_3$ in DMSO/H_2O (1:1 v/v) after 72 h aging. The blue arrows indicate right-handed helical fiber. The pink arrows indicate left-handed helical fiber.

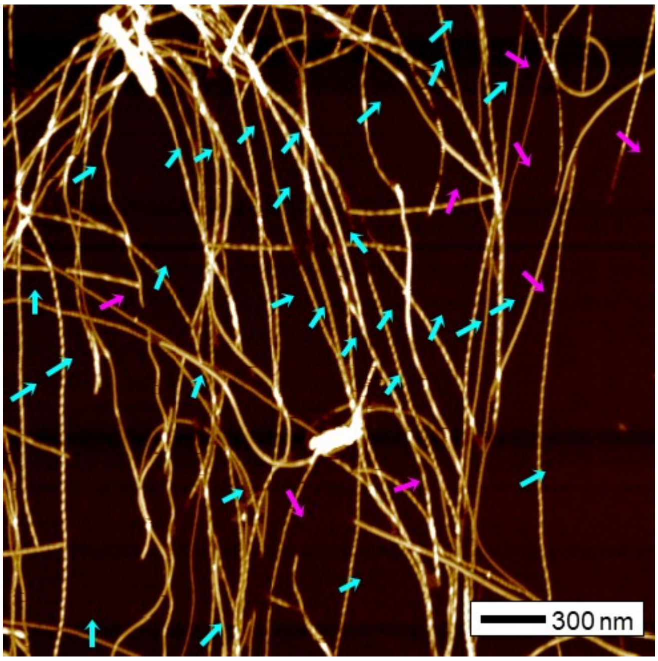

Fig. S23 AFM image of *S*-**L**[1] (6.4 mM) with 0.8 equiv. of $AgNO_3$ in DMSO/H_2O (1:1 v/v) after 72 h aging. The blue arrows indicate right-handed helical fiber. The pink arrows indicate left-handed helical fiber.

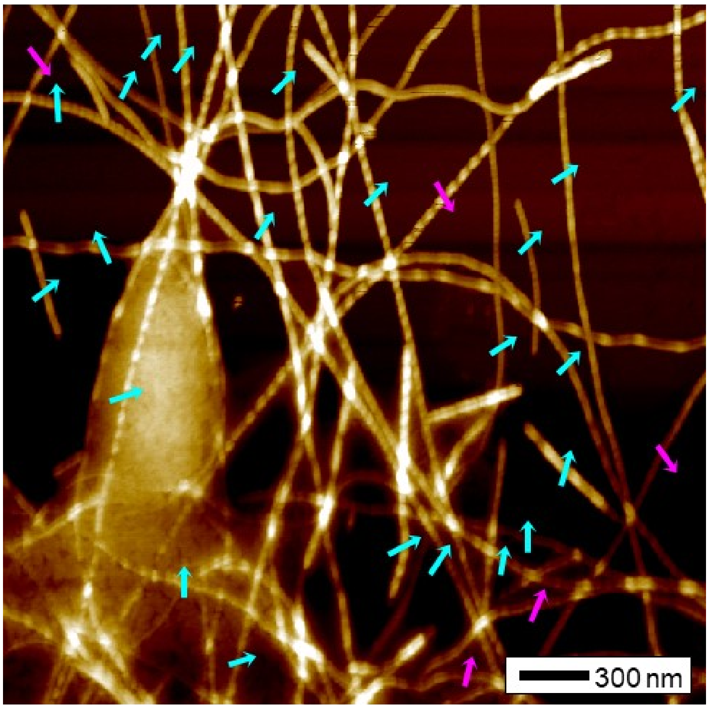

Fig. S24 AFM image of *S*-**L**[1] (6.4 mM) with 1.0 equiv. of $AgNO_3$ in DMSO/H_2O (1:1 v/v) after 72 h aging. The blue arrows indicate right-handed helical fiber. The pink arrows indicate left-handed helical fiber.

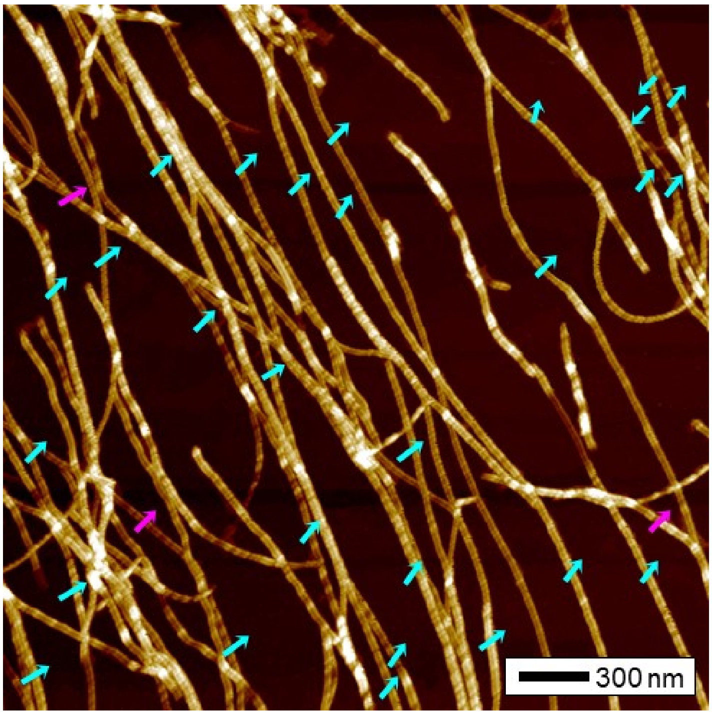

Fig. S25 AFM image of *S*-**L**[1] (6.4 mM) with 1.2 equiv. of $AgNO_3$ in DMSO/H_2O (1:1 v/v) a fter 72 h aging. The blue arrows indicate right-handed helical fiber. The pink arrows indicate left-handed helical fiber.

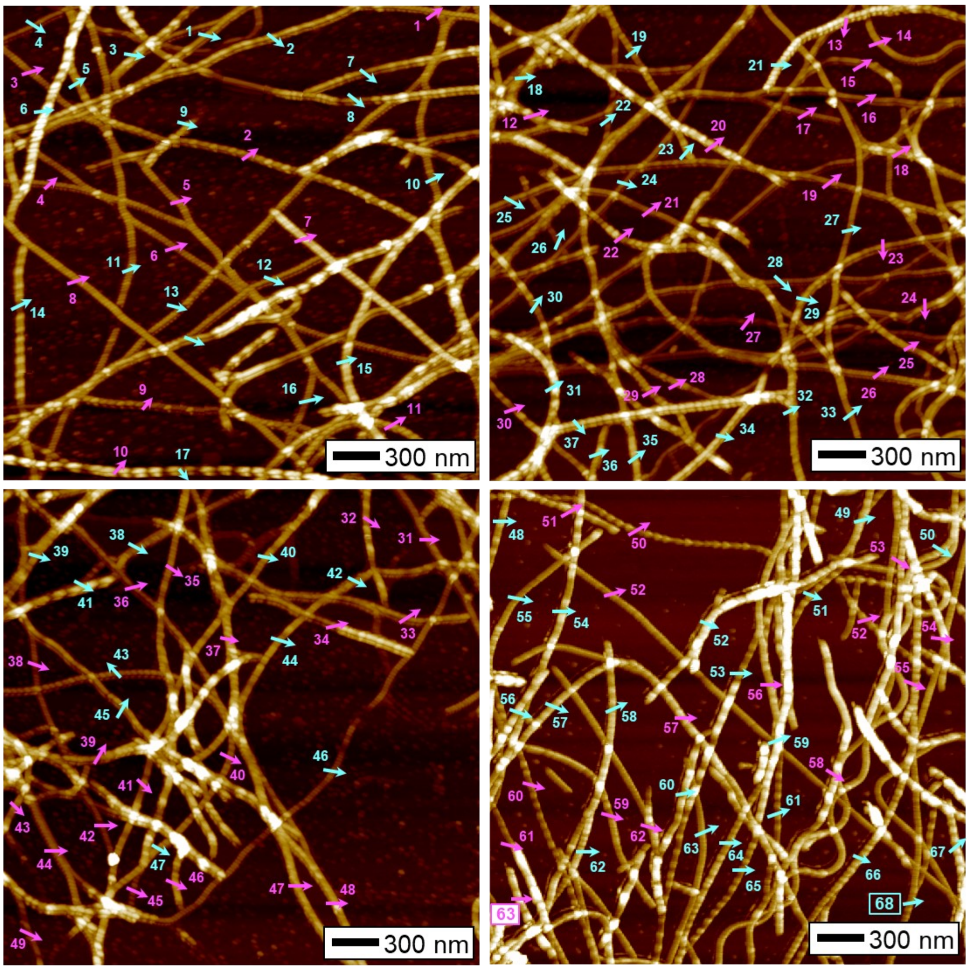

Fig. S26 AFM images of a mixure of *R*-$\mathbf{L}^1$ (6.4 mM) and *S*-$\mathbf{L}^1$ (6.4 mM) without $AgNO_3$ in DMSO/H_2O (1:1 v/v) after 72 h aging. The blue arrows indicate right-handed helical fiber. The pink arrows indicate left-handed helical fiber.

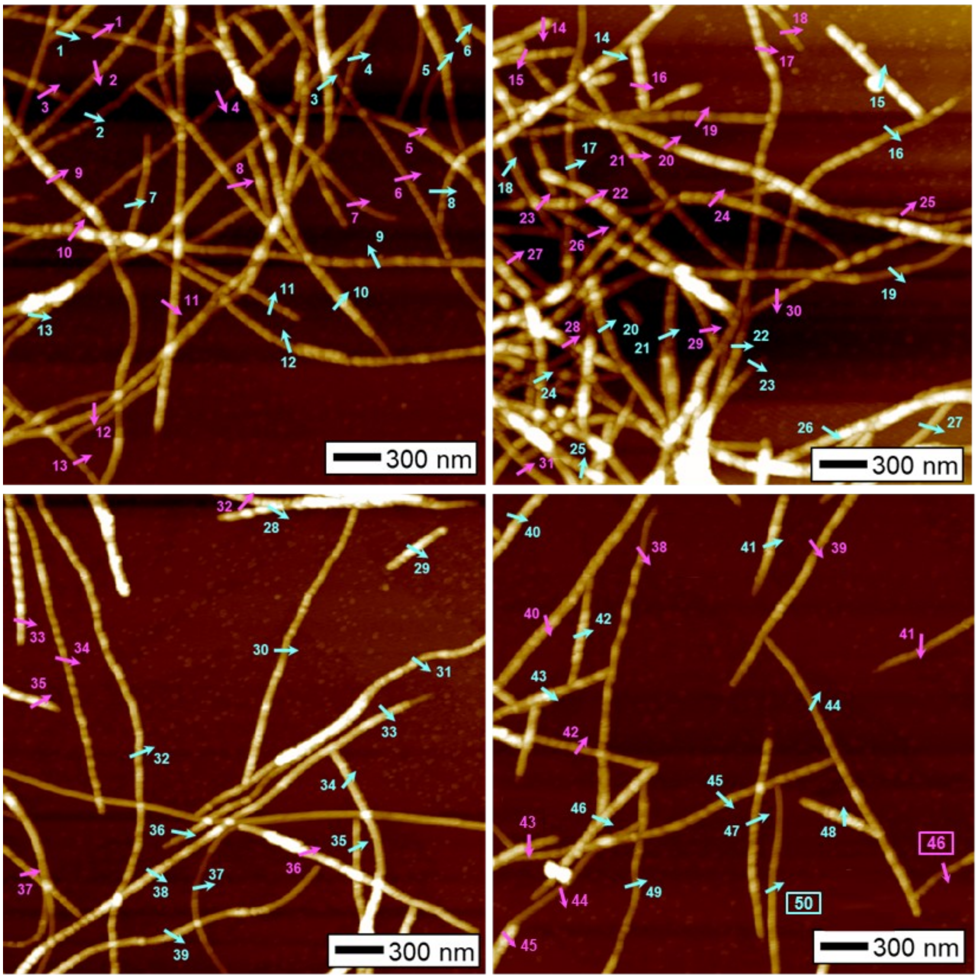

Fig. S27 AFM image of a mixure of *R*-**L**[1] (6.4 mM) and *S*-**L**[1] (6.4 mM) with 0.6 equiv. of $AgNO_3$ in DMSO/H_2O (1:1 v/v) after 72 h aging. The blue arrows indicate right-handed helical fiber. The pink arrows indicate left-handed helical fiber.

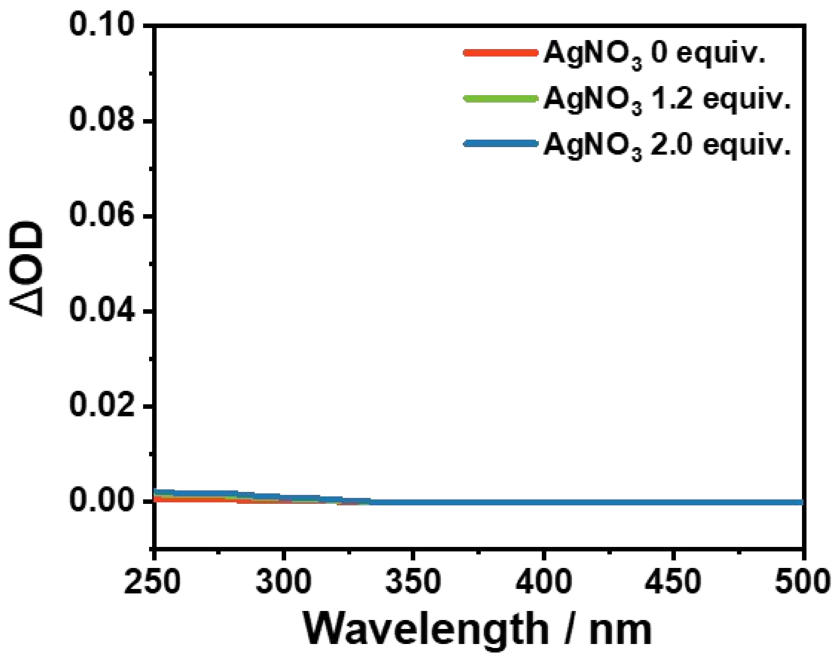

Fig. S28 LD spectra of *R*-**L**[1] (6.4 mM) with $AgNO_3$ (0, 1.2, and 2.0 equiv.) in DMSO/H_2O (1: 1 v/v) after 72 h aging.

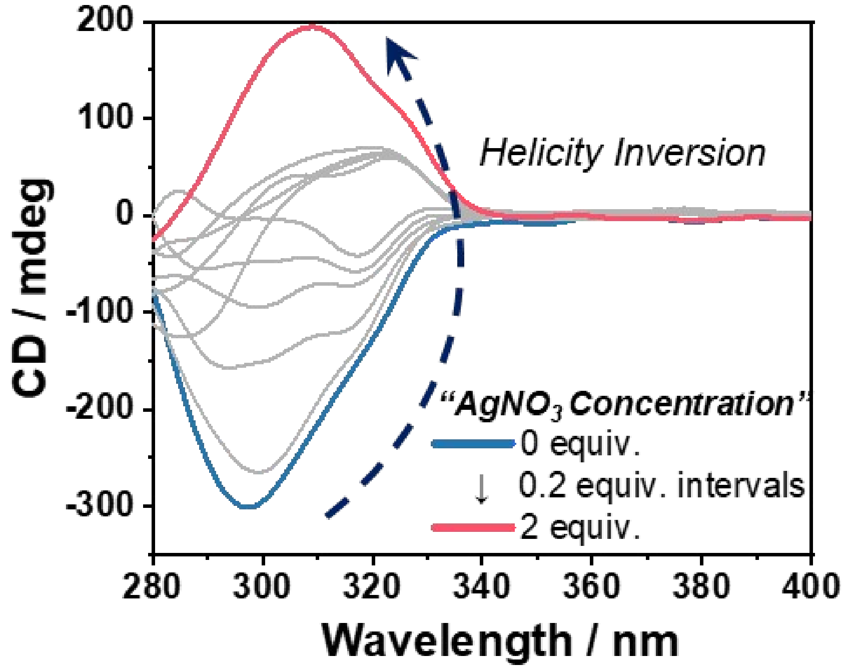

Fig. S29 CD spectra of *S*-**L**[1] (8 mM) upon stepwise addition of $AgNO_3$ (0-2.0 equiv.) in DMSO/H_2O (1:1 v/v). The negative signal observed at ~297 nm (blue line) originates from free *S*-**L**[1], while the positive signal at ~307 nm (red line) originates from [*S*-**L**^{1}Ag]$^+$.

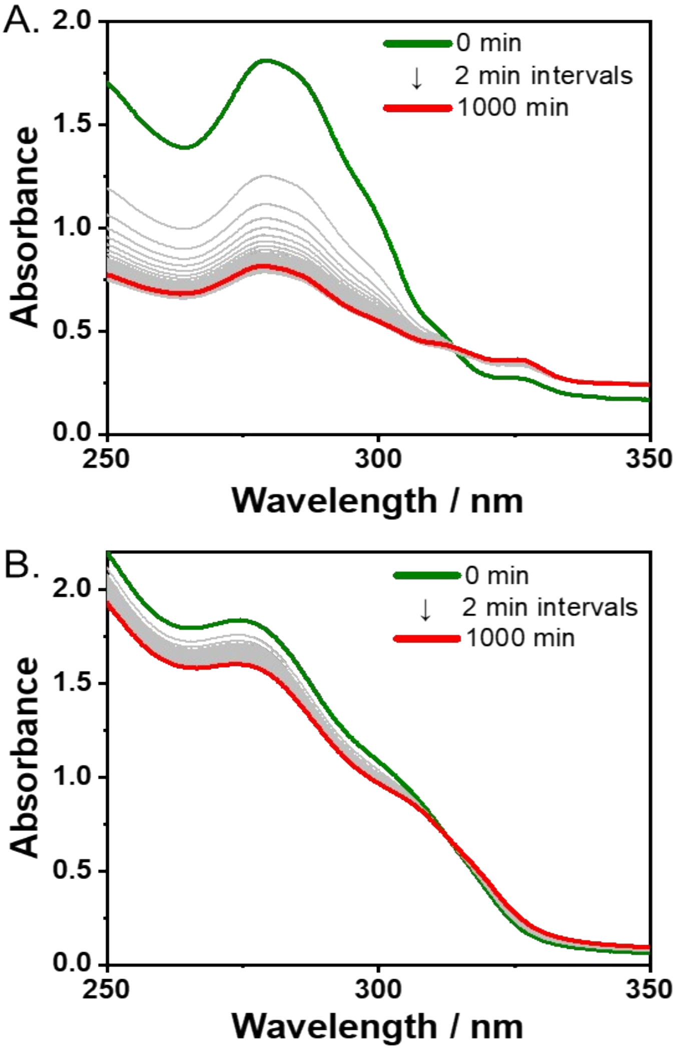

Fig. S30 Time-dependent (0-1000 min) UV-vis spectra of *R*-$\mathbf{L}^1$ (7.2 mM) in the presence of different equiv. of $AgNO_3$ in DMSO/H_2O (1:1 v/v) (cell path length: 0.2 mm): (a) 0 equiv. and (b) 0.4 equiv.

[*Note*] To elucidate the supramolecular polymerization process, we first prepared the sample by dissolving *R*-$\mathbf{L}^1$ (7.2 mM) in H_2O/DMSO (1:1 v/v). After adding the sample to the UV cell, it was heated to 90 °C (1 °C/min) to form the monomeric species in UV-vis spectroscopy. Then the sample was cooled to 20 °C (5 °C/min) in UV-vis spectroscopy. The time-dependent UV-vis spectral changes were measured at 20 °C.

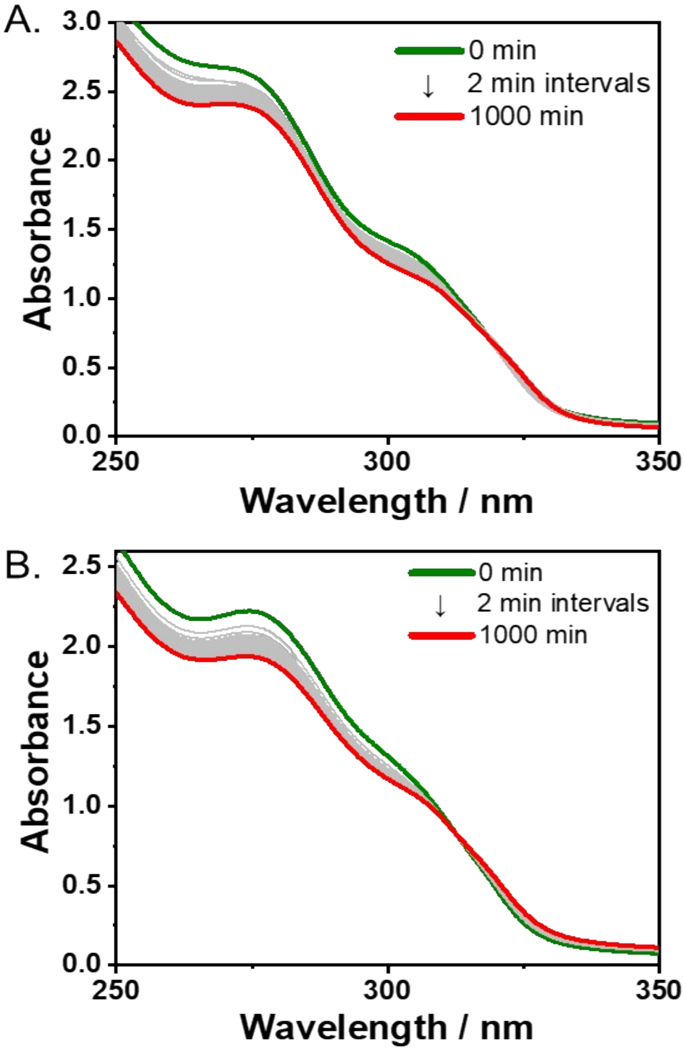

Fig. S31 Time-dependent (0-1000 min) UV-vis spectra of *R*-$\mathbf{L^1}$ (7.2 mM) in the presence of different equiv. of $AgNO_3$ in DMSO/H_2O (1:1 v/v) (cell path length: 0.2 mm): (a) 0.6 equiv. and (b) 0.8 equiv.

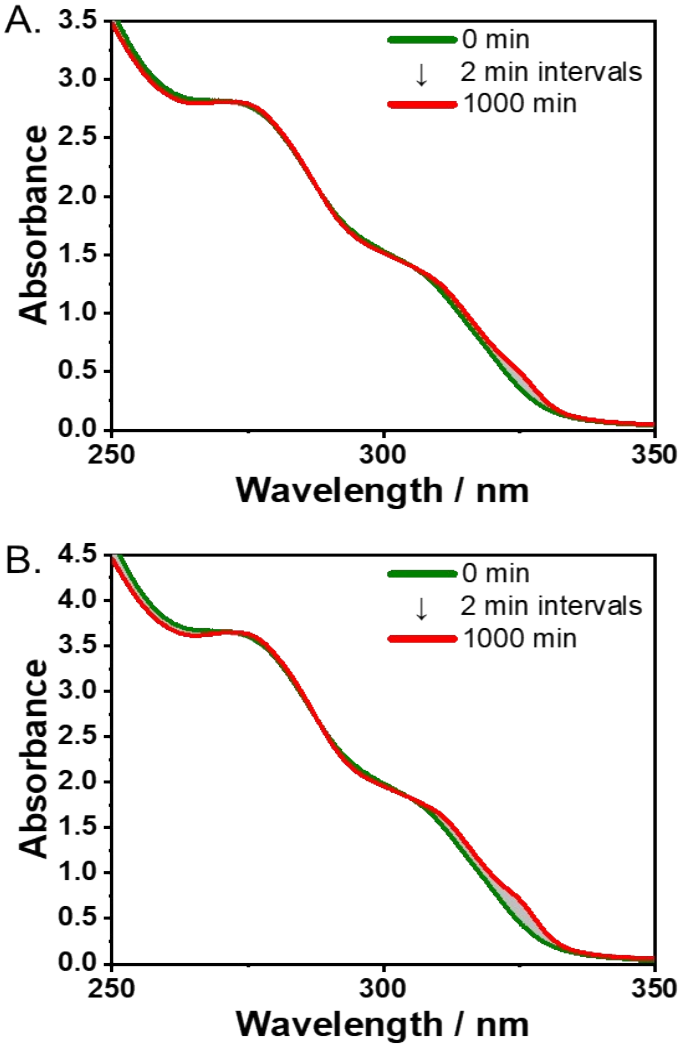

Fig. S32 Time-dependent (0-1000 nm) UV-vis spectra of *R*-**L**[1] (7.2 mM) in the presence of different equiv. of $AgNO_3$ in DMSO/H_2O (1:1 v/v) (cell path length: 0.2 mm): (a) 1.2 equiv. and (b) 1.4 equiv.

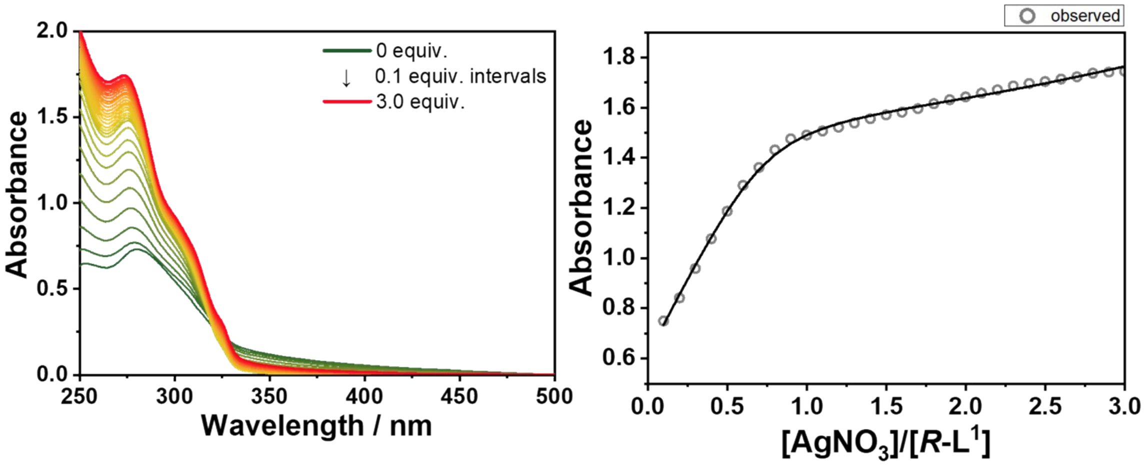

Fig. S33 Fitting of UV−vis titration data to determine the association constant for the formation of [*R*-**L**¹Ag]⁺ and [*R*-**L**¹$(AgNO_3)_2$] with HyperSpec softwareS1 by employing the 1:1 and 2:1 (Metal : L) binding model[5-7]: (a) UV-vis titration of *R*-**L**¹ (0.05 mM) with Ag^+ (0-3.0 equiv.) in DMSO : H_2O (1:1 v/v) and (b) HyperSpec output (circle: experimental points, solid line: theoretical fit).

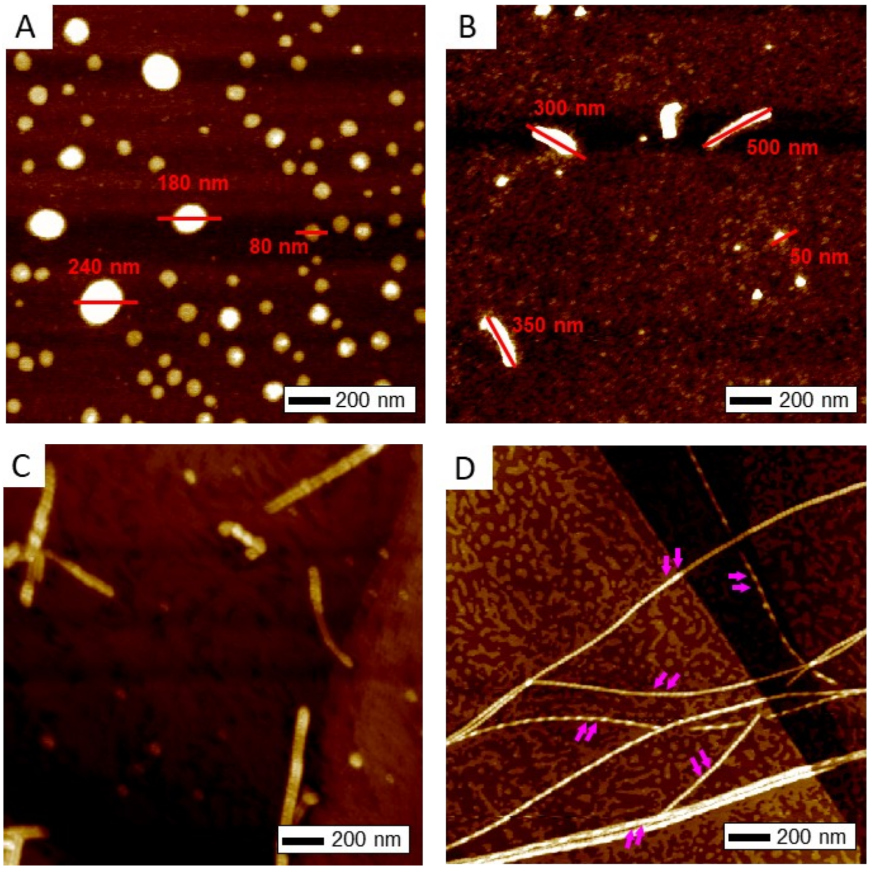

Fig. S34 Time-dependent AFM images of *R*-**L**¹ (6.4 mM) in the presence of 1.2 equiv. of $AgNO_3$ in DMSO/H_2O (1:1 v/v). Aging times: (A) 1 h, (B) 3 h, (C) 1 day, and (D) 3 days.

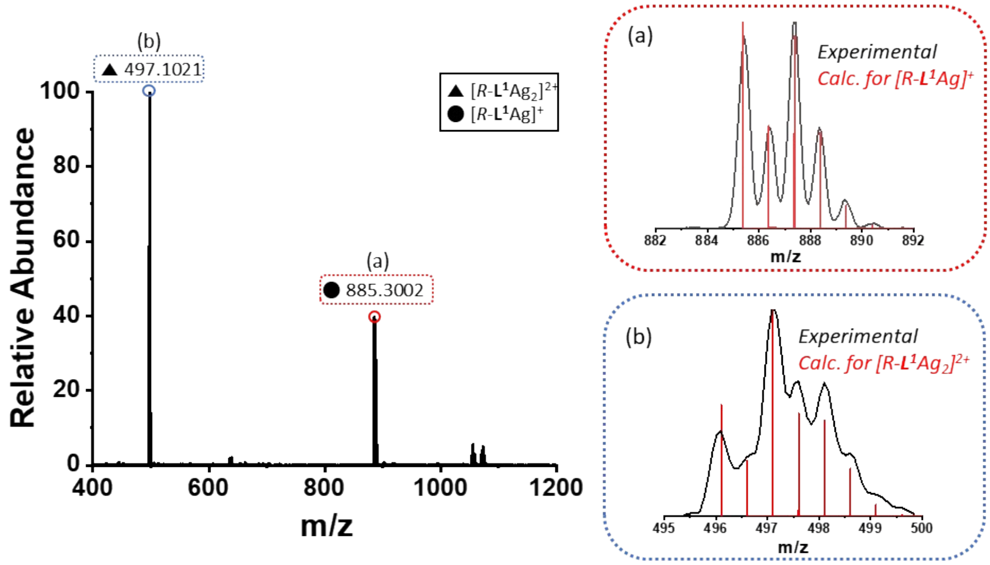

Fig. S35 ESI-MS spectrum of *R*-**L**[1] (6.4 mM) with 1.2 equiv. of $AgNO_3$ in DMSO/H_2O (1:1 v/v) after 1 h aging.

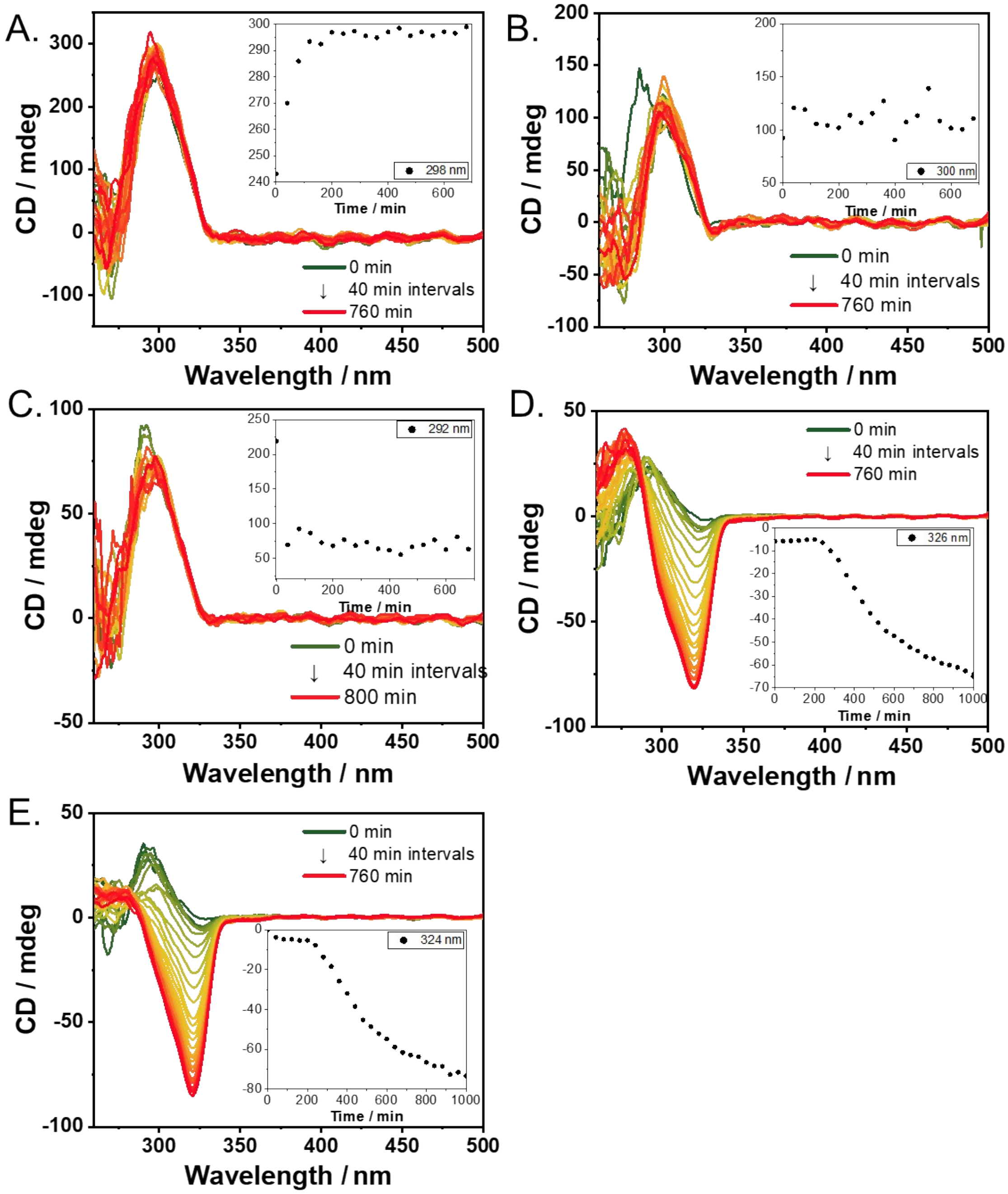

Fig. S36 Time-dependent (0-760 min) CD spectral changes of *R*-**L**[1] (8 mM) in the presence of $AgNO_3$ (0-1.4 equiv.) in DMSO/H_2O (1:1 v/v) at 20 °C (cell path length: 0.1 mm): (A) 0 equiv., (B) 0.4 equiv., (C) 0.8 equiv., (D) 1.2 equiv., and (E) 1.4 equiv.

[*Note*] To elucidate the supramolecular polymerization process, we first prepared the sample by dissolving *R*-**L**[1] (7.7 mM) in H_2O/DMSO (1:1 v/v). After adding the sample to the CD cell, it was heated to 90 °C (1 °C/min) to form the monomeric species in circular dichroism (CD) spectroscopy. Then the sample was cooled to 20 °C (5 °C/min) in CD spectroscopy. The time-dependent CD spectral changes were measured at 20 °C.

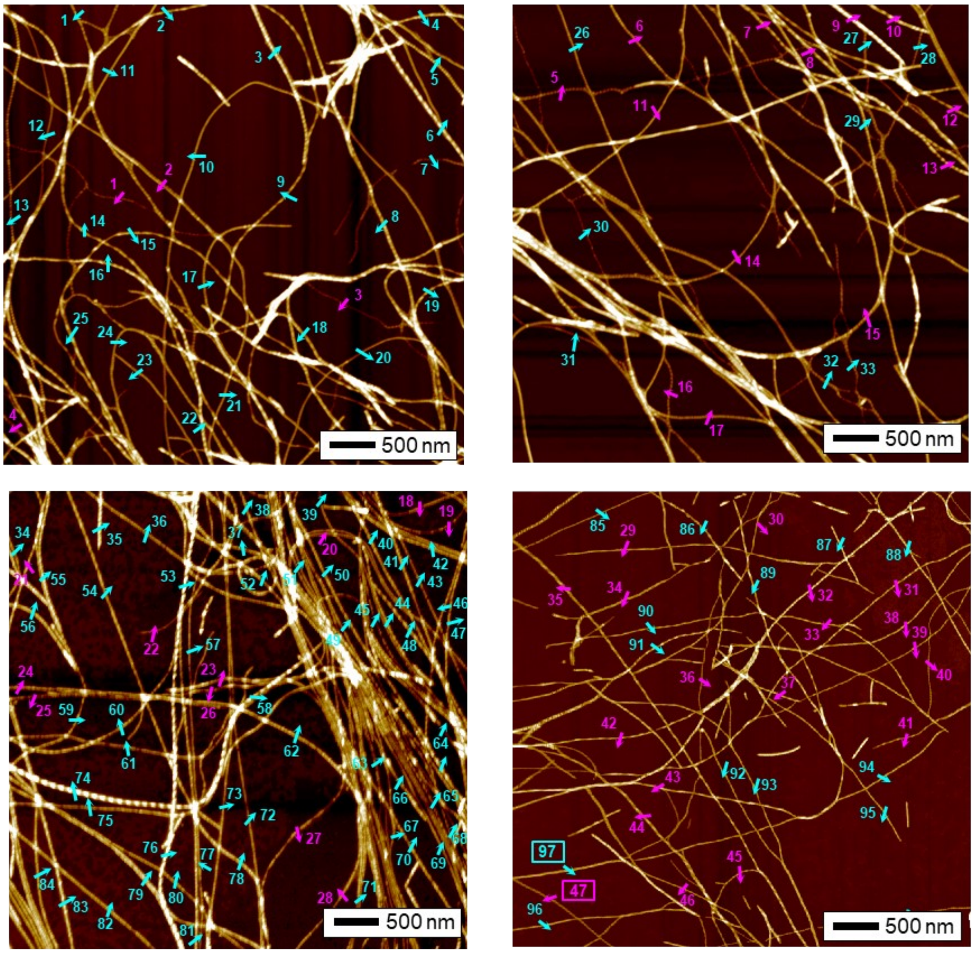

Fig. S37 AFM images of a mixed sample of aggregate I (7.2 mM, 50 μL) and aggregate III (7.2 mM, 21.5 μL) (Ag^+ : *R*-$\mathbf{L}^1$ molar ratio = 0.6) after 72 h aging. The blue arrows indicate right-handed helical fiber. The pink arrows indicate left-handed helical fiber.

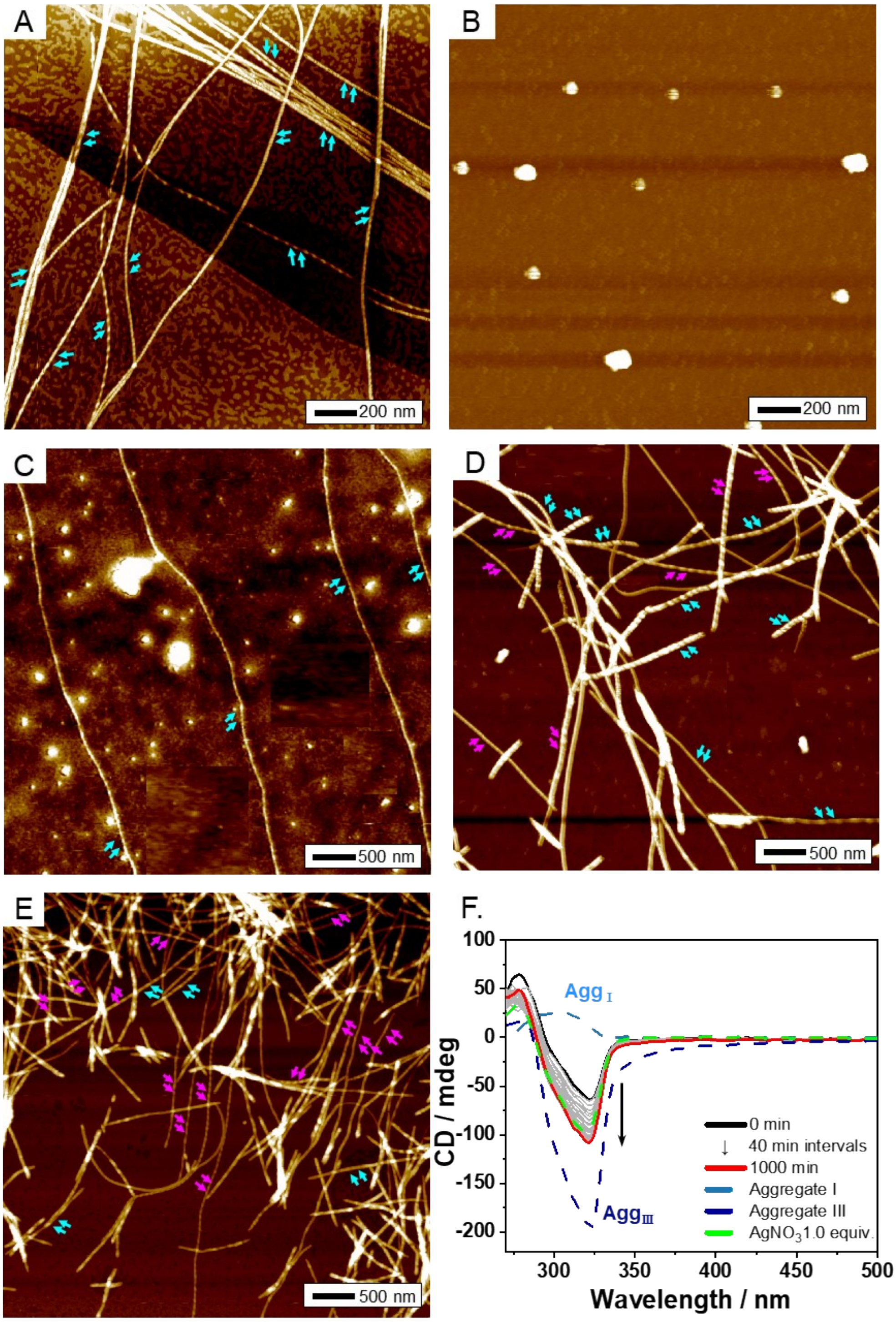

Fig. S38 AFM images of (A) aggregate I and (B) aggregate III. AFM image of a mixed sample of aggregate I (7.2 mM, 50 μL) and aggregate III (7.2 mM, 50 μL) after aging for (C) 10 min, (D) 1 h, and (E) 1 day. The molar ratio of Ag^+ to *R*-$\mathbf{L^1}$ in the mixed sample is 1.0 equiv. (F) Time-dependent CD spectra of the mixed sample of aggregate I (7.2 mM, 50 μL) and aggregate III (7.2 mM, 50 μL) in DMSO/H_2O (1:1 v/v). The blue arrows indicate right-handed helical fiber. The pink arrows indicate left-handed helical fiber.

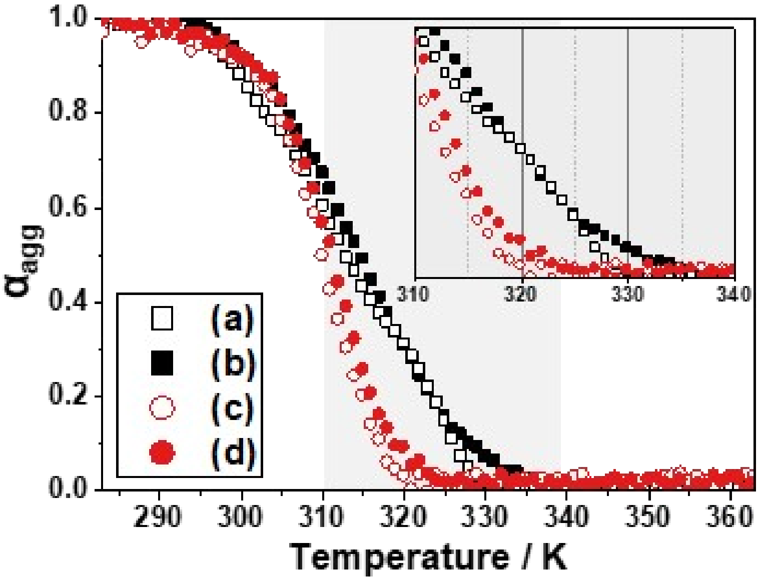

Fig. S39 Plot of CD spectral changes (at 323.5 nm) of *R*-**L**[1] vs temperature in the presence of different equiv. of $AgNO_3$: (a) 0.6 equiv. of $AgNO_3$ and (c) 1.2 equiv. of $AgNO_3$. (b) Aggregate I (7.2 mM, 50 μL) + aggregate III (7.2 mM, 21.5 μL), consisting 0.6 equiv. of $AgNO_3$. (d) Aggregate I (7.2 mM, 50 μL) + aggregate III (7.2 mM, 75 μL), consisting 1.2 equiv. of $AgNO_3$.

[*Note*] Temperature-dependent CD spectral changes were observed by heating with 1 °C/min.

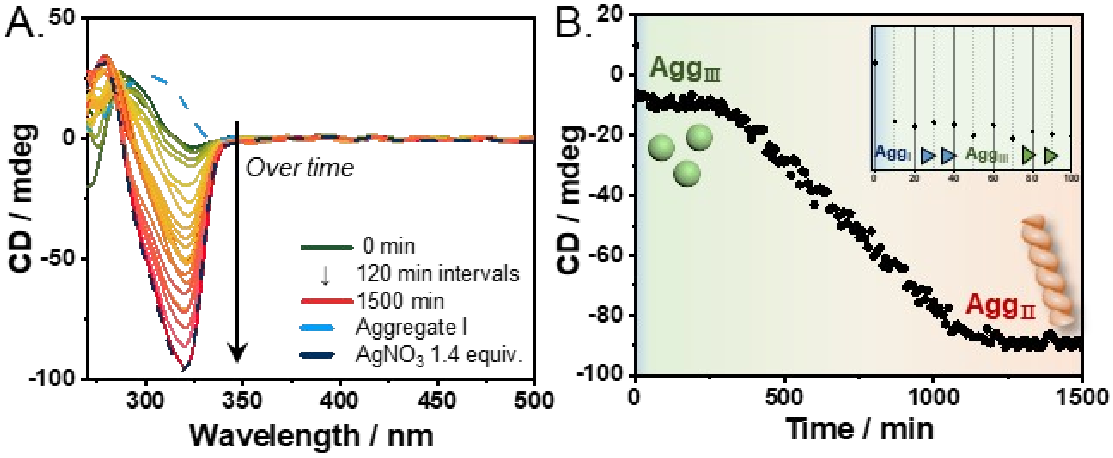

Fig. S40 (A) Time-dependent CD spectra of aggregate I upon addition of $AgNO_3$ (1.2 equiv.) in DMSO/H_2O (1:1 v/v). (B) Plot of CD spectral changes (at 323.5 nm) of aggregate I upon addition of $AgNO_3$ (1.2 equiv.) vs time.

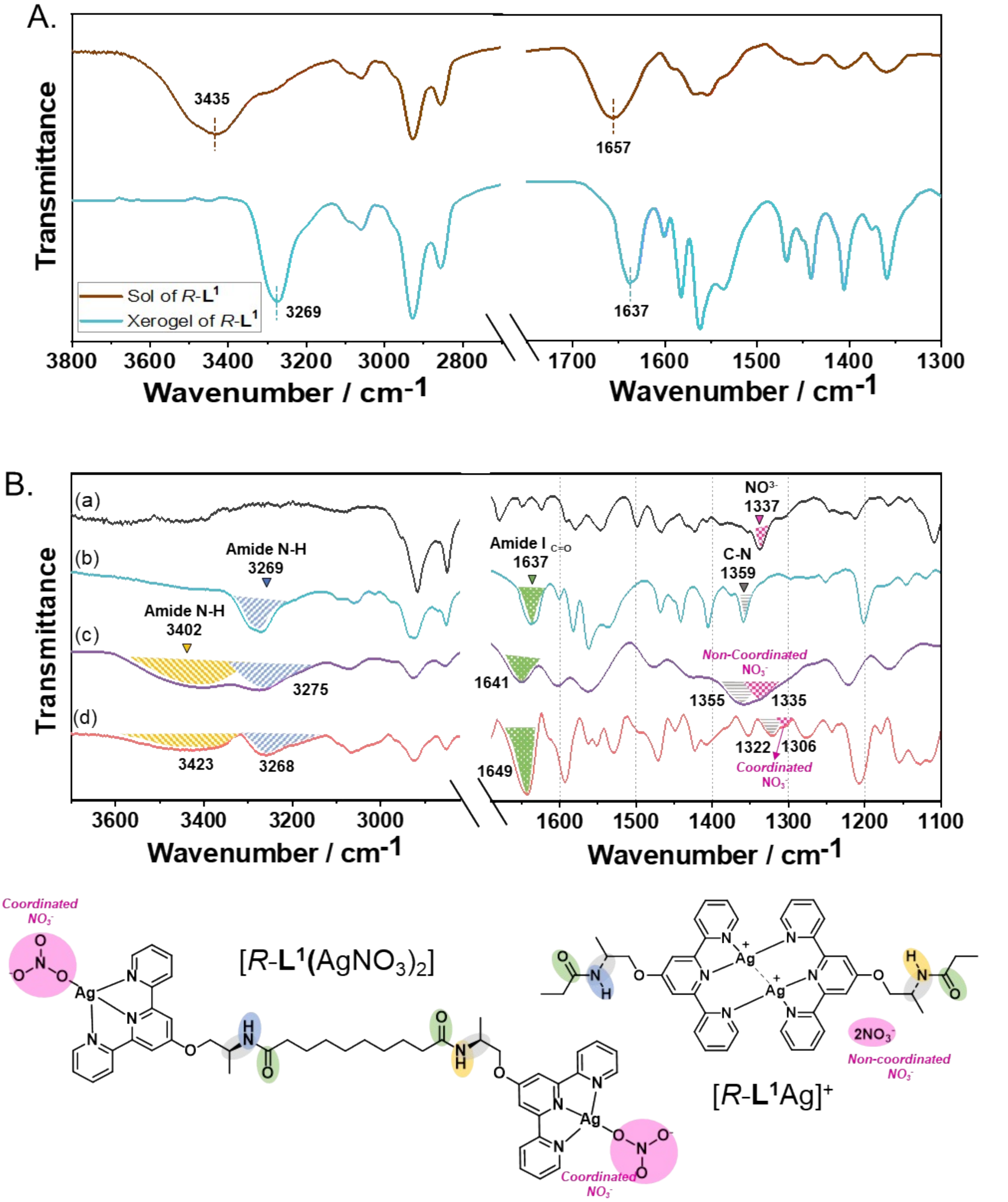

Fig. S41 (A) FT-IR spectra of sol state (brown line) of *R*-**L**[1] and aggregate I (blue line). (B) FT-IR spectra of (a) $AgNO_3$ (black line), (b) aggregate I (blue line), (c) aggregate II (purple line), and (d) aggregate III (pink line).

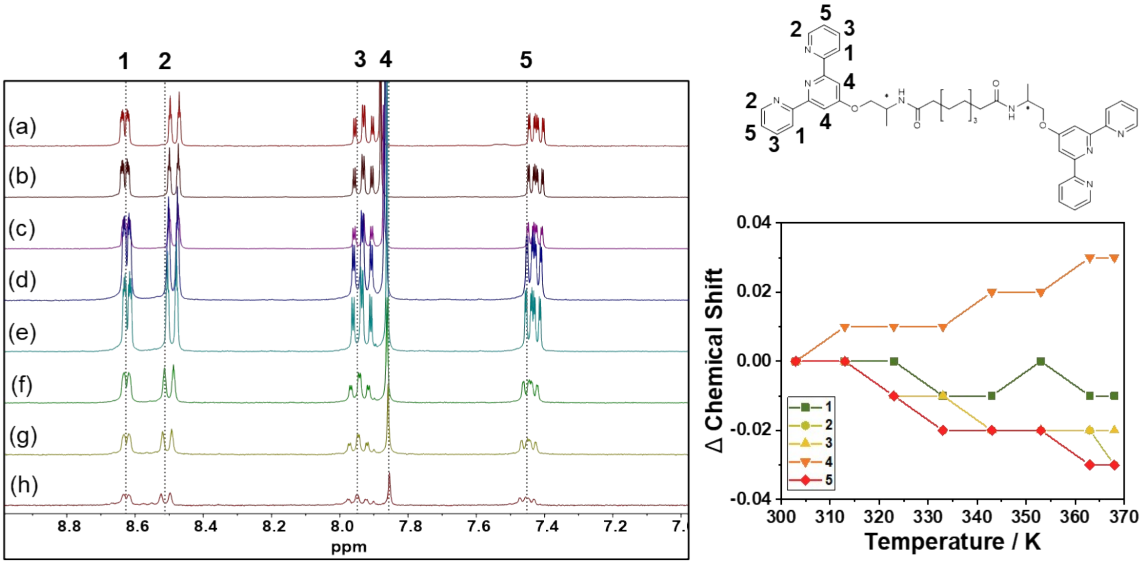

Fig. S42 Temperature-dependent ^{1}H NMR spectra of aggregate I in DMSO-d_6/D_2O (1:1 v/v); (a) 368 K, (b) 363 K, (c) 353 K, (d) 343 K, (e) 333 K, (f) 323 K, (g) 313 K, and (h) 303 K.

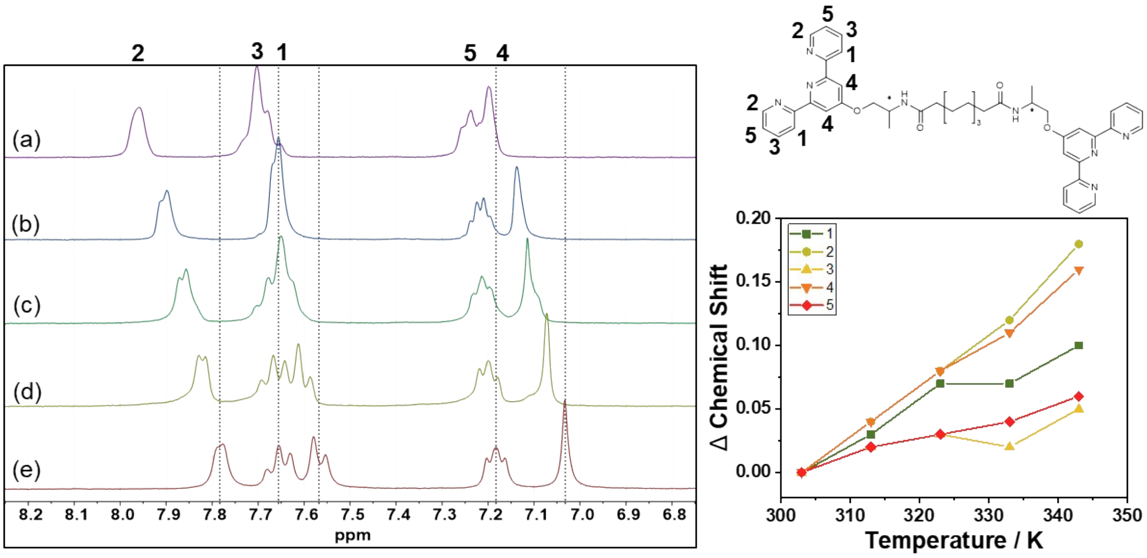

Fig. S43 Temperature-dependent ^{1}H NMR spectra of aggregate II in DMSO-d_6/D_2O (1:1 v/v); (a) 343 K, (b) 333 K, (c) 323 K, (d) 313 K, and (e) 303 K.

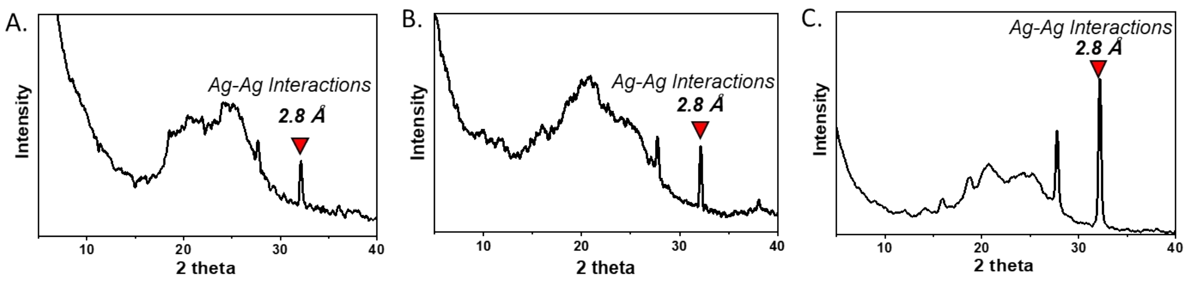

Fig. S44 WAXD patterns of *R*-$\mathbf{L}^1$ (6.4 mM) in the presence of different equiv. of $AgNO_3$ in DMSO/H_2O (1:1 v/v): (A) 0.5 equiv., (B) 0.8 equiv., and (C) 1.2 equiv.

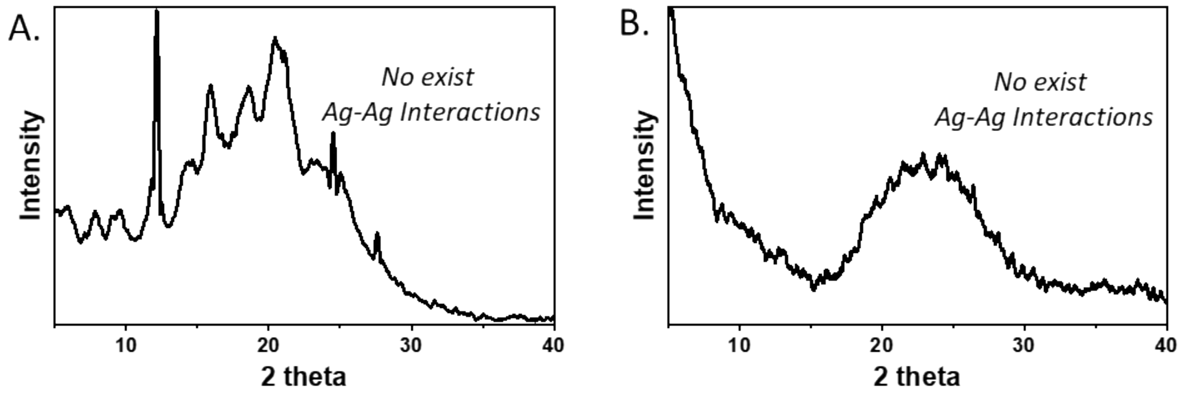

Fig. S45 WAXD patterns of *R*-$\mathbf{L}^1$ (6.4 mM) in the presence of different equiv. of $AgNO_3$ in DMSO/H_2O (1:1 v/v): (A) 0 equiv., (B) 2.0 equiv.

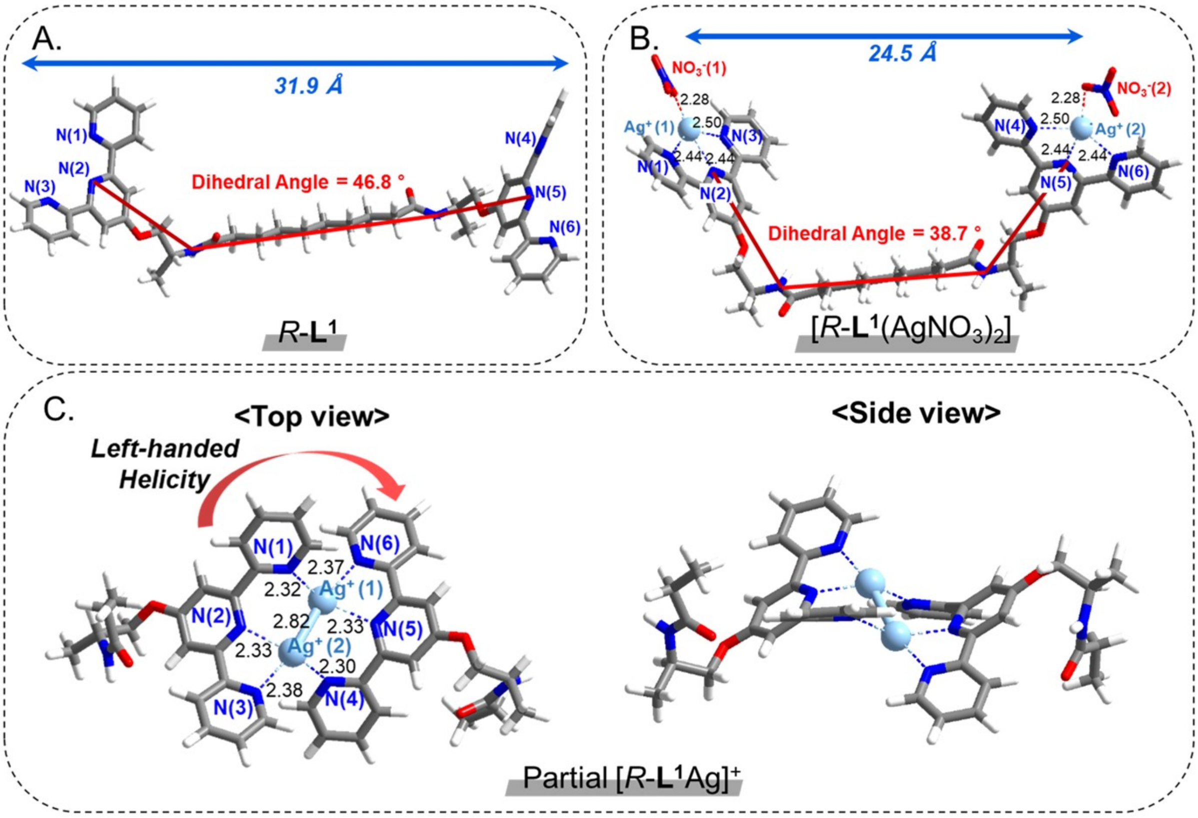

Fig. S46 DFT-optimized structures of (A) *R*-**L**[1], (B) [*R*-**L**[1]($AgNO_3$)$_2$], and (C) partial [*R*-**L**[1]Ag]$^+$. Note for the structure (C): Since NO_3^- remains uncoordinated in the FT-IR study, its coordination was ignored in the DFT calculation.

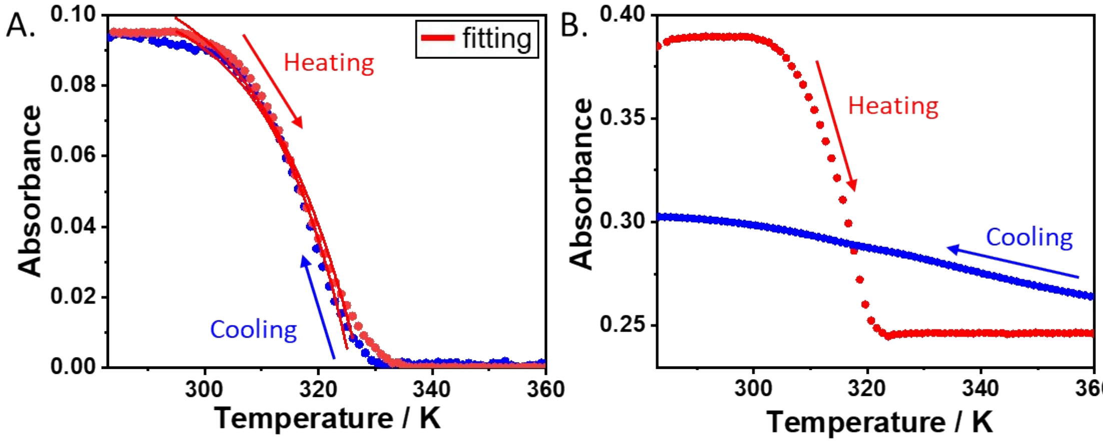

Fig. S47 Temperature-dependent absorption changes of (A) aggregate I and (B) aggregate II th rough heating (red points) and cooling (blue points).

[*Note*] In our repeated measurements of the cooling and heating curves for aggregate I (based on *R*-**L**[1]), no evidences of the hysteresis were found (Fig. S47A). During the heating and cooling, the T_e values are 327.2 and 325.7 K, respectively. While the T_m values are 317.5 and 316.9 K, respectively.

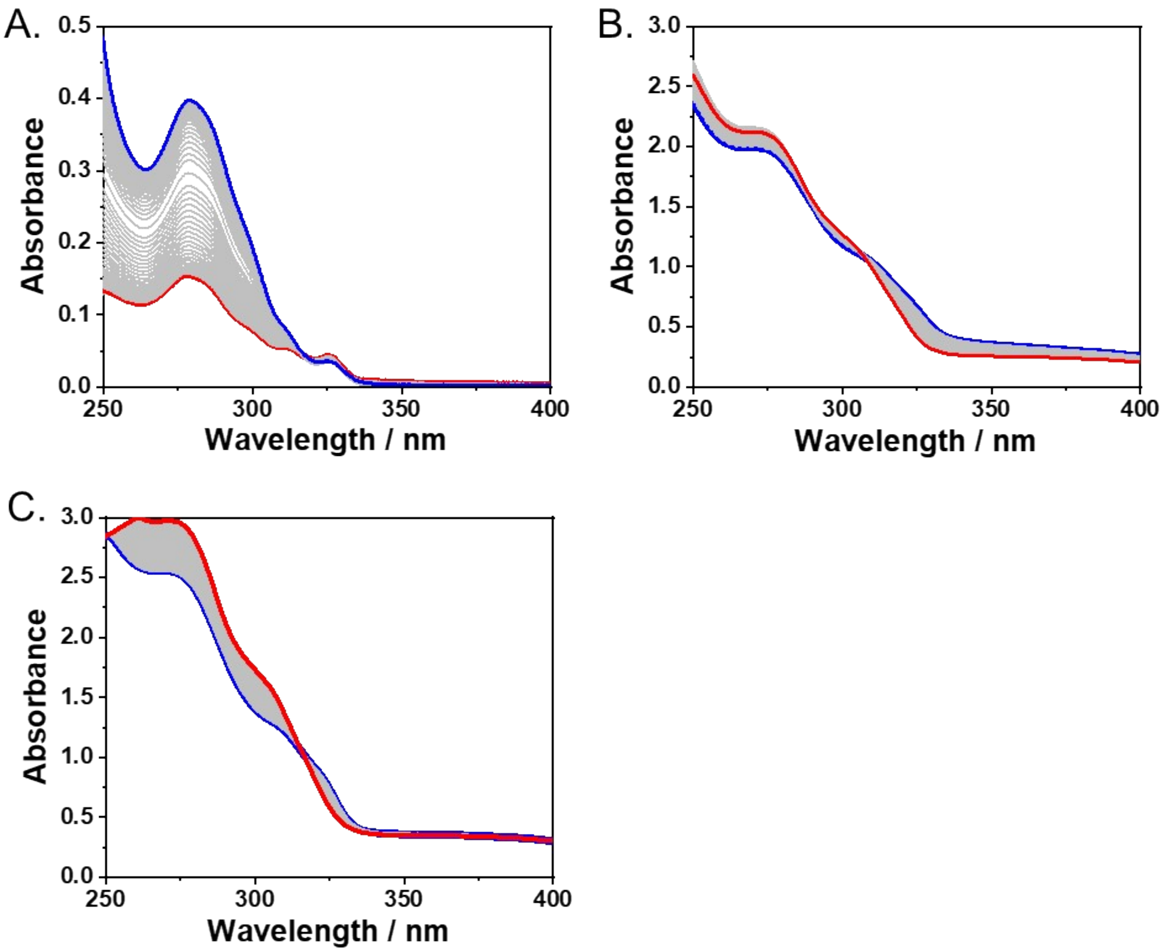

Fig. S48 Temperature-dependent UV spectra of different concentrations of *R*-**L**[1] with $AgNO_3$ (A) aggregate I, (B) aggregate II, and (C) aggregate III in DMSO/H_2O (1:1 v/v). The melting curves of aggregate II and aggregate III were measured by varying the aging time of the same sample (1.2 equiv. of $AgNO_3$). Aggregates I and II were measured after aging for 3 days, and aggregate III was measured after aging for 1 h.

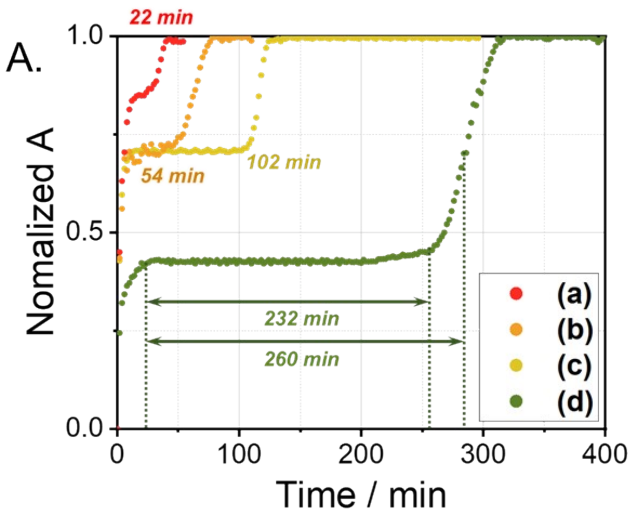

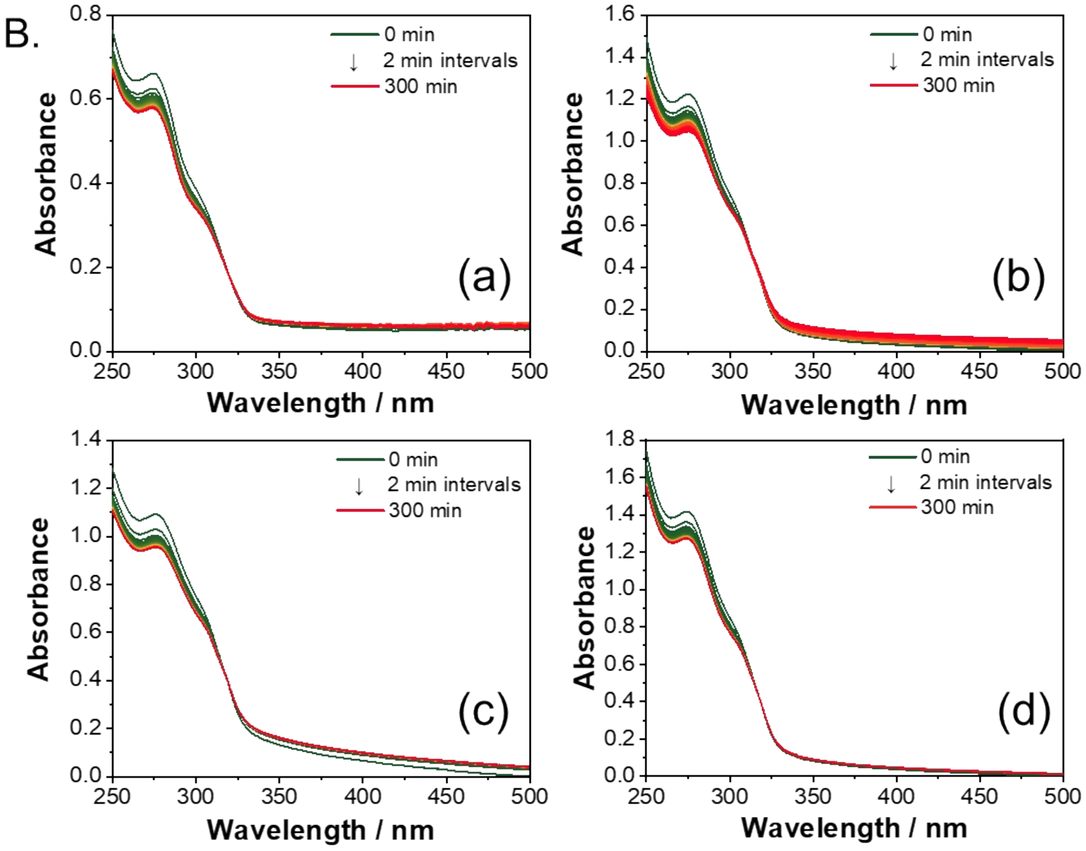

Fig. S49 (A) Time-dependent UV-vis spectral changes of different [*R*-**L**[1]] in the presence 0.6 equiv. of $AgNO_3$ in DMSO/H_2O (1:1 v/v): (a) 6.4 mM, (b) 6.8 mM, (c) 7.2 mM, and (d) 8.5 mM. (B: raw spectral data for the plots in A) Time-dependent UV-vis spectra of different [*R*-**L**[1]] in the presence 0.6 equiv. of $AgNO_3$: (a) 6.4 mM, (b) 6.8 mM, (c) 7.2 mM, and (d) 8.5 mM in DMSO/H_2O (1:1 v/v) at 20 °C (cell path length: 0.1 mm).

Table S1. Photochemical, helical, and morphology propeties of suprmolecular polymers based on *R*-**L**[1] without and with $AgNO_3$.

		$[R\text{-}L^1]_n$	$[R\text{-}L^1Ag]^+_n$	$[R\text{-}L^1(AgNO_3)_2]_n$
**UV-vis absorption**	**ILCT**	280 nm	285 nm	285 nm
	**MLCT**	*No MLCT*	318 nm, 326 nm	318 nm, 326 nm
**CD**		Positive(*P*-type)	Negative(*M*-type)	Negative(*M*-type)
**Morphology**		Right-handed helix 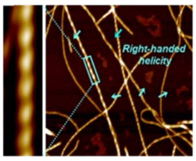	Left-handed helix 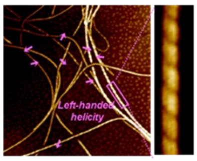	Spherical structure 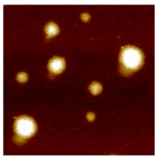
**Pitch**	**Angle**	*ca.* 28±3 °	*ca.* 30±3 °	
	**Length**	*ca.* 55±7 nm	*ca.* 75±5 nm	
**Height**		*ca.* 8 nm	*ca.* 5 nm	*ca.* 7.5 nm

Table S2. Cartesian coordinates of an optimized structure for *R*-**L**[1].

atom	x	y	z	atom	x	y	z
O	20.34365	-6.24608	-2.00909	C	1.82848	0.18669	1.81205
O	16.88319	-3.24205	-3.47785	C	2.18332	1.46574	2.24218
O	5.37778	-5.06214	-1.13148	C	1.18736	2.43657	2.35309
O	1.13451	-4.27374	-1.0479	C	-0.12463	2.07062	2.02935
N	23.26064	-10.4009	1.05595	H	24.12196	-4.10394	0.13602
N	23.56973	-5.77757	3.02392	H	25.47902	-2.67092	1.67436
N	22.71489	-7.64564	1.09797	H	19.15803	-3.6598	-3.72442
N	17.96563	-5.21253	-3.14253	H	19.88235	-4.28622	-1.49065
N	4.21376	-4.60544	-3.02156	H	21.25165	-4.47956	-2.63137
N	-3.80583	-2.66107	1.81472	H	20.7719	-5.01773	-5.09962
N	-1.12425	-1.88857	1.49042	H	19.92074	-6.5101	-4.6324
N	-0.47187	0.85201	1.62226	H	19.08667	-5.27804	-5.62215
C	20.94845	-10.7713	0.52017	H	17.89463	-6.16864	-2.81395
C	21.07487	-12.1268	0.82529	H	4.25495	-4.2617	-3.97384
C	22.31569	-12.6073	1.24543	H	19.98504	-10.361	0.21144
C	23.37382	-11.6958	1.34215	H	20.21353	-12.7952	0.7442
C	24.29709	-5.01334	3.83548	H	22.4646	-13.6599	1.49715
C	25.00267	-3.88141	3.41019	H	24.3643	-12.037	1.66815
C	24.93682	-3.53861	2.05937	H	24.32421	-5.31286	4.89056
C	24.17316	-4.33206	1.2025	H	25.58753	-3.29241	4.1204
C	23.50013	-5.45068	1.72657	H	20.615	-8.69953	-1.3483
C	22.07255	-9.93514	0.64809	H	21.88288	-4.70055	-0.33785
C	21.98778	-8.47208	0.33498	H	15.581	-4.9599	-1.51368
C	22.67405	-6.33847	0.84489	H	15.62528	-6.17039	-2.79773
C	21.89925	-5.77909	-0.19045	H	14.29999	-4.63596	-4.29306
C	21.18443	-8.01632	-0.71715	H	14.35478	-3.38244	-3.06216

C	21.13627	-6.64108	-0.98593	H	13.04705	-4.88261	-1.48968
C	20.25481	-4.86682	-2.35268	H	12.9918	-6.13273	-2.73082
C	19.79583	-5.42824	-4.79492	H	11.73036	-4.55833	-4.25189
C	19.28525	-4.73505	-3.52646	H	11.78502	-3.3163	-3.00345
C	16.85515	-4.41132	-3.1287	H	10.48578	-4.83502	-1.44941
C	15.58367	-5.08266	-2.61319	H	10.41689	-6.05913	-2.71475
C	14.30965	-4.47472	-3.20031	H	9.15861	-4.44568	-4.19695
C	13.02588	-5.03658	-2.58487	H	9.22764	-3.22861	-2.92398
C	11.74997	-4.41086	-3.15599	H	7.91604	-4.7642	-1.38844
C	10.4597	-4.96686	-2.54689	H	7.845	-5.96922	-2.6656
C	9.18543	-4.32022	-3.09807	H	6.52556	-4.37763	-4.10406
C	7.90058	-4.88119	-2.48405	H	6.69923	-3.12101	-2.87662
C	6.63091	-4.2164	-3.01678	H	1.80367	-4.55502	-4.3097
C	5.36908	-4.67828	-2.29019	H	2.36857	-6.24567	-4.16537
C	1.96519	-5.38893	-3.60401	H	0.98696	-5.67008	-3.1912
C	2.9145	-4.98419	-2.47964	H	3.08359	-5.84056	-1.80731
C	2.36364	-3.84446	-1.61278	H	2.20836	-2.93653	-2.22669
C	-4.9895	-3.0465	2.28536	H	3.0952	-3.61575	-0.82005
C	-5.29481	-4.36859	2.62978	H	-4.48126	-6.3769	2.73626
C	-4.29839	-5.33238	2.47033	H	-2.24926	-5.6641	1.86072
C	-3.05575	-4.93723	1.97443	H	-5.74722	-2.26084	2.39729
C	-2.84718	-3.58295	1.65371	H	-6.28363	-4.62813	3.01504
C	-0.77419	-3.93091	0.27204	H	1.82145	-1.76519	-0.16419
C	-1.52908	-3.11064	1.11894	H	-1.12569	-4.91176	-0.04969
C	0.45564	-3.46002	-0.20958	H	2.58106	-0.60102	1.73955
C	0.88411	-2.1896	0.19139	H	3.22152	1.69626	2.4956
C	0.05152	-1.44387	1.04878	H	1.41467	3.45197	2.68601
C	0.48109	-0.08269	1.50836	H	-0.93451	2.80704	2.10432

Table S3. Cartesian coordinates of an optimized structure for [*R*-**L**[1]($(AgNO_3)_2$)].

atom	x	y	z	atom	x	y	z
Ag	-1.82356	-1.66345	3.23628	C	-1.01212	-3.29874	0.48541
Ag	22.86432	-6.93279	3.31484	C	1.00908	-3.19914	-0.8307
O	23.66205	-6.22014	5.32723	C	1.60942	-2.48651	0.21666
O	24.78481	-7.25705	6.86734	C	0.84781	-2.22313	1.3673
O	24.24329	-8.29714	5.03959	C	1.41884	-1.46418	2.52765
O	-4.14505	-0.81871	4.02301	C	2.8001	-1.39567	2.77173
O	-4.32043	-0.31223	6.12736	C	3.25661	-0.67205	3.87465
O	-2.42121	-1.00313	5.33743	C	2.32865	-0.0449	4.70736
O	20.0495	-7.09945	-2.58035	C	0.97074	-0.17474	4.39951
O	17.18979	-5.05571	-5.78945	H	21.30362	-3.09877	-0.2439
O	5.6253	-4.90053	-2.50909	H	22.43026	-1.15751	0.8373
O	1.61313	-3.50397	-1.99266	H	19.41316	-5.59775	-5.47641
N	24.2482	-7.27334	5.77304	H	19.5613	-5.14423	-3.09624
N	-3.65974	-0.70043	5.17906	H	21.14635	-5.73041	-3.69961
N	21.87497	-9.09177	2.76091	H	21.20617	-7.35789	-5.69711
N	23.0669	-4.75383	2.11528	H	20.17326	-8.52404	-4.83692

N	21.55054	-6.85497	1.25045	H	19.67095	-7.89132	-6.43016
N	18.02277	-6.78249	-4.5694	H	17.80904	-7.5678	-3.96603
N	4.77561	-3.28406	-3.84921	H	4.97839	-2.51329	-4.47468
N	-3.11124	-2.98592	1.63649	H	19.52437	-10.3557	0.69223
N	-0.41966	-2.62476	1.48306	H	19.70895	-12.2571	2.29098
N	0.53862	-0.85712	3.33805	H	21.33148	-12.0837	4.21496
C	20.22956	-10.3045	1.52265	H	22.69257	-9.955	4.44223
C	20.32898	-11.3668	2.423	H	24.28637	-3.9521	3.57363
C	21.22194	-11.2734	3.4914	H	23.96746	-1.57456	2.79539
C	21.97818	-10.1041	3.62463	H	20.07633	-9.00497	-0.90412
C	23.65348	-3.71514	2.71186	H	21.22677	-4.89125	-1.47064
C	23.46615	-2.39758	2.28225	H	15.61197	-7.21192	-3.89387
C	22.61812	-2.17213	1.19716	H	15.29824	-7.2012	-5.63223
C	21.9959	-3.26061	0.58347	H	14.6567	-4.76326	-5.48965
C	22.24886	-4.55305	1.07099	H	14.85616	-4.81356	-3.74187
C	21.03279	-9.17036	1.72072	H	13.07183	-6.60378	-3.58409
C	21.01525	-7.99517	0.78874	H	12.85948	-6.53567	-5.33272
C	21.61712	-5.77389	0.47108	H	12.18601	-4.11259	-5.15674
C	21.1279	-5.77613	-0.84631	H	12.40017	-4.17757	-3.40908
C	20.49502	-8.08222	-0.50415	H	10.60667	-5.94273	-3.24936
C	20.54955	-6.95397	-1.33898	H	10.39467	-5.88848	-4.99795
C	20.09621	-6.01626	-3.50984	H	9.71989	-3.46827	-4.8477
C	20.16104	-7.63064	-5.48075	H	9.94902	-3.50668	-3.10048
C	19.42351	-6.47	-4.80485	H	8.14303	-5.25443	-2.88484
C	16.9969	-6.04179	-5.09687	H	7.93119	-5.25701	-4.63283
C	15.59546	-6.55793	-4.7831	H	7.18148	-2.86047	-4.55714
C	14.57606	-5.42936	-4.61551	H	7.52989	-2.80075	-2.82538
C	13.13661	-5.9235	-4.45412	H	2.56258	-2.03557	-4.85326
C	12.11988	-4.79066	-4.28579	H	2.80751	-3.58976	-5.70263
C	10.67469	-5.27052	-4.12467	H	1.45	-3.39243	-4.56204
C	9.66139	-4.13327	-3.96553	H	3.3247	-4.72842	-3.53972
C	8.22127	-4.61693	-3.78036	H	3.01121	-1.97987	-2.2281
C	7.21059	-3.47574	-3.64124	H	3.62615	-3.48915	-1.46598
C	5.81379	-3.97039	-3.27732	H	-4.96752	-5.67385	-0.42777
C	2.5008	-3.13302	-4.74927	H	-2.55076	-5.22148	-0.83144
C	3.38081	-3.62969	-3.60569	H	-4.86417	-2.62005	2.6531
C	2.95125	-3.08453	-2.23768	H	-6.18007	-4.34909	1.34438
C	-4.40494	-3.22376	1.86217	H	2.62981	-2.11921	0.13601
C	-5.12186	-4.18012	1.13538	H	-0.79683	-4.14063	-1.51762
C	-4.44821	-4.90726	0.15282	H	3.50881	-1.92169	2.13047
C	-3.09583	-4.6501	-0.07908	H	4.32688	-0.61183	4.0873
C	-2.45241	-3.66486	0.6864	H	2.64246	0.52533	5.58395
C	-0.32585	-3.61303	-0.68902	H	0.19469	0.2716	5.03033

Table S4. Cartesian coordinates of an optimized Structure for Partial [*R*-**L**^{1}Ag]$^{+}$.

atom	x	y	z	atom	x	y	z
Ag	3.15211	-1.25144	0.8467	C	10.45429	1.68764	-0.06042
Ag	3.58523	1.47762	1.45262	C	11.46025	2.55669	0.71412

O	-3.38266	1.31004	-0.8902	C	12.45217	3.20841	-0.25491
O	-2.9963	-0.93771	1.73704	C	10.41521	3.54984	2.83587
O	9.70799	0.79438	0.78368	C	11.06207	2.51053	3.74266
O	9.59967	4.3631	3.25108	C	10.64339	2.65922	5.20228
N	2.29449	-3.44141	0.48382	H	8.29636	3.16137	0.91461
N	-4.9286	-0.27001	-0.34558	H	8.3027	-1.05934	1.7876
N	0.89195	-1.0259	0.3258	H	-1.59251	-3.02439	1.48502
N	2.10177	1.64182	-0.29999	H	-1.59432	1.21538	0.74719
N	4.46445	3.6604	1.76068	H	3.0187	-2.77934	3.18868
N	5.8083	1.21141	2.10738	H	4.06318	-4.50345	0.27049
N	4.57955	-1.51131	2.65756	H	6.55903	-3.06399	5.6274
N	10.78145	3.56743	1.51174	H	7.56845	-1.27439	4.20485
C	0.94692	-5.83956	0.09141	H	4.1924	-3.80793	5.11777
C	2.34199	-5.80556	0.07322	H	3.65989	2.83781	-0.98752
C	2.9715	-4.57696	0.27514	H	2.30781	4.02725	-2.69555
C	0.24165	-4.65514	0.30713	H	-0.16032	3.48222	-2.87183
C	0.94922	-3.45871	0.50403	H	-5.56936	-0.95232	-0.73526
C	-3.25919	0.49714	-3.62149	H	-0.84803	-4.66144	0.29337
C	-4.38539	0.04747	-2.69756	H	-2.30499	0.01372	-3.35825
C	-4.17125	0.43147	-1.23578	H	-3.49089	0.24079	-4.66557
C	-6.02366	1.28859	1.26452	H	-3.11675	1.58568	-3.55863
C	-5.13786	0.04993	1.0702	H	-4.5477	-1.04164	-2.77019
C	-3.8379	0.21835	1.86659	H	-5.33849	0.51307	-3.00901
C	-1.09086	0.25512	0.79819	H	-5.6556	-0.82841	1.48766
C	-1.75931	-0.8939	1.25344	H	-5.54552	2.18163	0.83421
C	-1.07324	-2.12096	1.16767	H	-6.99531	1.14528	0.76976
C	0.2368	-2.15231	0.69402	H	-6.21654	1.47507	2.33256
C	0.21498	0.13724	0.31758	H	-4.09296	0.29712	2.9342
C	2.60255	2.59367	-1.1138	H	-3.31971	1.13024	1.55702
C	1.84126	3.27129	-2.06153	H	0.40969	-6.77677	-0.07119
C	0.80245	1.30128	-0.42567	H	2.93374	-6.70655	-0.09779
C	-0.03945	1.96871	-1.33486	H	-1.09502	1.69388	-1.40854
C	0.4841	2.9652	-2.1571	H	2.721	4.78272	1.82233
C	6.48559	2.31074	1.71296	H	3.88081	6.91949	2.41803
C	6.46983	0.03839	2.17957	H	6.38799	6.88216	2.71452
C	7.78372	-0.10154	1.73901	H	7.61303	4.73536	2.41255
C	7.79772	2.2502	1.23089	H	9.76849	2.31409	-0.65503
C	8.46354	1.01405	1.21032	H	10.99459	1.02389	-0.75077
C	3.80808	4.81186	1.94336	H	13.00602	2.45147	-0.82991
C	5.80415	3.63359	1.88798	H	11.93636	3.86803	-0.97322
C	6.53431	4.78058	2.23505	H	13.178	3.81757	0.30197
C	4.45648	6.00333	2.27394	H	12.01335	1.89266	1.39392
C	5.84277	5.97943	2.42913	H	10.40543	4.36691	1.006
C	4.0325	-2.4616	3.44206	H	12.15812	2.59537	3.63893
C	5.82943	-1.08954	2.93326	H	10.80908	1.50468	3.36306
C	6.56512	-1.64585	3.99385	H	9.55336	2.56393	5.31476
C	4.69421	-3.04651	4.51819	H	11.12886	1.89061	5.82159
C	5.99718	-2.63473	4.79462	H	10.92384	3.64688	5.59505

Table S5. Thermodynamic parameters for aggregate I, aggregate II, and aggregate III (*R*-**L**[1]: 7 mM) in mixed DMSO and H_2O (1:1 v/v).

	ΔG (kJ mol^{-1})	ΔH_e (kJ mol^{-1})	ΔS (J K^{-1} mol^{-1})	K_e (L mol^{-1})	T_e (K)
**Aggregate I**	-22.25	–89.6	–226	1.4×10^4	335.70
**Aggregate II**	-20.33	-155.22	-453	1.0×10^4	314.28
**Aggregate III**	-17.48	-171.81	-518	3.7×10^3	307.28

[a]Gibbs free energy. [b]Elongation enthalpy. [c]Entropy. [d]Elongation binding constant. [e]Elongation Temperature. The melting curves of aggregate II and aggregate III were measured by varying the aging time of the same sample ($AgNO_3$ 1.2 equiv.). Aggregates I and II were measured after aging for 3 days, and aggregate III was measured after aging for 1 hour.

4. Analytical data

4.1 ^{1}H-NMR and ^{13}C-NMR spectoscopy

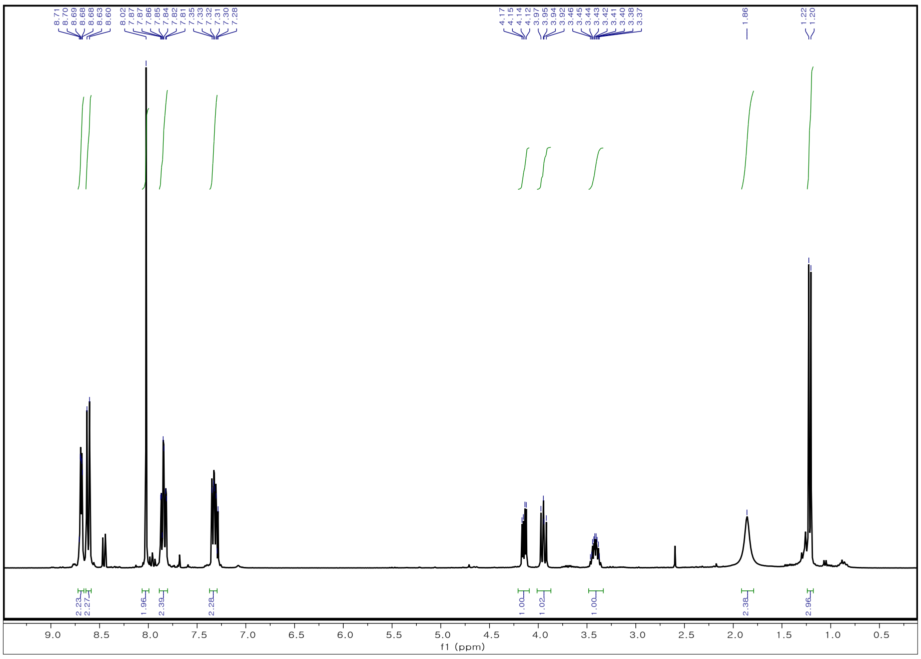

Fig. S51 ^{1}H NMR spectrum (300 MHz) of *R*-**L**^{2} in $CDCl_3$ at 25 °C.

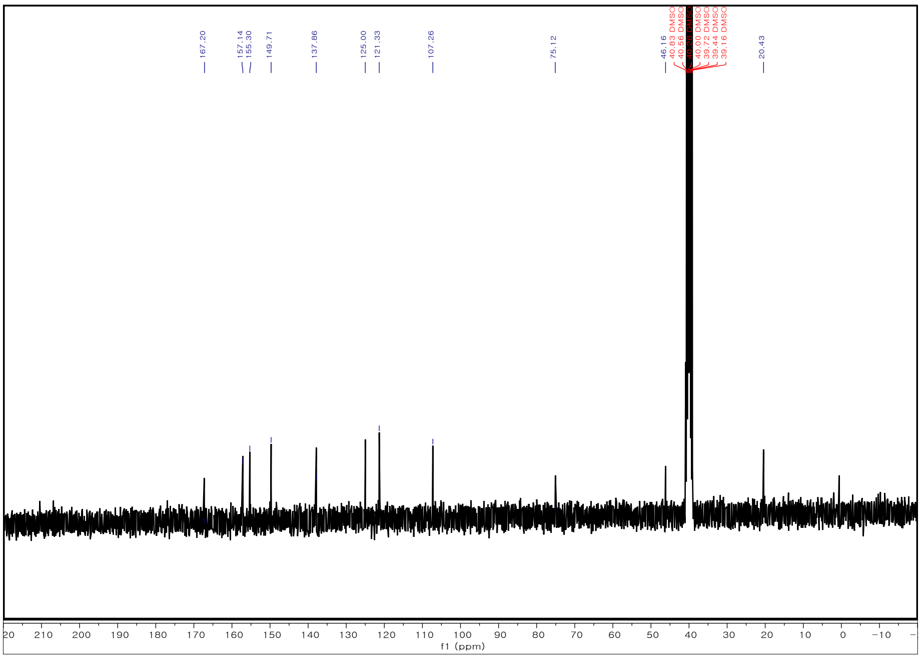

Fig. S52 ^{13}C NMR spectrum (75 MHz) of *R*-**L**^{2} in DMSO-d_6 at 25 °C.

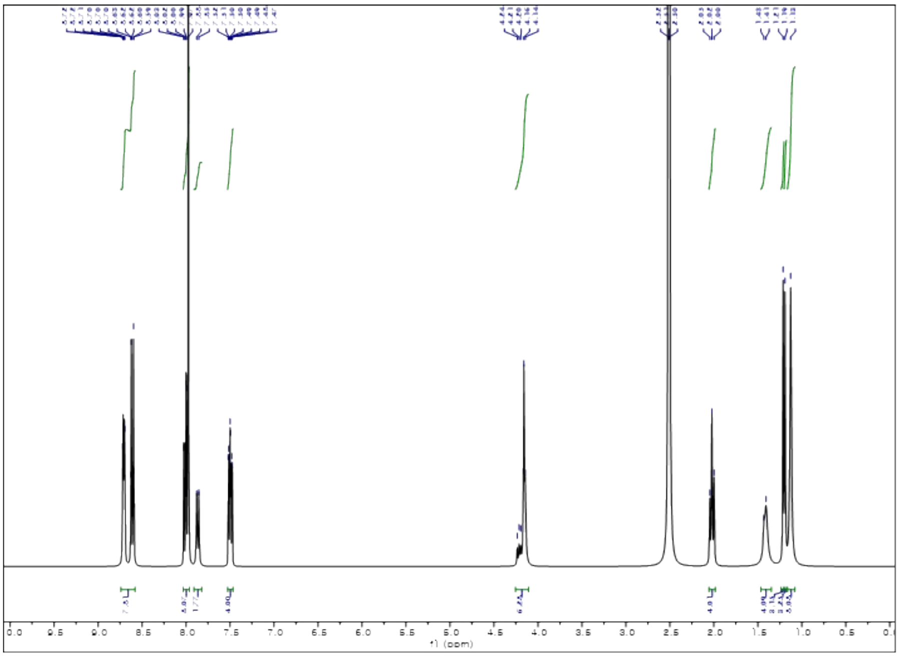

Fig. S53 ^{1}H NMR spectrum (300 MHz) of *R*-**L^1** in DMSO-d_6 at 25 °C.

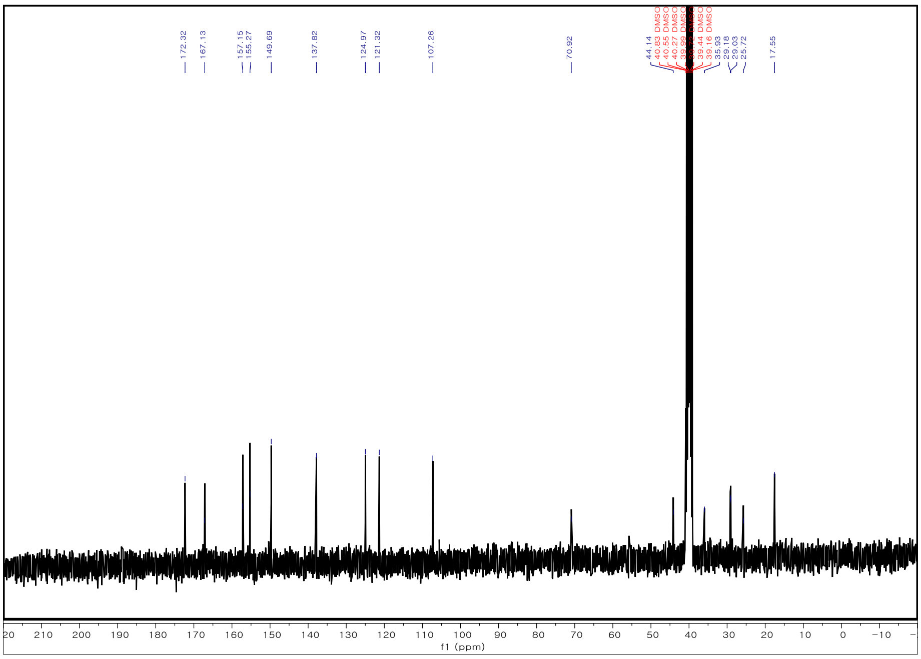

Fig. S54 ^{13}C NMR spectrum (75 MHz) of *R*-**L^1** in DMSO-d_6 at 25 °C.

4.2 HR mass spectrometry

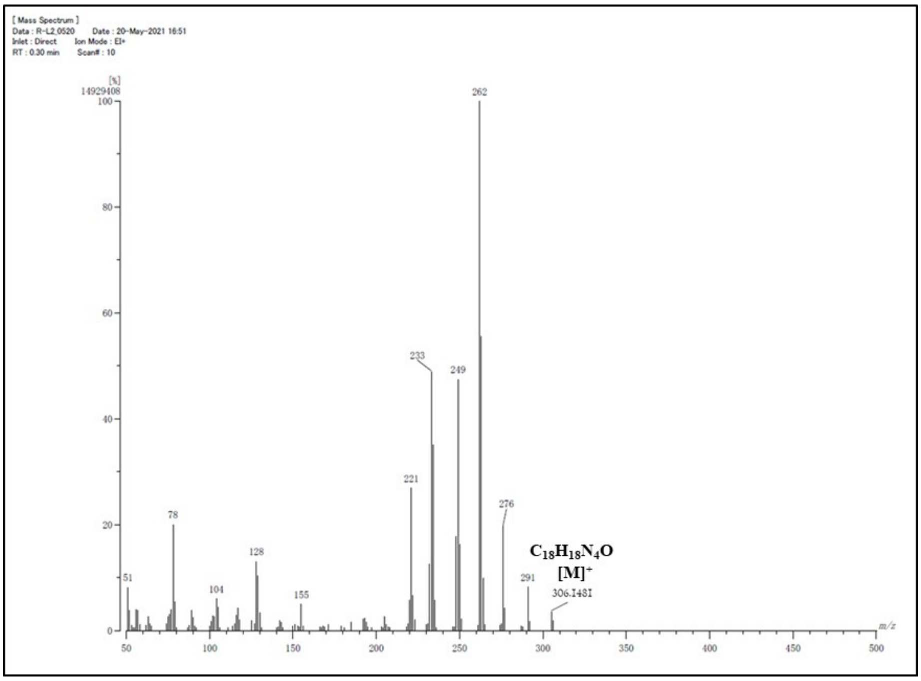

Fig. S55 HR EI-MS spectrum of *R*-**L**[2] in DCM.

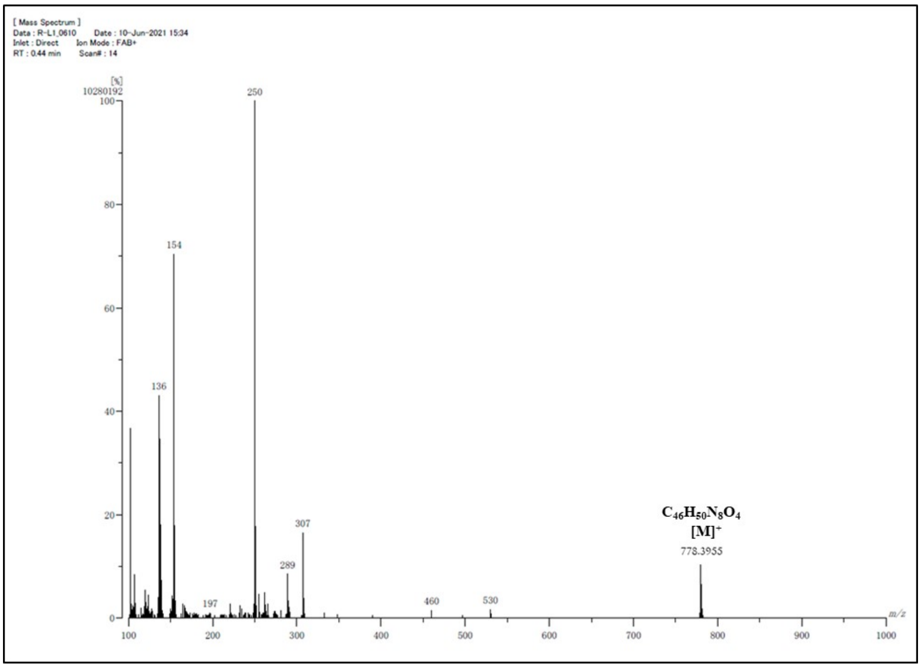

Fig. S56 HR FAB-MS spectrum of *R*-**L**[1] in DCM.

5. Supplementary references

1 H. M. M. ten Eikelder, A. J. Markvoort, T. F. A. de Greef and P. A. J. Hilbers, *J. Phys. Chem. B*, 2012, **116**, 5291-5301.
2 M. M. J. Smulders, M. M. L. Nieuwenhuizen, T. F. A. de Greef, P. van der Schoot, A. P. H. J. Schenning and E. W. Meijer, *Chem. Eur. J.*, 2010, **16**, 362-367.
3 H. Choi, S. Ogi, N. Ando and S. Yamaguchi, *J. Am. Chem. Soc.*, 2021, **143**, 2953-2961.
4 M. H.-Y. Chan, M. Ng, S. Y.-L. Leung, W. H. Lam and V. W.-W. Yam, *J. Am. Chem. Soc.*, 2017, **139**, 8639-8645.
5 http://www.hyperquad.co.uk/HypSpec2014.htm.
6 P. Gans, A. Sabatini and A. Vacca, *Talanta*, 1996, **43**, 1739-1753.
7 H. Ju, T. Abe, Y. Takahashi, Y. Tsuruoka, A. Otsuka, E. Lee, M. Ikeda, S. Kuwahara and Y. Habata, *Inorg. Chem.*, 2021, **60**, 1738-1745.
8 G. W. T. M. J. Frisch, H. B. Schlegel, G. E. Scuseria, M. A. Robb, J. R. Cheeseman, G. Scalmani, V. Barone, B. Mennucci, G. A. Petersson, H. Nakatsuji, M. Caricato, X. Li, H. P. Hratchian, A. F. Izmaylov, J. Bloino, G. Zheng, J. L. Sonnenberg, M. Hada, M. Ehara, K. Toyota, R. Fukuda, J. Hasegawa, M. Ishida, T. Nakajima, Y. Honda, O. Kitao, H. Nakai, T. Vreven, J. A. Montgomery, Jr., J. E. Peralta, F. Ogliaro, M. Bearpark, J. J. Heyd, E. Brothers, K. N. Kudin, V. N. Staroverov, R. Kobayashi, J. Normand, K. Raghavachari, A. Rendell, J. C. Burant, S. S. Iyengar, J. Tomasi, M. Cossi, N. Rega, J. M. Millam, M. Klene, J. E. Knox, J. B. Cross, V. Bakken, C. Adamo, J. Jaramillo, R. Gomperts, R. E. Stratmann, O. Yazyev, A. J. Austin, R. Cammi, C. Pomelli, J. W. Ochterski, R. L. Martin, K. Morokuma, V. G. Zakrzewski, G. A. Voth, P. Salvador, J. J. Dannenberg, S. Dapprich, A. D. Daniels, Ö. Farkas, J. B. Foresman, J. V. Ortiz, J. Cioslowski, and D. J. Fox, *Journal*, 2009.
9 J. P. Perdew, *Phys. Rev. B*, 1986, **33**, 8822-8824.
10 F. Weigend, *Phys. Chem. Chem. Phys.*, 2006, **8**, 1057-1065.
11 F. Weigend and R. Ahlrichs, *Phys. Chem. Chem. Phys.*, 2005, **7**, 3297-3305.
12 C. Kim, K. Y. Kim, J. H. Lee, J. Ahn, K. Sakurai, S. S. Lee and J. H. Jung, *ACS Appl. Mater. Interfaces*, 2017, **9**, 3799-3807.
